# Synthesis and Structure–Activity relationship of 1-(5-isoquinolinesulfonyl)piperazine analogues as inhibitors of *Mycobacterium tuberculosis* IMPDH

**DOI:** 10.1016/j.ejmech.2019.04.027

**Published:** 2019-07-15

**Authors:** Vinayak Singh, Angela Pacitto, Stefano Donini, Davide M. Ferraris, Sándor Boros, Eszter Illyés, Bálint Szokol, Menico Rizzi, Tom L. Blundell, David B. Ascher, Janos Pato, Valerie Mizrahi

**Affiliations:** a H3D Drug Discovery and Development Centre, Department of Drug Discovery and Development & Institute of Infectious Disease and Molecular Medicine, University of Cape Town, Rondebosch, 7701, Cape Town, South Africa; b MRC/NHLS/UCT Molecular Mycobacteriology Research Unit, DST/NRF Centre of Excellence for Biomedical TB Research & Wellcome Centre for Infectious Diseases Research in Africa, Institute of Infectious Disease and Molecular Medicine & Department of Pathology, University of Cape Town, Anzio Road, Observatory, 7925, South Africa; c South African Medical Research Council Drug Discovery and Development Research Unit, Department of Chemistry and Institute of Infectious Disease and Molecular Medicine, University of Cape Town, Rondebosch, 7701, Cape Town, South Africa; d Department of Biochemistry, University of Cambridge, 80 Tennis Court Road, Cambridge, CB2 1GA, United Kingdom; e Dipartimento di Scienze del Farmaco, University of Piemonte Orientale, Via Bovio 6, 28100, Novara, Italy; f Department of Biochemistry and Molecular Biology, University of Melbourne, Bio21 Institute, 30 Flemington Road, Parkville, 3052, Australia; g Vichem Chemie Research Ltd, Rákóczi utca 5, Veszprém, 8200, Hungary

**Keywords:** Tuberculosis, GuaB2, IMPDH, SAR, TB, tuberculosis, IMPDH, inosine-5′-monophosphate dehydrogenase, *Mth*, *Mycobacterium thermoresistible*

## Abstract

Tuberculosis (TB) is a major infectious disease associated increasingly with drug resistance. Thus, new anti-tubercular agents with novel mechanisms of action are urgently required for the treatment of drug-resistant TB. In prior work, we identified compound **1** (cyclohexyl(4-(isoquinolin-5-ylsulfonyl)piperazin-1-yl)methanone) and showed that its anti-tubercular activity is attributable to inhibition of inosine-5′-monophosphate dehydrogenase (IMPDH) in *Mycobacterium tuberculosis*. In the present study, we explored the structure–activity relationship around compound **1** by synthesizing and evaluating the inhibitory activity of analogues against *M. tuberculosis* IMPDH in biochemical and whole-cell assays. X-ray crystallography was performed to elucidate the mode of binding of selected analogues to IMPDH. We establish the importance of the cyclohexyl, piperazine and isoquinoline rings for activity, and report the identification of an analogue with IMPDH-selective activity against a mutant of *M. tuberculosis* that is highly resistant to compound **1**. We also show that the nitrogen in urea analogues is required for anti-tubercular activity and identify benzylurea derivatives as promising inhibitors that warrant further investigation.

## Highlights

• Forty-eight analogues of 1-(5-isoquinolinesulfonyl)piperazine were synthesized.
• Biochemical, whole-cell, and X-ray studies were performed to elucidate the IMPDH inhibition.
• Piperazine and isoquinoline rings were essential for target-selective whole-cell activity.
• Compound **47** showed improved IC_50_ against the *Mtb*IMPDH and maintained on-target whole-cell activity.
• Compound **21** showed activity against IMPDH in both wild type *M. tuberculosis* and a resistant mutant of compound **1**.

## Graphical abstract

**Figure:**
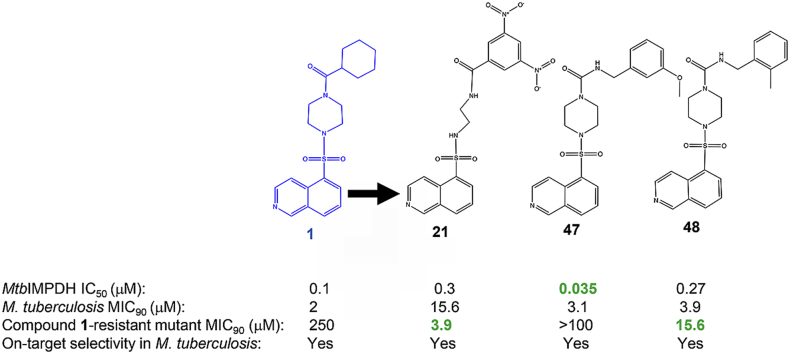

## 1 Introduction

Tuberculosis (TB), an infectious disease caused by *Mycobacterium tuberculosis* (*M. tuberculosis*), is the leading infectious killer globally, claiming 1.7 million lives in 2017 [1]. The severity of the global health threat presented by TB has been heightened by the emergence and spread of multi drug-resistant (MDR) and extensively drug-resistant (XDR) strains of *M. tuberculosis* that are resistant to first- and second-line drugs. Of the 10 million incident cases of TB recorded globally in 2017, approximately 458,000 were MDR, defined as resistant to isoniazid (INH) and rifampicin (RIF), with or without resistance to other first-line anti-tubercular drugs [1]. Of these, 8.5% had XDR-TB, which is resistant to INH and RIF (*i.e*., MDR-TB) in addition to any fluoroquinolone and at least one of the injectable second-line drugs, kanamycin, amikacin, or capreomycin [1]. Against this background, the “End TB” strategy, which aims to reduce TB deaths by 90% and TB incidence by 80% by 2035, was launched recently by the WHO (http://www.who.int/tb/strategy/en/). Among other interventions, new drug combinations that can shorten the duration of TB treatment; are effective against MDR and XDR-TB, and can be co-administered with drugs for the treatment of the major co-morbidities, HIV/AIDS and diabetes, are required to achieve this aspirational goal. While considerable progress has been made on establishing a TB drug pipeline, the high attrition rate of compounds in clinical development reinforces the need to fuel the pipeline with high-quality leads that act on novel targets [2].

In prior work conducted under the auspices of the More Medicines for Tuberculosis (MM4TB) consortium [3], we identified compound **1** (cyclohexyl(4-(isoquinoline-5-ylsulfonyl)piperazin-1-yl)methanone) as a promising new anti-tubercular agent with limited mammalian cell toxicity [4]. Compound **1** belongs to the 1-(5-isoquinolinesulfonyl) homopiperazine (Fasudil) scaffold, which is a known inhibitor of Rho-associated protein kinase (ROCK), but was found to inhibit inosine-5′-monophosphate dehydrogenase (IMPDH) in *M. tuberculosis*. IMPDH is an essential enzyme that catalyzes the NAD^+^-dependent conversion of inosine 5′-monophosphate (IMP) to xanthosine 5′-monophosphate (XMP) in the *de novo* purine biosynthesis pathway [5]. Compound **1** is one of the several small-molecule inhibitors of *M. tuberculosis* IMPDH discovered in recent years [[6], [7], [8], [9], [10], [11], [12], [13], [14], [15]]. IMPDH was genetically validated as a vulnerable and bactericidal drug target, and compound **1** was shown to be active against *M. tuberculosis* in macrophages. Moreover, in a parallel study, the value of IMPDH as a new TB drug target was called into question by the lack of *in vivo* efficacy of an IMPDH inhibitor from an indazole sulfonamide series [8]. This conclusion was attributed to subversion of IMPDH essentiality *in vivo* via the purine salvage pathway, which enables GMP production in *M. tuberculosis* by assimilation of exogenous guanine, a metabolite found to be present at millimolar concentrations in normal and diseased tissue from humans and rabbits [8]. However, this notion was questioned by Hedstrom [16] who offered alternative explanations for the discrepant conclusions regarding the value – or otherwise – of IMPDH as a new TB drug target. Importantly, Hedstrom and colleagues recently reported a benzoxazole-based *M. tuberculosis* IMPDH inhibitor which retains activity in the presence of exogenous guanine [14], suggesting that IMPDH may indeed be a vulnerable target in *M. tuberculosis* [5].

In the present study, we set out to explore the structure-activity relationships (SAR) around compound **1** with the aim of identifying more potent analogues with improved activity against a compound **1**-resistant mutant of *M. tuberculosis* to enable further pharmacologic interrogation of this target. The compounds were tested for inhibitory activity against the IMPDH enzyme and for whole-cell activity against replicating wild-type *M. tuberculosis* strain H37Rv. Target selectivity in *M. tuberculosis* was assessed by testing a subset of compounds for activity against a conditional knockdown mutant in which the level of IMPDH can be progressively depleted by transcriptional silencing of the IMPDH-encoding gene, *guaB2* [5]. Cross-resistance was assessed by testing for activity against a mutant of *M. tuberculosis*, SRMV2.6 [5], which shows high-level resistance to compound **1** as a result of a Tyr487Cys substitution. As the crystal structure of *Mycobacterium thermoresistible* (*Mth*) IMPDH (GuaB2) was elucidated [5] during the course of this study, the modes of binding of seven compounds to *Mth* IMPDH were also analysed by X-ray crystallography and docking analyses performed for three others.

## 2 Results and discussion

### 2.1 Design strategy for target compounds

**Fig. 1:**
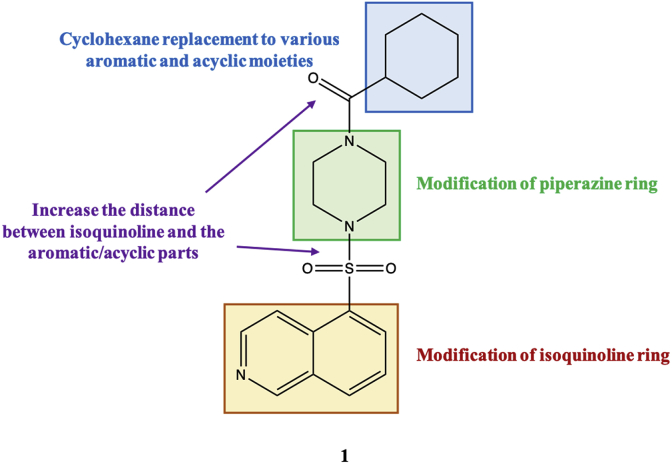
Design of target compound series derived from compound **1**.

**Scheme 1:**
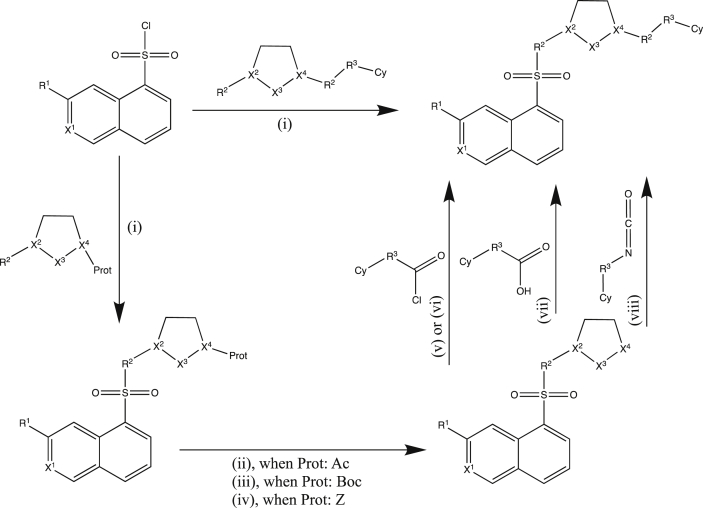
The synthetic pathway of derivatives of compound **1**. Reagents and conditions: (i), EtOAc, triethylamine, ambient temp; (ii), 2N HCl, 3 h reflux; (iii), TFA/DCM = 1:1, 3 h, ambient temp; (iv), H_2_/Pd—C, EtOH, overnight, ambient temp; (v), Py, ambient temp; (vi), EtOAc, aq.NaHCO_3_, overnight, ambient temp; (vii), EtOAc/DMF, carbodiimide (DCC or EDCI) overnight, ambient temp; (viii), Py, 4 h, 100 ^°^C.

**Scheme 2:**
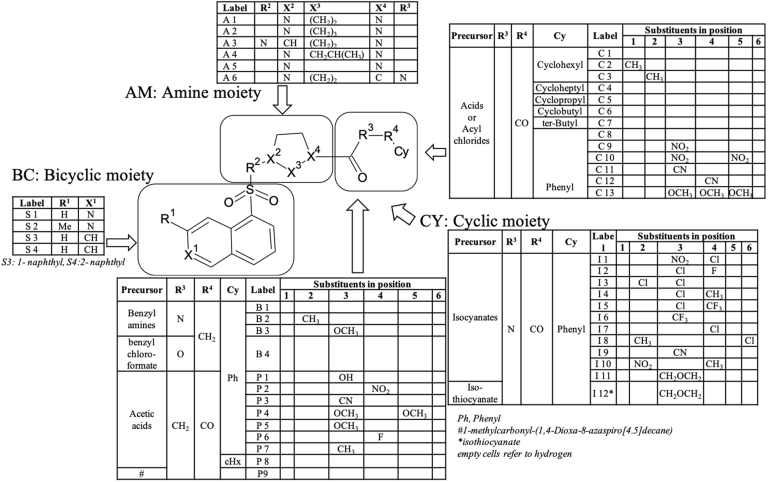
Synthesis of derivatives of compound **1** (also see Table S1).

To understand the SAR, a molecular library of 49 compounds was built around the compound **1** structure (Fig. 1; Scheme 1, Scheme 2; Tables S1 and S2). We made changes on the Fasudil-like moiety; on the cyclohexyl ring, utilizing both alicyclic and aromatic substituents; and by increasing the distance between the carbonyl group and the cyclohexyl ring to produce phenylacetic acid, phenylurea, and benzylurea derivatives.

### 2.2 Synthetic chemistry

The synthetic routes for the preparation of 1-(5-isoquinolinesulfonyl)piperazine derivatives (2-49) derivatives are summarized in Scheme 1, Scheme 2 and Table S1.

### 2.3 SAR profiling using biological assays

#### 2.3.1 Set I: modifications on the Fasudil-like moiety

The finding by Magnet et al. [4] that the Fasudil analog, 5-(piperazin-1-ylsulfonyl)isoquinoline, has no MIC against *M. tuberculosis*, pointed to a critical role for the cyclohexyl group in the activity of compound **1** – a notion substantiated subsequently by the co-crystal structure of compound **1** bound to *Mth* IMPDH [5]. Therefore, in the first set of compounds, we retained the cyclohexyl and modified the Fasudil-like moiety to produce compounds **2**–**8** (Table 1). Replacement of the rigid piperazine ring with the flexible ethylenediamine spacer resulted in the loss of whole-cell activity for compound **2** while retaining activity against the enzyme. Modification of the piperazine ring by inclusion of a methyl group at position-3 (compound **3)** resulted in profound loss of both biochemical and whole-cell activities (MIC_90_: 100 μM, IC_50_: >100 μM). Increasing the distance between the sulfonamide and carboxamide groups (compound **4** and **5**) also resulted in the loss of both whole-cell activity and activity against the enzyme. All other modifications of the piperazine ring similarly ablated whole-cell activity while significantly reducing activity against the enzyme. The introduction of a methyl group at position-3 of the isoquinoline ring (compound **6**) ablated both whole-cell and enzyme activities (MIC_90_: 100 μM, IC_50_: >100 μM) (Table 1). The isoquinoline substituent was then replaced by a naphthalene to produce the 1-naphthyl derivative, **7**, or 2-naphthyl derivative, **8**. While these compounds retained modest activity against the enzyme, both modifications completely ablated whole-cell activity (Table 1), suggesting an essential role for the nitrogen atom in the isoquinoline moiety for compound uptake.

**Table 1:**
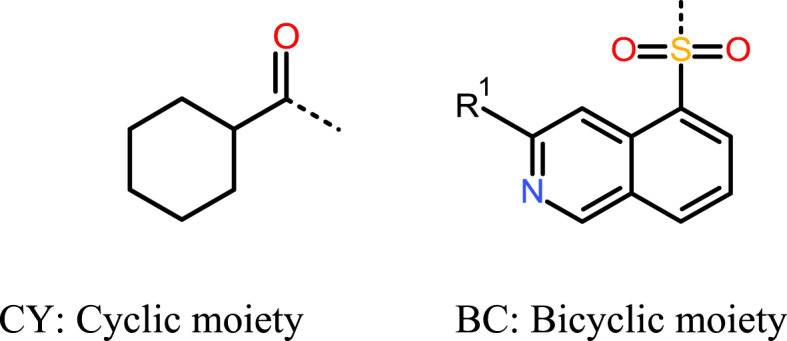
SAR profiling of modifications on the Fasudil-like moiety.

Cmpd No.	Structure X = N R^1^ = H if not defined otherwise	IMPDH IC_50_ (μM)	MIC_90_ (μM) against WT	MIC_90_ (μM) against SRMV2.6	Hyper-sensitivity to *guaB2* cKD
**1**		0.1 ± 0.01	2	250	∼32-fold
**2**		1.5 ± 0.02	50	>250	ND^a^
**3**		>100	100	100	ND
**4**		9.7 ± 0.3	>100	>100	ND
**5**		14.7 ± 0.3	>100	>100	ND
**6**		>100	>100	>100	ND^a^
**7**		3.0 ± 0.1	>100	>100	ND
**8**		1.6 ± 0.1	>100	>100	ND

a ND, not determined.

#### 2.3.2 Set II: modifications on compound **2**

Based on the data obtained from the compounds in Set I, the ethylenediamine compound **2** was selected for further development. We explored the effect of ring size and found a correlation with the inhibitory activity against the enzyme, with the IC_50_ value of **9** > **10** > **2** (Table 2). The cyclobutyl (**10**) and t*.*-butyl (**11**) analogues showed similar activity profiles presumably owing to their comparable size and polarity. However, all of the ethylenediamine analogues had weak activity against *M. tuberculosis* (MIC_90_: 50–100 μM) underscoring the importance of the piperazine ring for whole-cell activity.

**Table 2:**
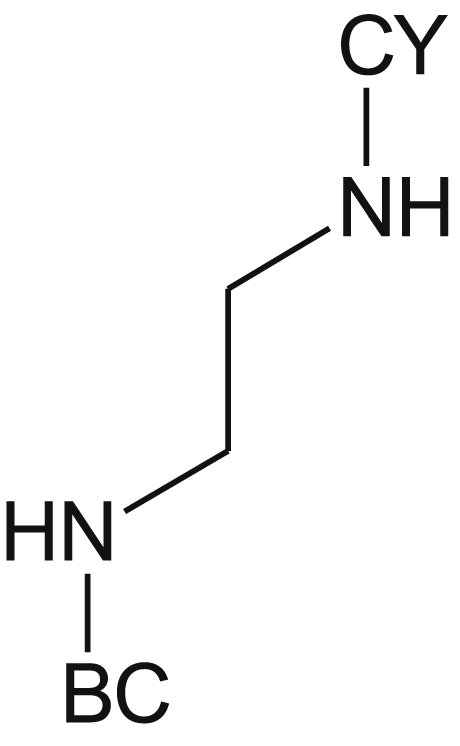
SAR profiling of compound 2.

Cmpd No	CY	IMPDH IC_50_ (μM)	MIC_90_ (μM) against WT	MIC_90_ (μM) against SRMV2.6	Hyper-sensitivity to *guaB2* cKD
**2**		0.5 ± 0.02	50	>250	ND^a^
**9**		6.9 ± 0.2	100	100	ND
**10**		2.7 ± 0.1	100	100	ND
**11**		2.8 ± 0.2	100	100	ND

a ND, not determined.

#### 2.3.3 Set III: modifications on the cyclohexyl ring

As reported previously, the cyclohexyl group of compound **1** was orientated to form strong pi interactions with the Y487’ (Y471’ in the *Mth* structure) side chain from the adjacent protomer in the IMPDH tetramer [5]. To explore the SAR around the cyclohexyl ring, compounds **12**–**21** were synthesized and analysed in terms of IMPDH inhibition and whole-cell activity (Table 3).

**Table 3:**
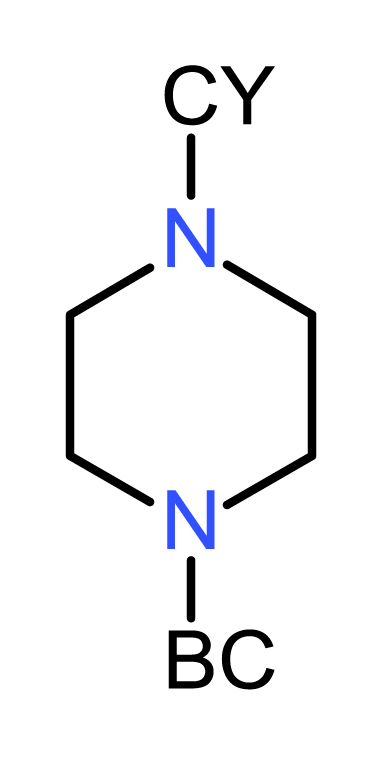
SAR profiling of modifications on the cyclohexyl ring.

Cmpd No	CY	IMPDH IC_50_ (μM)	MIC_90_ (μM) against WT	MIC_90_ (μM) against SRMV2.6	Hyper-sensitivity to *guaB2* cKD
**1**		0.1 ± 0.01	2	250	∼32-fold
**12**		2.4 ± 0.01	250	>250	ND^a^
**13**		0.084 ± 0.0	31.2	>250	16–32-fold
**14**		0.83 ± 0.04	31.2	>250	16-fold
**15**		8.1 ± 0.1	>100	>100	ND
**16**		8.6 ± 0.5	25	50	ND
**17**		16.5 ± 0.5	100	100	ND
**18**		1.1 ± 0.1	12.5	50	8-fold
**19**		1.4 ± 0.01	12.5	100	ND
**20**		12.0 ± 0.5	15.6	>125	ND
**21**		0.3 ± 0.02	15.6	3.9	4-fold

a ND, not determined.

##### 2.3.3.1 IIIa: alicyclic derivatives

**Fig. 2:**
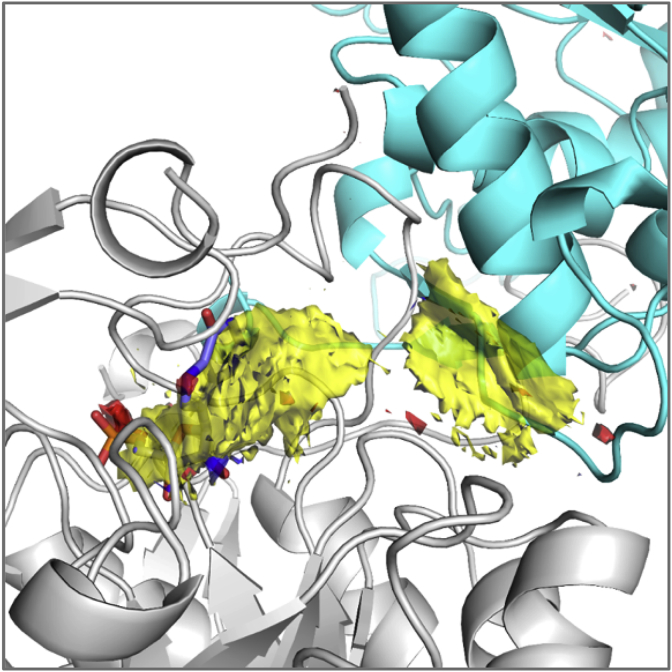
Hotspot maps of IMP bound IMPDH. Calculated hydrogen donor regions are coloured in blue, hydrogen acceptor regions in red and apolar regions in yellow.

**Fig. 3:**
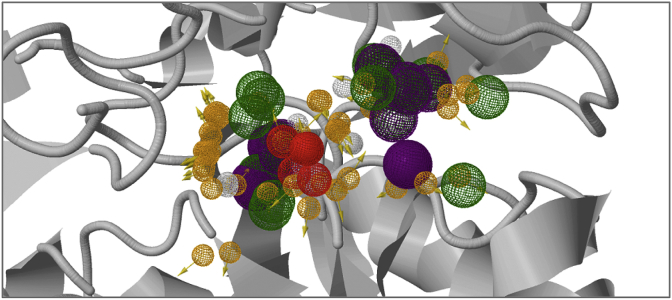
Pharmacophore model of the NAD-binding pocket, showing the important interactions made by our recently described inhibitors (including 5J5R [5], 5K4X and 5K4Z [8]). The chemical characteristics of the small molecule binders are represented by mesh spheres, with green representing hydrogen donors and positive ions, purple aromatic groups, white and orange hydrogen donors and acceptors respectively, and blue and red positive and negative ions respectively.

**Fig. 4:**
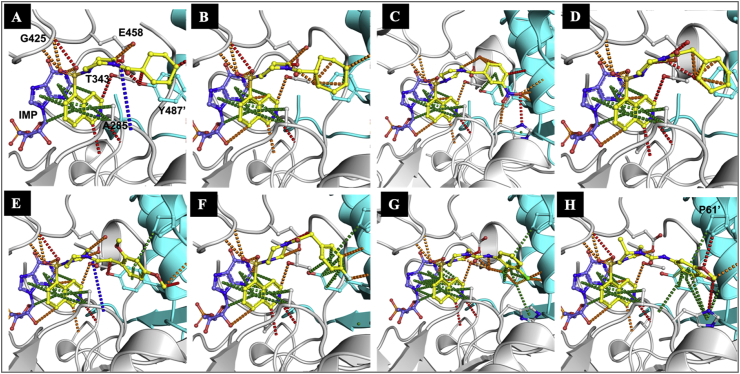
The X-ray crystal structures of compounds bound to *Mth* IMPDH. (A) **1**, (B) **14**, (C) **18**, (D) **22**, (E) **27**, (F) **35**, (G) **37**, and (H) **45**. Interactions made by the inhibitors in the X-ray crystal structures of the complexes with *Mth* IMPDH (gray; and adjacent protomer in cyan) were calculated by Arpeggio [17], with ring and *pi* interactions shown in green, amide–amide in blue, hydrogen bonds in red, and polar interactions in orange. The electron density of the soaked complexes revealed that all the compounds bound into the NAD-binding pocket of IMPDH in a similar manner to compound **1.**

Compound **12**, in which a methyl group was added at position-1 of the cyclohexyl ring of **1** maintained inhibitory activity against IMPDH (IC_50_: 2.4 μM), but anti-tubercular activity was ablated (MIC_90_: 250 μM) suggesting that this modification affected compound permeation and/or metabolism in *M. tuberculosis*. In contrast, the addition of a methyl group at position-2 of the cyclohexyl ring moderately improved the inhibitory activity against IMPDH while retaining modest target-selective, whole-cell activity (compound **13**, MIC_90_: 31.2 μM; IC_50_: 0.084 μM). Replacement of the cyclohexyl by a cycloheptyl ring (compound **14**) reduced both enzyme and whole-cell activities by approximately one order of magnitude (MIC_90_: 31.2 μM, IC_50_: 0.83 μM) while maintaining target selectivity. The co-crystal structure of **14** with *Mth* IMPDH confirmed the binding of this compound to the NAD-binding pocket of IMPDH in a similar manner to compound **1**. Pharmacophore analysis of the binding (Fig. 4; Table 5) highlighted the pivotal role of interactions with **14** made at both hotspot regions identified (Fig. 2, Fig. 3).

**Table 4:**
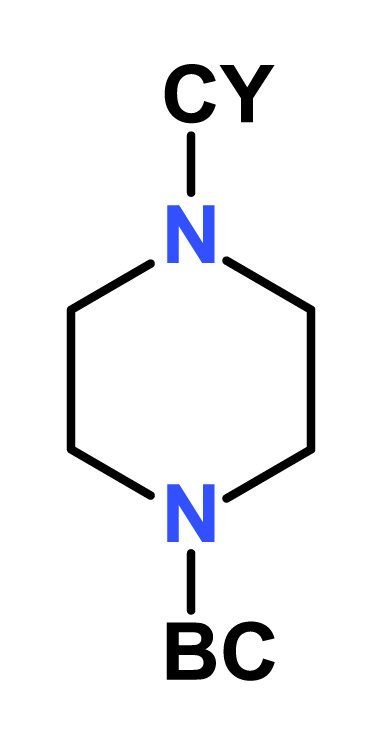
SAR profiling of compounds with increased distance of carbonyl group to the cyclohexyl ring or its analogs.

Cmpd No	CY	IMPDH IC_50_ (μM)	MIC_90_ (μM) against WT	MIC_90_ (μM) against SRMV2.6	Hyper-sensitivity to *guaB2* cKD
**1**		0.1 ± 0.01	2	250	∼32-fold
**22**		0.23 ± 0.02	31.2	>250	16-fold
**23**		3.3 ± 0.2	250	>250	ND^a^
**24**		5.96 ± 0.2	125	>250	ND
**25**		4.0 ± 0.2	125	>250	ND
**26**		2.6 ± 0.2	250	>250	ND
**27**		1.98 ± 0.1	250	>250	ND
**28**		11.48 ± 0.3	250	>250	ND
**29**		0.68 ± 0.05	25.0	>100	ND
**30**		5.3 ± 0.2	250	>250	ND
**31**		0.15 ± 0.03	15.6	31.2	ND
**32**		0.08 ± 0.005	62.5	125	ND
**33**		15.0 ± 0.5	250	>250	ND
**34**		0.16 ± 0.1	25	50	ND
**35**		5.6 ± 0.2	12.5	>100	ND
**36**		0.042 ± 0.002	31.2	62.5	ND
**37**		0.9 ± 0.06	125	>250	ND
**38**		1.6 ± 0.2	100	100	ND
**39**		0.6 ± 0.03	25	25	ND
**40**		3.4 ± 0.1	250	>250	ND
**41**		0.08 ± 0.005	6.3	>100	4-fold
**42**		1.1 ± 0.1	50	>100	ND
**43**		>100	>100	>100	ND
**44**		0.6 ± 0.02	12.5	>100	8–16-fold
**45**		0.12 ± 0.02	12.5	>100	8–16-fold
**46**		0.35 ± 0.03	3.1	>100	8-fold
**47**		0.035 ± 0.002	3.1	>100	8-fold
**48**		0.27 ± 0.03	3.9	15.6	16–32-fold
**49**		0.8 ± 0.05	100	100	ND

a ND, not determined.

**Table 5:** **Data collection and refinement statistics**. Statistics for the highest-resolution shell are shown in parentheses.

	compound 14	compound 18	compound 22	compound 27	compound 35	compound 37	compound 45
PDB Code	6D4Q	6D4R	6D4V	6D4U	6D4W	6D4S	6D4T
Beamline	I24	I24	I24	I24	I24	I24	I24
Wavelength (Å)	0.97	0.97	0.97	0.97	0.97	0.97	0.97
Resolution range (Å)	61.64–1.71 (1.77–1.71)	42.33–1.34 (1.39–1.34)	63.5–2.02 (2.09–2.02)	61.21–1.7 (1.76–1.7)	35.93–1.8 (1.86–1.8)	63.22–1.63 (1.69–1.63)	61.18–1.54 (1.60–1.54)
Space group	I 4	I 4	I 4	I 4	I 4	I 4	I 4
Unit cell	89.6 89.6 85.0 90 90 90	88.7 88.7 84.7 90 90 90	89.8 89.8 84.9 90 90 90	88.7 88.7 84.6 90 90 90	88.8 88.8 84.6 90 90 90	89.4 89.4 85.3 90 90 90	88.7 88.7 84.5 90 90 90
Total reflections	72178 (7214)	145626(14577)	44053 (4407)	71796 (7189)	56465 (5608)	83302 (8319)	96068 (9539)
Unique reflections	36236 (3621)	73343 (7332)	22169 (2223)	36025 (3612)	29914 (2956)	41811 (4173)	48275 (4799)
Multiplicity	2.0 (2.0)	2.0 (2.0)	2.0 (2.0)	2.0 (2.0)	1.9 (1.9)	2.0 (2.0)	2.0 (2.0)
Completeness (%)	100.0 (100.0)	99.9 (99.9)	100.0 (100.0)	100.0 (99.9)	98.5 (99.1)	100.0 (99.9)	100.0 (100.0)
Mean I/sigma(I)	13.6 (1.8)	12.9 (1.4)	10.6 (2.3)	5.4 (1.7)	12.5 (2.6)	16.2 (1.3)	11.9 (2.0)
Wilson B-factor	24.16	17.45	29.05	16.46	21.84	22.71	20.55
R-merge	0.047 (0.530)	0.037 (0.656)	0.105 (0.512)	0.082 (0.536)	0.057 (0.429)	0.034 (0.684)	0.040 (0.414)
R-meas	0.066	0.052	0.149	0.116	0.080	0.048	0.057
CC1/2	0.998 (0.34)	0.998 (0.487)	0.979 (0.23)	0.987 (0.283)	0.978 (0.57)	0.999 (0.51)	0.997 (0.472)
R-work	0.16 (0.28)	0.17 (0.29)	0.17 (0.28)	0.17 (0.25)	0.17 (0.24)	0.17 (0.27)	0.17 (0.26)
R-free	0.19 (0.32)	0.19 (0.33)	0.20 (0.34)	0.20 (0.27)	0.20 (0.27)	0.19 (0.31)	0.19 (0.30)
Number of non-hydrogen atoms	2650	2724	2554	2655	2600	2647	2670
macromolecules	2330	2335	2285	2280	2316	2319	2317
ligands	51	53	51	55	52	53	55
water	269	336	218	320	232	275	298
Protein residues	331	332	331	330	331	331	331
RMS(bonds)	0.008	0.007	0.009	0.009	0.009	0.011	0.008
RMS(angles)	1.15	1.27	1.13	1.15	1.18	1.19	1.20
Ramachandran favored (%)	98	98	98	98	98	98	98
Ramachandran outliers (%)	0	0	0	0	0	0	0
Clashscore	6.28	6.69	4.09	4.50	3.39	2.74	3.16
Average B-factor	26.90	23.20	29.80	20.20	25.60	27.90	25.70
macromolecules	25.60	21.80	29.00	18.50	24.40	26.70	24.10
ligands	24.30	21.00	27.20	20.30	26.30	26.50	24.80
solvent	38.60	33.60	39.30	32.60	36.90	38.10	37.70

##### 2.3.3.2 IIIb: aromatic derivatives

Substitution of the cyclohexyl by a phenyl ring (compound **15**) resulted in loss of both biochemical and whole-cell activities (IC_50_: 8.1 μM, MIC_90_: >100 μM). However, other derivatives containing substituted phenyl rings were more active than compound **15** (Table 3). The 3-cyano substitution (compound **16**) had no effect on the IC_50_ but improved whole-cell activity (MIC_90_: 25 μM) whereas the 4-cyano substitution (compound **17**) had a deleterious effect on both biochemical and whole-cell activity. However, substitution of the phenyl ring in compound **16** by a 3-nitrophenyl in compound **18** increased the inhibitory activity against both the enzyme and *M. tuberculosis* (IC_50_: 1.1 μM, MIC_90_: 12.5 μM). Electron density of the soaked complex with *Mth* IMPDH revealed that compound **18** exhibits extensive pi interactions with both IMP and A285 (A269 in the *Mth* structure) in the first hotspot region. The tail of **18** extended up into the second hotspot region where it made strong pi interactions with the Y487′ (Y471′ in the *Mth* structure) side chain from the adjacent molecule in the tetramer (Fig. 2, Fig. 3, Fig. 4; Table 5). The addition of a second nitro group at the meta position in the phenyl ring (compound **19**) had no further effect on either IC_50_ or MIC_90_ (3,5-dinitrophenyl, IC_50_: 1.4 μM, MIC_90_: 12.5 μM). Interestingly, the 3,4,5-trimethoxyphenyl derivative, compound **20**, displayed reasonably good whole-cell activity (MIC_90_: 15.6 μM) despite having a poor IC_50_ (12 μM) suggesting that the modifications are important for compound uptake.

**Fig. 5:**
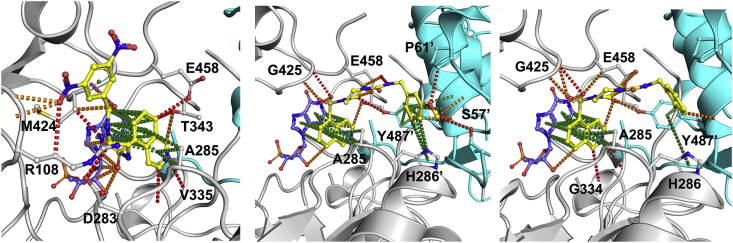
Docking analyses of compounds **21** (A), **47** (B), and **48** (C) bound to *Mth* IMPDH. Interactions made by compounds with *Mth* IMPDH (gray; and adjacent protomer in cyan) were calculated by Arpeggio [17], with ring and *pi* interactions shown in green, amide–amide in blue, hydrogen bonds in red, and polar interactions in orange.e.)

Based on the inhibitory characteristics of compounds **18**/**19** and **2**, we synthesized compound **21**, the ring-opened derivative of compound **19**. Compound **21** (IC_50_: 0.3 μM, MIC_90_: 15.6 μM) was highly active against the enzyme, and was 4-fold more active against the compound **1**-resistant mutant strain, SRMV2.6, than wild-type *M. tuberculosis* (MIC_90_: 3.9 μM *vs*. 15.6 μM). This compound also showed a 4-fold increase in potency against the *guaB2* cKD mutant upon *guaB2* silencing, thus confirming its target selectivity for IMPDH in *M. tuberculosis* and ability to bind at the active site of a mutant form of the enzyme in which the Tyr487 residue is substituted by Cys. Docking analysis supported the notion that compound **21** may exhibit a distinct mode of binding at the active site being independent of interaction with Y487’ (Fig. 5).

#### 2.3.4 Set IV: increasing the distance between the carbonyl group and the cyclohexyl ring or its analogues

In this set of compounds, the effect of (i) increasing the spacing between carbonyl group and the cyclohexyl ring or its analogues and (ii) change of one nitrogen of piperazine to carbon on the activity profile was investigated (Table 4).

##### 2.3.4.1 IVa: phenylacetic acid derivatives: methylene insertion

Insertion of a methylene between the carbonyl group and the cyclohexyl ring transformed the compounds into phenylacetic acid derivatives (compounds **22**–**30**; Table 4). Most of the compounds in this series showed reasonable activity against IMPDH, with **22** (cyclohexylacetic acid) and **29** (4-fluorophenylacetic acid) being the most potent (IC_50_: 0.23 μM and 0.68 μM, respectively). Although compound **22** and **29** were reasonably active against *M. tuberculosis* and **22** retained target selectivity in whole cells, as evidenced by the hypersensitivity of the *guaB2* cKD mutant to this compound, the MIC_90_ values of the other phenylacetic acid analogues were unfavorable. Electron density of the soaked complexes of **22**, **27**, and **29** with *Mth* IMPDH revealed that these molecules bound to the NAD binding pocket and made strong pi interactions with IMP and A285, in addition to a network of polar and hydrogen bonds to surrounding residues including T343, G4225 and E458 (Fig. 3, Fig. 4). Compounds **27** and **29** were also able to make interactions with adjacent protomer through the side chain of P61′ and Y487′.

##### 2.3.4.2 IVb: phenylurea derivatives: NH group insertion

A series of analogues containing mono or di substitutions of the phenyl ring were synthesized (compounds **31**–**45**, Table 4). These compounds were similar to the phenylacetic acid derivatives, but with a nitrogen in place of carbon. Some of the compounds showed good inhibitory activity against the enzyme but limited whole-cell activity (Table 4). When the phenyl ring was substituted at position 3, the resulting compounds generally displayed reduced whole-cell activity while maintaining IC_50_ values comparable to that of compound **1** (Table 4). Similarly, when the phenyl ring was substituted at other positions, the resulting compounds **33** (4-chloro phenylurea, MIC_90_: 250 μM, IC_50_: 15 μM); **39** (2-nitro, 4-methyl phenylurea; MIC_90_: 25 μM, IC_50_: 0.6 μM); and **40** (2-chloro, 6-methyl phenylurea, MIC_90_: 250 μM, IC_50_: 3.4 μM) were markedly less active against *M. tuberculosis*. Despite displaying limited whole-cell activity, the electron density of the soaked complex suggested that compound **37** was able to bind to *Mth* IMPDH in a mode similar to **1** (Fig. 4; Table 5). An additional chlorine atom at position-4 resulted in substantial loss of both biochemical and anti-mycobacterial activities (compounds **34** and **33)**. Thus, while substituting a carboxamide by a urea moiety improved the inhibitory activities of the 3-substituted phenyl derivatives against the enzyme, this did not translate into improved whole-cell activity, again pointing to an impact on compound permeation and/or metabolism in *M. tuberculosis*.

Next, based on the inhibitory characteristics of compound **20**, the methylenedioxyphenyl ring was selected for further evaluation (compound **41**–**45**, Table 4). In contrast to the other phenylureas, the 3,4 methylenedioxyphenyl urea derivative, **41**, showed promising whole-cell activity (MIC_90_: 6.3 μM), resistance to **SRMV2.6**, and a 4-fold increase in potency against the *guaB2* cKD mutant coupled with potent activity against the enzyme (IC_50_: 0.08 μM). While compound **42**, the thiophenylurea analogue of **41,** retained comparable activity against the enzyme (IC_50_: 1.1 μM), the replacement of oxygen by sulfur resulted in an 8-fold reduction in whole-cell activity. Replacement of the piperazine ring in **41** by the seven-membered homopiperazine ring in **44** resulted in an 8-fold reduction in biochemical activity and a 2-fold reduction in whole-cell activity. Interestingly, the phenyulrea compound **45**, in which the piperazine ring in **41** was replaced by 3-methylpiperazine, had a negligible effect on both biochemical and whole-cell activity. This is in contrast to the dramatic loss of activity observed when the same piperazine ring substitution was made in compound **1** to produce compound **3**, as described above. Moreover, both compounds **44** and **45** showed increased potency against the *guaB2* cKD mutant confirming that these compounds retained target selectivity for IMPDH in whole *M. tuberculosis* cells. Structural analysis confirmed tight binding of compound **45** to *Mth* IMPDH, similar to compound **1** (Fig. 2, Fig. 3, Fig. 4; Table 5).

##### 2.3.4.3 IVc: benzylurea derivatives: NHCH_2_ group insertion

Finally, to further increase the spacing between the carbonyl group and the cyclohexyl ring, we synthesized a series of benzylurea derivatives (**46**–**49**, Table 4). Compound **46** (IC_50_: 0.35 μM, MIC_90_: 3.1 μM), exhibited biochemical and whole-cell activities comparable to **1** and also retained target selectivity in *M. tuberculosis*. The 3-methoxybenzylurea derivative, compound **47**, was the most potent IMPDH inhibitor analysed in this study (IC_50_: 0.035 μM), demonstrating ∼3-fold greater potency than **1**, while maintaining whole-cell activity (MIC_90_: 3.1 μM) as well as target selectivity in *M. tuberculosis*. However, compound **47** was not active against SMRV2.6, a finding consistent with the docking analysis, which predicted a strong interaction between **47** and the Y487′ residue in the adjacent promoter (Fig. 5). Compound **48** (2-methyl benzylurea) also exhibited good anti-mycobacterial (MIC_90_: 3.9 μM) and biochemical (IC_50_: 0.27 μM) activity, while maintaining target selectivity in *M. tuberculosis*. This compound was also reasonably active against the resistant mutant, SRMV2.6 (MIC_90_: 15.6 μM) even though docking analysis suggested an interaction with the Y487’ residue (Fig. 5). We also evaluated the benzylurethane, compound **49**, which differs from the benzylurea compound **46** by only one atom. While retaining activity against the enzyme (IC_50_: 0.8 μM), this substitution abolished whole-cell activity (MIC_90_: 100 μM) (Table 5), underscoring the importance of the nitrogen atom in the urea moiety for compound engagement of the IMPDH target in *M. tuberculosis*.

### 2.4 X-ray crystallography

All compounds were evaluated for structural analysis and co-crystal structures were obtained for seven of these, namely compounds **14**, **18**, **22**, **27**, **35**, **37** and **45**. Computational analysis of the *Mth* IMPDH (GuaB2) structure revealed two main hotspot regions, both of which were located within the NAD binding pocket (Fig. 2). One of these was located near the bound IMP, where compounds could make apolar interactions to both the IMP and surrounding residues. The other was located close to the entrance of the active site, where apolar interactions, including to the neighbouring subunit, could be taken advantage of.

Pharmacophore analysis of the binding of the IMPDH inhibitors (including 5J5R [5], 5K4X and 5K4Z [8]) (Fig. 3) highlighted the pivotal role of interactions they made at both hotspot regions identified. In the first hotspot region, all of the analogues made extensive pi interactions with both IMP and A285 (A269 in the *Mth* structure). They could also make weak hydrogen bonds to the side chain of T343 (T327 in the *Mth* structure) and the main chain atoms of G334, V335, G336 and G425 (G318, V319, G320 and G409 in the *Mth* structure). The tails of the enzyme inhibitors extended up into the second hotspot region, where they all made strong pi interactions with the Y487′ (Y471′ in the *Mth* structure) side chain from the adjacent molecule in the tetramer.

The compounds **14**, **18**, **22**, **27**, **35**, **37** and **45** were also able to make additional interactions to P61′ side chain from the adjacent protomer (Fig. 4). In summary, it was observed that the compounds establish strong interactions to the *Mth* IMPDH, suggesting that factors such as compound permeation, metabolism and/or efflux could be the reason for their moderate to weak anti-tubercular activities.

In the case of compounds **21**, **47**, and **48**, our attempts to obtain co-crystals with *Mth* IMPDH were unsuccessful despite extensive efforts. As a result, docking analyses were performed instead for these compounds (Fig. 5). These analyses revealed that binding of compound **21** to the IMPDH is independent of interaction with Y487’.

## 3 Conclusions

In this paper, we describe a SAR investigation and optimisation around a previously reported inhibitor of *M. tuberculosis* IMPDH. The availability of a conditional knockdown mutant of *M. tuberculosis* in the IMPDH-encoding gene provided a key tool for assessing the target selectivity of the inhibitors in whole cells [5]. Furthermore, the X-ray crystal structure of the enzyme-bound inhibitor, which was solved during the course of this study, provided a useful comparator for the other enzyme-bound inhibitor structures reported here [5]. The present study yielded a number of reasonably potent compounds with on-target activity against this enzyme in *M. tuberculosis.* The results confirmed that the piperazine and isoquinoline rings of compound **1** are essential for target-selective whole-cell activity as any changes in these moieties resulted in the loss of activity against *M. tuberculosis*. While promising IMPDH enzyme inhibitors were identified among the carboxamide derivatives, these showed poor whole-cell activity. The complex, lipid-rich cell envelope of *M. tuberculosis* is thought to act as a significant permeability barrier for small molecules [18]. Thus, the LogP value of a small molecule, as a measure of its lipophilicity, can be a useful physicochemical property for interrogating MIC data. However, no direct relationship was found between LogP and/or predicted Caco-2 permeability (a measure of absorption of oral drugs), and whole-cell activity of the compounds against *M. tuberculosis* (Table S2). The best compounds in terms of whole-cell activity were benzylureas, whereas other urea derivatives exhibited better enzyme inhibition. Notably, the 3-methoxybenzylurea derivative, compound **47**, showed improved potency against the enzyme while maintaining on-target, whole-cell activity. Importantly, this study yielded a potent IMPDH inhibitor, compound, **21,** which was active against IMPDH in both wild type *M. tuberculosis* as well as the SRMV2.6 mutant, suggesting that, unlike compound **1** [5], its mode of binding at the active site is independent of interactions with the Tyr487 residue in the adjacent protomer. The distinct mode of binding of this compound, as reinforced by docking analysis, suggests the potential to develop more potent inhibitors that can bypass the mechanism of resistance to compound **1**. In summary, the results of this study provide a promising platform for further efforts to develop leads against *M. tuberculosis* IMPDH. However, they also serve as a timely reminder of the significant challenges in developing potent inhibitors of an essential enzyme that have the properties required to penetrate the cell envelope, evade metabolic detoxification [19], and engage their target in *M. tuberculosis*.

## 4 Methods

### 4.1 Design strategy and synthetic chemistry

The synthetic routes to the analogues of compound **1** (Table S1) are illustrated in Scheme 1, Scheme 2. Isoquinoline-5-sulfonyl chlorides were reacted with the corresponding amines (yield: 80–95%) using triethylamine as base in ethyl-acetate (method, i). We applied three kinds of protecting groups depending on the synthetic strategy and the availability of the starting materials. The N— acetyl group was removed by aqueous 2N hydrochloric acid (method, ii) with an yield of 50–80%, while the t.-Boc protecting group was removed by trifluoroacetic acid in dichloromethane under mild, anhydrous conditions with high yield (70–90%) (method, iii). The carboxybenzyl group was removed by catalytic hydrogenation under atmospheric pressure (method, iv) in ethanol giving the products in quantitative yield. The carboxamide derivatives were synthesized from different types of carbonyl compounds. Carbonyl chlorides were reacted either with the amine components in anhydrous pyridine (method, v) or under aqueous conditions, aq. NaHCO_3_/EtOAc (method, vi) yielding the products in moderate to excellent yield, whilst carboxylic acids were activated by DCC or EDCI in anhydrous pyridine (method, vii). Synthesis of urea derivatives required elevated tempemperature (100 °C) in dry pyridine for 12–18 h due to the inactivity of isocyanates/isothiocyanates (method, viii), with a product yield of 60–80%.

### 4.2 General procedures for the synthesis of compounds

#### 4.2.1 Synthesis of sulfonamide intermediates from sulfochlorides

##### 4.2.1.1 Method A

10 mM sulfochloride hydrochloride was suspended in 50 ml ethylacetate and 11 mM amine component was added with stirring. 2.8 ml triethylamine dissolved in 10 ml ethylacetate was dropped in with stirring and kept stirring overnight at ambient temperature. The reaction mixture was extracted three times with water, the organic phase was dried over sodium sulfate filtered, and then evaporated to dryness. For cases in which an oily product was obtained, the residue was treated with diisopropyl ether.

#### 4.2.2 Deprotection

##### 4.2.2.1 Method B

The acetyl protecting group was removed with 2 N hydrochloric acid while stirring for 3 h at 100 °C. After cooling, the pH of the reaction mixture was adjusted to alkaline with sodium bicarbonate and extracted with ethylacetate. The organic phase was dried on sodium sulfate, filtrated then ethylacetate saturated with hydrochloric acid gas was added dropwise with stirring. Crystalline hydrochloride salt was isolated by filtration, and washed carefully with ethylacetate and diethylether.

##### 4.2.2.2 Method C

The carbobenzyloxy group was removed by catalytic hydrogenation in ethanol solution in presence of palladium/charcoal catalyst. After filtration, the filtrate was evaporated to dryness then dissolved in ethylacetate and the product was isolated as hydrochloride salt.

##### 4.2.2.3 Method D

Boc-protected derivatives were deblocked with dichloromethane-trifluoroacetic acid (1:1). After stirring for a few hours, the solvent was removed, the residue was treated with 2M HCl in ethylacetate, and the product was isolated as a hydrochloride salt, as above.

#### 4.2.3 Synthesis of carboxamides

##### 4.2.3.1 Method E

0.5 mM amine dihydrochloride was dissolved in 2 ml dry pyridine and 0.6 mM acyl chloride was added. After several hours (reaction was monitored by TLC) the solvent was removed under vacuum and the residue was crystallized from ethanol:water (20:80). Crystals were collected by filtration and dried under vacuum. The product was purified by silica column chromatography if needed. (eluent, ethylacetate or chloroform: methanol, 10:1).

##### 4.2.3.2 Method F

0.5 mM amine dihydrochloride was dissolved/suspended in 20 ml saturated sodium bicarbonate solution. A solution of 1 mM acyl chloride in 20 ml ethylacetate was added and stirred overnight at ambient temperature. The organic phase was separated and extracted with brine then dried over sodium sulfate. After evaporation to dryness, and the crude product was purified by silica column chromatography, if needed, as above.

##### 4.2.3.3 Method G

The amine compound and 1.1 equivalent phenylacetic acid were dissolved in ethylacetate (in case of solubility problems 20–50% dimethylformamide was also added). 1.2 equivalent carbodiimide (dicyclohexylcarbodiimide or ethyl-dimethylaminopropyl carbodiimide) was added and stirred at ambient temperature overnight. Dicyclohexylurea was removed by filtration while ethyl-dimethylamino propylurea was removed by extraction with water and brine. The organic solvent was also extracted with sodium carbonate solution in both cases and the organic phase was dried and evaporated. The crude products were purified on silica column (eluent: ethylacetate or chloroform-methanol 10:1).

#### 4.2.4 Synthesis of ureas

##### 4.2.4.1 Method H

The amine component was dissolved in 5 ml dry pyridine and 1.1 equivalent isocyanate was added. The reaction mixture was stirred for 4 h at 100 °C. Solvent was removed in vacuum and the residue was dissolved in ethylacetate and extracted three times with 10% sodium dihydrogenphosphate (pH 6) solution. The organic phase was separated, dried on sodium sulfate, evaporated and the residue was chromatographed on a silica column using ethylacetate or chloroform: methanol (10:1) eluent. Pure fractions were combined and evaporated. The residue was treated with diisopropyl ether, and crystals were isolated by filtration.

#### 4.2.5 Synthesis of compound **19**

5-(1,4-piperazinyl-1-sulfonyl) isoquinoline was acylated with 2-nitrobenzoylchloride according to General Method F. The nitro function was reduced by iron powder in ethanol-water solution (2:1) containing 3% acetic acid at reflux temperature. After 2 h of stirring compound **19** was isolated and purified by column chromatography.

#### 4.2.6 NMR and LC-MS analyses

The ^1^H and ^13^C NMR spectra of the compounds were recorded on a Bruker Avance 300 NMR spectrometer operating at 7.05 Tesla magnetic field, controlled by TopSpin 1.3 software package, equipped with 5 mm ^1^H/^13^C dual inverse Z-grad probehead, and in DMSO‑*d*_6_ solution at 30 °C. The chemical shifts are referred to tetramethylsilane (δTMS = 0 ppm). The chemical structures of the compounds were proven based on two dimensional zqs-TOCSY [20], zqs-easy-ROESY [[20], [21], [22]], multiplicity-edited HSQC as well as HMBC spectra. The LC-MS analyses were performed with Waters liquid chromatograph - mass spectrometer (mass detector, Waters SQD; UV detector, Waters 996 DAD; separation module, Waters Alliance 2795; column, Waters XBridge C18 50 × 4.6 mm 3.5 μm; and the gradient used with solvent-1, water (Mili-Q) containing 0.1% formic acid (Riedel- DeHaen, extra-pure), and solvent-2, acetonitrile (Riedel- DeHaen). The flow-rate was 2 ml/min with 5 μg sample. The MS parameters were used as: Ionisation, ES+/ES-; source block temperature, 110 °C; desolvation temperature, 250 °C; desolvation gas, 500 L/h; cone gas, 80L/h; capillary voltage, 3000 V; cone voltage, 30 V; extractor voltage, 6 V; Rf lens Voltage, 0.1 V; Scan: 80–1000 *m*/*z* in 1 s; and Inter-scan delay, 0.1 s.

#### 4.2.7 Cyclohexyl[4-(isoquinoline-5-sulfonyl)piperazin-1-yl]methanone (**1**)

LCMS[MH+]: 388.1, MW calc: 387.162, Rt: 3.29 min.

^1^H NMR (300 MHz, DMSO‑*d*_6_) *δ*: 9.50 (s, 1H); 8.70 (d, *J* = 6.2 Hz, 1H); 8.52 (dd, *J* = 8.2 and 1.1 Hz, 1H); 8.44 (d, *J* = 6.2 Hz, 1H); 8.36 (dd, *J* = 7.4 and 1.1 Hz, 1H); 7.88 (dd, *J* = 8.2 and 7.4 Hz, 1H); 3.50 (tm, *J* = 4.7 Hz, 4H); 3.07 (tm, *J* = 4.7 Hz, 4H); 2.46 (m, 1H); 1.61 (dm, *J* ∼ 9.0 Hz, 2H); 1.45–1.58 (ovl. m, 3H); 1.16–1.27 (ovl. m, 4H); 1.11 (m, 1H).

^13^C NMR (75 MHz, DMSO‑*d*_6_) *δ*: 173.6; 153.7; 145.1; 134.6; 134.4; 131.2; 131.1; 128.9; 126.7; 117.2; 45.9; 45.6; 44.4; 40.5; 39.0; 29.1; 25.6; 25.2.

#### 4.2.8 N-{2-[(isoquinoline-5-sulfonyl)amino]ethyl}cyclohexanecarboxamide (**2**)

LCMS[MH+]: 362.1, MW calc: 361.146, Rt: 2.68 min.

^1^H NMR (300 MHz, DMSO‑*d*_6_) *δ*: 9.47 (s, 1H); 8.70 (d, *J* = 6.2 Hz, 1H); 8.44 (dd, *J* = 8.0 and 1.2 Hz, 1H); 8.41 (d, *J* = 6.2 Hz, 1H); 8.32 (dd, *J* = 7.5 and 1.2 Hz, 1H); 8.11 (br. s, 1H); 7.83 (dd, *J* = 8.0 and 7.5 Hz, 1H); 7.55 (t, *J* = 5.5 Hz, 1H); 3.00 (td, *J* = 6.7 and 5.5 Hz, 2H); 2.79 (t, *J* = 6.7 Hz, 2H); 1.87 (tt, *J* ∼ 9.0 and 3.0 Hz, 1H); 1.45–1.70 (ovl. m, 5H); 1.05–1.25 (ovl. m, 5H).

^13^C NMR (75 MHz, DMSO‑*d*_6_) *δ*: 175.4; 153.5; 144.7; 134.7; 133.6; 132.6; 130.4; 128.8; 126.6; 117.3; 44.0; 42.0; 38.4; 29.1; 25.5; 25.3.

#### 4.2.9 Cyclohexyl[4-(isoquinoline-5-sulfonyl)-2-methylpiperazin-1-yl]methanone (**3**)

LCMS[MH+]: 402.3, MW calc: 401.177, Rt: 3.37 min.

^1^H NMR (300 MHz, DMSO‑*d*_6_) *δ*: 9.73 (s, 1H); 8.77 (d, *J* = 6.2 Hz, 1H); 8.63 (dd, *J* = 8.2 and 1.2 Hz, 1H); 8.57 (d, *J* = 6.2 Hz, 1H); 8.48 (dd, *J* = 7.3 and 1.2 Hz, 1H); 7.98 (dd, *J* = 8.2 and 7.3 Hz, 1H); 4.20 (ovl. m, 2H); 4.15 (m, 1H); 3.61 (m, 1H); 3.19 (m, 1H); 2.92 (m, 1H); 2.50 (m, 2H); 1.45-1-73 (ovl. m, 5H); 1.10–1.38 (ovl. m, 5H); 1.07 (d, *J* ∼ 5.5 Hz, 1.5H); 0.90 (d, *J* ∼ 5.5 Hz, 1.5H).

#### 4.2.10 N-[1-(isoquinoline-5-sulfonyl)piperidin-4-yl]cyclohexanecarboxamide (**4**)

LCMS[MH+]: 402.4, MW calc: 401.177, Rt: 3.14 min.

^1^H NMR (300 MHz, DMSO‑*d*_6_) *δ*: 9.50 (s, 1H); 8.70 (d, *J* = 6.1 Hz, 1H); 8.50 (dd, *J* = 8.0 and 1.1 Hz, 1H); 8.40 (d, *J* = 6.1 Hz, 1H); 8.36 (dd, *J* = 7.5 and 1.1 Hz, 1H); 7.87 (dd, *J* = 8.0 and 7.5 Hz, 1H); 7.57 (d, *J* = 7.7 Hz, 1H); 3.61 (ddd, *J* ∼ 12.5, 2.5 and 2.5 Hz, 2H); 3.57 (m, 1H); 2.78 (ddd, *J* ∼ 12.5, 10.5 and 2.5 Hz, 2H); 1.98 (tt, *J* ∼ 11.0 and 2.5 Hz, 1H); 1.71 (dm, *J* ∼ 12.0 Hz, 2H); 1.52–1.69 (ovl. m, 5H); 1.32 (ddd, *J* ∼ 12.0, 10.5, 10.0, and 2.5 Hz, 2H); 1.06–1.28 (ovl. m, 5H).

^13^C NMR (75 MHz, DMSO‑*d*_6_) *δ*: 174.6; 153.7; 145.0; 134.3; 134.0; 132.2; 131.0; 128.9; 126.7; 117.2; 44.3; 44.2; 44.0; 31.0; 29.3; 25.5; 25.4.

#### 4.2.11 N-[1-(cyclohexanecarbonyl)piperidin-4-yl]isoquinoline-5-sulfonamide (**5**)

LCMS[MH+]: 402.4, MW calc: 401.177, Rt: 3.07 min.

^1^H NMR (300 MHz, DMSO‑*d*_6_) *δ*: 9.48 (s, 1H); 8.70 (d, *J* = 6.1 Hz, 1H); 8.44 (dd, *J* = 8.0 and 1.1 Hz, 1H); 8.43 (d, *J* = 6.1 Hz, 1H); 8.38 (dd, *J* = 7.5 and 1.1 Hz, 1H); 8.21 (d, *J* = 8.0 Hz, 1H); 7.84 (dd, *J* = 8.0 and 7.5 Hz, 1H); 4.05 (dm, *J* ∼ 13.5 Hz, 1H); 3.69 (dm, *J* ∼ 13.5 Hz, 1H); 3.27 (m, 1H); 2.92 (ddm, *J* ∼ 13.5 and 11.0 Hz, 1H); 2.55 (ddm, *J* ∼ 13.5 and 11.0 Hz, 1H); 2.46 (m, 1H); 1.40–1.70 (ovl. m, 7H); 1.05–1.35 (ovl. m, 7H).

#### 4.2.12 Cyclohexyl[4-(3-methylisoquinoline-5-sulfonyl)piperazin-1-yl]methanone (**6**)

LCMS[MH+]: 402.4, MW calc: 401.177, Rt: 3.24 min.

^1^H NMR (300 MHz, DMSO‑*d*_6_) *δ*: 9.39 (s, 1H); 8.45 (dd, *J* = 8.0 and 1.1 Hz, 1H); 8.30 (dd, *J* = 7.5 and 1.1 Hz, 1H); 8.24 (s, 1H); 7.78 (dd, *J* = 8.0 and 7.5 Hz, 1H); 3.50 (tm, *J* = 4.7 Hz, 4H); 3.07 (tm, *J* = 4.7 Hz, 4H); 2.69 (s, 3H); 2.48 (m, 1H); 1.47–1.67 (ovl. m, 5H); 1.08–1.29 (ovl. m, 5H).

#### 4.2.13 Cyclohexyl[4-(naphthalene-1-sulfonyl)piperazin-1-yl]methanone (**7**)

LCMS[MH+]: 387.2, MW calc: 386.166, Rt: min, 4.23 min.

^1^H NMR (300 MHz, DMSO‑*d*_6_) *δ*: 8.66 (dd, *J* = 8.0 and 1.1 Hz, 1H); 8.30 (dd, *J* = 8.2 and 1.1 Hz, 1H); 8.14 (dm, *J* ∼ 7.5 Hz, 1H); 8.12 8 dm, *J* ∼ 8.0 Hz, 1H); 7.74 (dd, *J* = 8.2 and 8.0 Hz, 1H); 7.64–7.73 (ovl. m, 2H); 3.48 (tm, *J* = 4.7 Hz, 4H); 3.07 (tm, *J* = 4.7 Hz, 4H); 2.47 (m, 1H); 1.47–1.67 (ovl. m, 5H); 1.05–1.29 (ovl. m, 5H).

#### 4.2.14 Cyclohexyl[4-(naphthalene-2-sulfonyl)piperazin-1-yl]methanone (**8**)

LCMS[MH+]: 387.2, MW calc: 386.166, Rt: 4.24 min.

^1^H NMR (300 MHz, DMSO‑*d*_6_) *δ*: 8.44 (d, *J* ∼ 1.5 Hz, 1H); 8.21 (dm, *J* ∼ 8.2 Hz, 1H); 8.17 (d, *J* = 8.7 Hz, 1H); 8.08 (dm, *J* = 8.4 Hz, 1H); 7.67–7.77 (ovl. m, 3H); 3.54 (tm, *J* ∼ 4.7 Hz, 4H); 2.95 (tm, *J* ∼ 4.7 Hz, 4H); 2.46 (m, 1H); 1.45–1.65 (ovl. m, 5H); 1.00–1.25 (ovl. m, 5H).

#### 4.2.15 N-{2-[(isoquinoline-5-sulfonyl)amino]ethyl}cyclopropanecarboxamide (**9**)

LCMS[MH+]: 320.1, MW calc: 319.100, Rt: 1.93 min.

^1^H NMR (300 MHz, DMSO‑*d*_6_) *δ*: 9.48 (s, 1H); 8.69 (d, *J* = 6.2 Hz, 1H); 8.44 (dd, *J* = 8.0 and 1.2 Hz, 1H); 8.41 (d, *J* = 6.2 Hz, 1H); 8.33 (dd, *J* = 7.5 and 1.2 Hz, 1H); 8.16 (br. s, 1H); 7.97 (t, *J* = 5.5 Hz, 1H); 7.83 (dd, *J* = 8.0 and 7.5 Hz, 1H); 3.04 (td, *J* = 6.7 and 5.5 Hz, 2H); 2.81 (t, *J* = 6.7 Hz, 2H); 1.36 (m, 1H); 0.50–0.65 (m, 4H).

#### 4.2.16 N-{2-[(isoquinoline-5-sulfonyl)amino]ethyl}cyclobutanecarboxamide (**10**)

LCMS[MH+]: 334.2, MW calc: 333.115, Rt: 2.2 min.

^1^H NMR (300 MHz, DMSO‑*d*_6_) *δ*: 9.48 (s, 1H); 8.70 (d, *J* = 6.2 Hz, 1H); 8.44 (dd, *J* = 8.0 and 1.2 Hz, 1H); 8.40 (d, *J* = 6.2 Hz, 1H); 8.32 (dd, *J* = 7.5 and 1.2 Hz, 1H); 8.14 (t, *J* = 5.5 Hz, 1H); 7.83 (dd, *J* = 8.0 and 7.5 Hz, 1H); 7.54 (t, *J* = 5.5 Hz, 1H); 3.01 (td, *J* = 6.7 and 5.5 Hz, 2H); 2.81 (td, *J* = 6.7 and 5.5 Hz, 2H); 2.79 (ovl. m, 1H); 1.84–2.06 (ovl. m, 4H); 1.80 (m, 1H); 1.67 (m, 1H).

#### 4.2.17 1-[4-(isoquinoline-5-sulfonyl)piperazin-1-yl]-2,2-dimethylpropan-1-one (**11**)

LCMS[MH+]: 336.2, MW calc: 335.130, Rt: 2.43 min.

^1^H NMR (300 MHz, DMSO‑*d*_6_) *δ*: 9.48 (s, 1H); 8.70 (d, *J* = 6.2 Hz, 1H); 8.44 (dd, *J* = 8.0 and 1.1 Hz, 1H); 8.41 (d, *J* = 6.2 Hz, 1H); 8.33 (dd, *J* = 7.5 and 1.1 Hz, 1H); 8.14 (br. s, 1H); 7.84 (dd, *J* = 8.0 and 7.5 Hz, 1H); 7.35 (t, *J* = 5.5 Hz, 1H); 3.03 (td, *J* = 6.7 and 5.5 Hz, 2H); 2.80 (t, *J* = 6.7 Hz, 2H); 0.96 (s, 9H).

#### 4.2.18 [4-(isoquinoline-5-sulfonyl)piperazin-1-yl](1-methylcyclohexyl)methanone (**12**)

LCMS[MH+]: 402.2, MW calc: 401.177, Rt: 3.53 min.

^1^H NMR (300 MHz, DMSO‑*d*_6_) *δ*: 9.67 (s, 1H); 8.74 (d, *J* = 6.1 Hz, 1H); 8.61 (dd, *J* = 8.0 and 1.1 Hz, 1H); 8.57 (d, *J* = 6.1 Hz, 1H); 8.46 (dd, *J* = 7.5 and 1.1 Hz, 1H); 7.97 (dd, *J* = 8.0 and 7.5 Hz, 1H); 3.58 (tm, *J* = 4.7 Hz, 4H); 3.08 (tm, *J* = 4.7 Hz, 4H); 1.81 (m, 2H); 1.14–1.43 (ovl.m, 8H); 1.06 (s, 3H).

#### 4.2.19 [4-(isoquinoline-5-sulfonyl)piperazin-1-yl](2-methylcyclohexyl)methanone (**13**)

LCMS[MH+]: 402.2, MW calc: 401.177, Rt: 3.41 min.

^1^H NMR (300 MHz, DMSO‑*d*_6_) *δ*: 9.75 (s, 1H); 8.76 (d, *J* = 6.1 Hz, 1H); 8.67 (dd, *J* = 8.0 and 1.1 Hz, 1H); 8.65 (d, *J* = 6.1 Hz, 1H); 8.51 (dd, *J* = 7.5 and 1.1 Hz, 1H); 8.01 (dd, *J* = 8.0 and 7.5 Hz, 1H); 3.45–3.60 (ovl. m, 4H); 2.96–3.15 (ovl. m, 4H); 2.66 (m, 1H); 1.83 (m, 1H); 1.53–1.62 (ovl. m, 2H); 1.48 (m, 2H); 1.31 (m, 2H); 1.23 (m, 1H); 1.16 (m, 1H); 0.69 (d, *J* = 7.0 Hz, 3H).

#### 4.2.20 Cycloheptyl[4-(isoquinoline-5-sulfonyl)piperazin-1-yl]methanone (**14**)

LCMS[MH+]: 402.2, MW calc: 401.177, Rt: 3.42 min.

^1^H NMR (300 MHz, DMSO‑*d*_6_) *δ*: 9.74 (s, 1H); 8.75 (d, *J* = 6.1 Hz, 1H); 8.66 (dd, *J* = 8.0 and 1.1 Hz, 1H); 8.65 (d, *J* = 6.1 Hz, 1H); 8.50 (dd, *J* = 7.5 and 1.1 Hz, 1H); 8.00 (dd, *J* = 8.0 and 7.5 Hz, 1H); 3.50 (tm, *J* = 4.7 Hz, 4H); 3.08 (tm, *J* = 4.7 Hz, 4H); 2.63 (m, 1H); 1.52–1.66 (ovl. m, 5H); 1.27–1.52 (ovl. m, 7H).

#### 4.2.21 [4-(isoquinoline-5-sulfonyl)piperazin-1-yl](phenyl)methanone (**15**)

LCMS[MH+]: 382.43, MW calc: 381.46, Rt: 3.30 min.

^1^H NMR (300 MHz, DMSO‑*d*_6_) *δ*: 9.47 (s, 1H); 8.70 (d, *J = *6.2 Hz, 1H); 8.44 (dd, *J = *8.0 and 1.2 Hz, 1H); 8.41 (d, *J = *6.2 Hz, 1H); 8.32 (dd, *J = *7.5 and 1.2 Hz, 1H); 8.11 (br. s, 1H); 7.83 (dd, *J = *8.0 and 7.5 Hz, 1H); 7.55 (t, *J = *5.5 Hz, 1H); 3.00 (td, *J = *6.7 and 5.5 Hz, 2H); 2.79 (t, *J = *6.7 Hz, 2H); 1.87 (tt, *J* ∼9.0 and 3.0 Hz, 1H); 1.45–1.70 (ovl. m, 5H); 1.05–1.25 (ovl. m, 5H).

#### 4.2.22 3-[4-(isoquinoline-5-sulfonyl)piperazine-1-carbonyl]benzonitrile (**16**)

LCMS[MH+]:397.2 MW calc: 396.12 Rt: 2.68 min.

^1^H NMR (300 MHz, DMSO‑*d*_6_) *δ*: 9.50 (s, 1H); 8.69 (d, *J* = 6.1 Hz, 1H); 8.51 (dd, *J* = 8.0 and 1.1 Hz, 1H); 8.43 (d, *J* = 6.1 Hz, 1H); 8.36 (dd, *J* = 7.5 and 1.1 Hz, 1H); 7.88 (dd, *J* = 8.0 and 7.5 Hz, 1H); 7.04 (ddd, *J* = 8.1, 7.2 and 1.4 Hz, 1H); 6.90 (dd, *J* = 7.8 and 1.4 Hz, 1H); 6.63 (d, *J* = 8.1 Hz, 1H); 6.49 (dd, *J* = 7.8 and 7.2 Hz, 1H); 5.07 (s, 2H); 3.47 (tm, *J* = 4.7 Hz, 4H); 3.17 (tm, *J* = 4.7 Hz, 4H).

#### 4.2.23 4-[4-(isoquinoline-5-sulfonyl)piperazine-1-carbonyl]benzonitrile (**17**)

LCMS[MH+]: 407.0, MW calc: 406.110, Rt: 3.17 min.

^1^H NMR (300 MHz, DMSO‑*d*_6_) *δ*: 9.51 (s, 1H); 8.69 (d, *J* = 6.2 Hz, 1H); 8.52 (dd, *J* = 8.2 and 1.1 Hz, 1H); 8.42 (d, *J* = 6.2 Hz, 1H); 8.36 (dd, *J* = 7.4 and 1.1 Hz, 1H); 7.89 (dd, *J* = 8.2 and 7.4 Hz, 1H); 7.87 (dm, *J* = 8.2 Hz, 2H); 7.52 (dm, *J* = 8.2 Hz, 2H); 3.67 (m, 2H); 3.32 (m, 2H); 3.23 (m, 2H); 3.15 (m, 2H).

#### 4.2.24 [4-(isoquinoline-5-sulfonyl)piperazin-1-yl](3-nitrophenyl)methanone (**18**)

LCMS[MH+]: 427.3, MW calc: 426.100, Rt: 3.09 min.

^1^H NMR (300 MHz, DMSO‑*d*_6_) *δ*: 9.52 (s, 1H); 8.70 (d, *J* = 6.2 Hz, 1H); 8.53 (dd, *J* = 8.2 and 1.1 Hz, 1H); 8.44 (d, *J* = 6.2 Hz, 1H); 8.38 (dd, *J* = 7.4 and 1.1 Hz, 1H); 8.26 (ddd, *J* = 8.1, 1.6 and 1.3 Hz, 1H); 8.17 (dd, *J* = 1.6 and 1.3 Hz, 1H); 7.90 (dd, *J* = 8.2 and 7.4 Hz, 1H); 7.78 (ddd, *J* = 7.6, 1.3 and 1.3 Hz, 1H); 7.69 (dd, *J* = 8.1 and 7.6 Hz, 1H); 3.68 (m, 2H); 3.33 (m, 2H); 3.25 (m, 2H); 3.18 (m, 2H).

#### 4.2.25 [4-(isoquinoline-5-sulfonyl)piperazin-1-yl](3,5- dinitrophenyl)methanone (**19**)

LCMS[MH+]: 473.3, MW calc: 471.085, Rt: 3.35 min.

^1^H NMR (300 MHz, DMSO‑*d*_6_) *δ*: 9.52 (s, 1H); 8.82 (t, *J* = 2.1 Hz, 1H); 8.70 (d, *J* = 6.2 Hz, 1H); 8.59 (d, *J* = 2.1 Hz, 2H); 8.54 (dd, *J* = 8.2 and 1.1 Hz, 1H); 8.44 (d, *J* = 6.2 Hz, 1H); 8.38 (dd, *J* = 7.4 and 1.1 Hz, 1H); 7.90 (dd, *J* = 8.2 and 7.4 Hz, 1H); 3.70 (m, 2H); 3.35 (m, 2H); 3.27 (m, 2H); 3.20 (m, 2H).

#### 4.2.26 [4-(isoquinoline-5-sulfonyl)piperazin-1-yl](3,4,5-trimethoxyphenyl)methanone (**20**)

LCMS[MH+]: 468.3, MW calc: 467.093, Rt: 3.63 min.

^1^H NMR (300 MHz, DMSO‑*d*_6_) *δ*: 9.51 (s, 1H); 8.70 (d, *J* = 6.2 Hz, 1H); 8.53 (dd, *J* = 8.2 and 1.1 Hz, 1H); 8.43 (d, *J* = 6.2 Hz, 1H); 8.37 (dd, *J* = 7.4 and 1.1 Hz, 1H); 7.89 (dd, *J* = 8.2 and 7.4 Hz, 1H); 7.76 (ddd, *J* = 7.5 and 1.5 Hz, 1H); 7.74 (ddd, *J* = 8.5, 5.5 and 1.5 Hz, 1H); 7.54 (dd, *J* = 11.0 and 8.5 Hz, 1H); 3.63 (m, 2H); 3.39 (m, 2H); 3.19 (m, 4H).

#### 4.2.27 N-{2-[(isoquinoline-5-sulfonyl)amino]ethyl}-3,5-dinitrobenzamide (**21**)

LCMS[MH+]: 446.0, MW calc: 445.069, Rt: 2.95 min.

^1^H NMR (300 MHz, DMSO‑*d*_6_) *δ*: 9.28 (s, 1H); 9.04 (t, *J* = 5.5 Hz, 1H); 8.94 (t, *J* = 2.0 Hz, 1H); 8.79 (d, *J* = 2.0 Hz, 2H); 8.59 (d, *J* = 6.2 Hz, 1H); 8.37 (d, *J* = 6.2 Hz, 1H); 8.33 (br, 1H); 8.32 (dd, *J* = 7.5 and 1.1 Hz, 1H); 8.29 (dd, *J* = 8.2 and 1.1 Hz, 1H); 7.77 (dd, *J* = 8.2 and 7.4 Hz, 1H); 3.30 (td, *J* = 6.5 and 5.5 Hz, 2H); 3.08 (t, *J* = 6.5 Hz, 2H).

^13^C NMR (75 MHz, DMSO‑*d*_6_) *δ*: 162.0; 153.3; 148.2; 144.6; 136.5; 134.8; 133.4; 132.6; 130.3; 128.7; 127.3; 126.5; 120.9; 117.3; 41.3; 39.7.

#### 4.2.28 2-Cyclohexyl-1-(4-(isoquinolin-5-ylsulfonyl)piperazin-1-yl)ethan-1-one (**22**)

LCMS[MH+]: 402.2, MW calc: 401.177, Rt: 3.45 min.

^1^H NMR (300 MHz, DMSO‑*d*_6_) *δ*: 9.73 (s, 1H); 8.75 (d, *J* = 6.1 Hz, 1H); 8.66 (dd, *J* = 8.0 and 1.1 Hz, 1H); 8.64 (d, *J* = 6.1 Hz, 1H); 8.50 (dd, *J* = 7.5 and 1.1 Hz, 1H); 8.00 (dd, *J* = 8.0 and 7.5 Hz, 1H); 3.49 (tm, *J* = 4.7 Hz, 4H); 3.08 (m, 4H); 2.09 (d, *J* = 6.4 Hz, 2H); 1.48–1.64 (ovl. m, 6H); 1.04–1.18 (ovl. m, 3H); 0.75–0.90 (ovl. m, 2H).

#### 4.2.29 2-(1,4-dioxaspiro[4.5]decan-8-yl)-1-[4-(isoquinoline-5-sulfonyl)piperazin-1-yl]ethan-1-one (**23**)

LCMS[MH+]: 461.2, MW calc: 460.178, Rt: 1.99 min.

^1^H NMR (300 MHz, DMSO‑*d*_6_) *δ*: 9.83 (br. s, 1H); 9.64 (s, 1H); 8.74 (d, *J* = 6.1 Hz, 1H); 8.61 (dd, *J* = 8.0 and 1.1 Hz, H); 8.54 (d, *J* = 6.1 Hz, 1H); 8.46 (dd, *J* = 7.5 and 1.1 Hz, 1H); 7.97 (dd, *J* = 8.0 and 7.5 Hz, 1H); 4.28 (s, 2H); 3.90 (s, 4H); 3.57 (m, 2H); 3.41 (m, 2H); 3.35 (m, 2H); 3.18 (m, 2H); 3.11 (m, 2H); 3.06 (m, 2H); 1.96 (m, 2H); 1.84 (m, 2H).

#### 4.2.30 3-{2-[4-(isoquinoline-5-sulfonyl)piperazin-1-yl]-2-oxoethyl}benzonitrile (**24**)

LCMS[MH+]: 421.2, MW calc: 420.126, Rt:2.90 min.

^1^H NMR (300 MHz, DMSO‑*d*_6_) *δ*: 9.66 (s, 1H); 8.73 (d, *J* = 6.1 Hz, 1H); 8.62 (dd, *J* = 8.0 and 1.1 Hz, 1H); 8.57 (d, *J* = 6.1 Hz, 1H); 8.45 (dd, *J* = 7.5 and 1.1 Hz, 1H); 7.97 (dd, *J* = 8.0 and 7.5 Hz, 1H); 7.62 (dm, *J* = 6.6 Hz, 1H); 7.55 (dd, *J* = 1.5 and 1.5 Hz, 1H); 7.38–7.47 (ovl. m, 2H); 3.72 (s, 2H); 3.56 (tm, *J* = 4.7 Hz, 2H); 3.52 (tm, *J* = 4.7 Hz, 2H); 3.08 (tm, *J* = 4.7 Hz, 4H).

#### 4.2.31 2-(3-hydroxyphenyl)-1-[4-(isoquinoline-5-sulfonyl)piperazin-1-yl]ethan-1-one (**25**)

LCMS[MH+]: 412.2, MW calc: 411.125, Rt: 2.60 min.

^1^H NMR (300 MHz, DMSO‑*d*_6_) *δ*: 9.50 (s, 1H); 9.21 (s, 1H); 8.68 (d, *J* = 6.1 Hz, 1H); 8.52 (dd, *J* = 8.0 and 1.1 Hz, 1H); 8.40 (d, *J* = 6.1 Hz, 1H); 8.33 (dd, *J* = 7.5 and 1.1 Hz, 1H); 7.87 (dd, *J* = 8.0 and 7.5 Hz, 1H); 6.94 (ddm, *J* = 8.8 and 7.2 Hz, 1H); 6.47–6.55 (ovl. m, 3H); 3.53 (s, 2H); 3.49 (tm, *J* = 4.7 Hz, 4H); 3.04 (tm, *J* = 4.7 Hz, 2H); 2.94 (tm, *J* = 4.7 Hz, 2H).

#### 4.2.32 1-[4-(isoquinoline-5-sulfonyl) piperazin-1-yl]-2-(3-methoxyphenyl)ethan-1-one (**26**)

LCMS[MH+]: 426.2, MW calc: 425.141, Rt: 2.99 min.

^1^H NMR (300 MHz, DMSO‑*d*_6_) *δ*: 9.74 (s, 1H); 8.73 (d, *J* = 6.1 Hz, 1H); 8.66 (dd, *J* = 8.0 and 1.1 Hz, 1H); 8.61 (d, *J* = 6.1 Hz, 1H); 8.46 (dd, *J* = 7.5 and 1.1 Hz, 1H); 7.99 (dd, *J* = 8.0 and 7.5 Hz, 1H); 7.06 (dd, *J* = 7.9 and 7.9 Hz, 1H); 6.60–6.72 (ovl. m, 3H); 3.63 (s, 3H); 3.59 (s, 2H); 3.52 (tm, *J* = 4.7 Hz, 4H); 3.03 (tm, *J* = 4.7 Hz, 2H); 2.92 (tm, *J* = 4.7 Hz, 2H).

#### 4.2.33 1-[4-(isoquinoline-5-sulfonyl) piperazin-1-yl]-2-(2,4-dimethoxyphenyl)ethan-1-one (**27**)

LCMS[MH+]: 456.2, MW calc: 456.2, Rt: 3.08 min.

^1^H NMR (300 MHz, DMSO‑*d*_6_) *δ*: 9.68 (s, 1H); 8.74 (d, *J* = 6.1 Hz, 1H); 8.63 (dd, *J* = 8.0 and 1.1 Hz, 1H); 8.59 (d, *J* = 6.1 Hz, 1H); 8.45 (dd, *J* = 7.5 and 1.1 Hz, 1H); 7.97 (dd, *J* = 8.0 and 7.5 Hz, 1H); 6.87 (d, *J* = 8.3 Hz, 1H); 4.43 (d, *J* = 2.1 Hz, 1H); 6.34 (dd, *J* = 8.3 and 2.1 Hz, 1H); 3.70 (s, 3H); 3.59 (s, 3H); 3.52 (tm, *J* = 4.7 Hz, 4H); 3.42 (s, 2H); 3.03 (tm, *J* = 4.7 Hz, 4H).

#### 4.2.34 1-(4-(isoquinolin-5-ylsulfonyl)piperazin-1-yl)-2-(m-tolyl)ethan-1-one (**28**)

LCMS[MH+]: 410.2, MW calc: 409.146, Rt: 3.22 min.

^1^H NMR (300 MHz, DMSO‑*d*_6_) *δ*: 9.52 (s, 1H); 8.68 (d, *J* = 6.1 Hz, 1H); 8.53 (dd, *J* = 8.0 and 1.1 Hz, 1H); 8.40 (d, *J* = 6.1 Hz, 1H); 8.32 (dd, *J* = 7.5 and 1.1 Hz, 1H); 7.88 (dd, *J* = 8.0 and 7.5 Hz, 1H); 7.02 (dd, *J* = 7.7 and 7.7 Hz, 1H); 6.83–6.92 (ovl. m, 3H); 3.57 (s, 2H); 3.51 (tm, *J* = 4.7 Hz, 4H); 3.00 (tm, *J* = 4.7 Hz, 2H); 2.87 (tm, *J* = 4.7 Hz, 2H); 2.13 (s, 3H).

#### 4.2.35 2-(4-fluorophenyl)-1-(4-(isoquinolin-5-ylsulfonyl)piperazin-1-yl)ethan-1-one (**29**)

LCMS[MH+]: 414.3, MW calc: 413.121, Rt: 3.13 min.

^1^H NMR (300 MHz, DMSO‑*d*_6_) *δ*: 9.50 (s, 1H); 8.69 (d, *J* = 6.1 Hz, 1H); 8.52 (dd, *J* = 8.0 and 1.1 Hz, 1H); 8.42 (d, *J* = 6.1 Hz, 1H); 8.35 (dd, *J* = 7.5 and 1.1 Hz, 1H); 7.88 (dd, *J* = 8.0 and 7.5 Hz, 1H); 7.12 (ddm, *J* = 8.8 and 8.8 Hz, 2H); 7.01 (ddm, *J* = 8.8 and 5.7 Hz, 2H); 3.62 (s, 2H); 3.52 (tm, *J* = 4.7 Hz, 4H); 3.03 (tm, *J* = 4.7 Hz, 4H).

#### 4.2.36 1-(4-(isoquinolin-5-ylsulfonyl)piperazin-1-yl)-2-(4-nitrophenyl)ethan-1-one (**30**)

LCMS[MH+]: 441.2, MW calc: 440.115, Rt: 3.09 min.

^1^H NMR (300 MHz, DMSO‑*d*_6_) *δ*: 9.50 (s, 1H); 8.70 (d, *J* = 6.1 Hz, 1H); 8.52 (dd, *J* = 8.0 and 1.1 Hz, 1H); 8.44 (d, *J* = 6.1 Hz, 1H); 8.36 (dd, *J* = 7.5 and 1.1 Hz, 1H); 8.09 (dm, *J* = 8.6 Hz, 2H); 7.88 (dd, *J* = 8.0 and 7.5 Hz, 1H); 7.39 (dm, *J* = 8.6 Hz, 2H); 3.82 (s, 2H); 3.49–3.60 (m, 4H); 3.08 (tm, *J* = 4.7 Hz, 4H).

#### 4.2.37 N-(3-cyanophenyl)-4-(isoquinolin-5-ylsulfonyl)piperazine-1-carboxamide (**31**)

LCMS[MH+]: 422.2, MW calc: 421.121, Rt: 2.98 min.

^1^H NMR (300 MHz, DMSO‑*d*_6_) *δ*: 9.50 (s, 1H); 8.85 (s, 1H); 8.71 (d, *J* = 6.1 Hz, 1H); 8.52 (dd, *J* = 8.0 and 1.1 Hz, 1H); 8.48 (d, *J* = 6.1 Hz, 1H); 8.39 (dd, *J* = 7.5 and 1.1 Hz, 1H); 7.89 (dd, *J* = 8.0 and 7.5 Hz, 1H); 7.82 (dd, *J* = 2.0 amd 2.0 Hz, 1H); 7.63 (ddd, *J* = 8.2, 2.0 and 1.5 Hz, 1H); 7.40 (dd, *J* = 8.2 and 7.8 Hz, 1H); 7.35 (ddd, *J* = 7.8, 2.0 and 1.5 Hz, 1H); 3.51 (tm, *J* = 4.7 Hz, 4H); 3.13 (tm, *J* = 4.7 Hz, 4H).

#### 4.2.38 4-(Isoquinolin-5-ylsulfonyl)—N-(3-(trifluoromethyl)phenyl)piperazine-1-carboxamide (**32**)

LCMS[MH+]: 465.2, MW calc: 464.113, Rt: 3.6 min.

^1^H NMR (300 MHz, DMSO‑*d*_6_) *δ*: 9.50 (s, 1H); 8.82 (s, 1H); 8.71 (d, *J* = 6.1 Hz, 1H); 8.51 (dd, *J* = 8.0 and 1.1 Hz, 1H); 8.48 (d, *J* = 6.1 Hz, 1H); 8.39 (dd, *J* = 7.5 and 1.1 Hz, 1H); 7.89 (dd, *J* = 8.0 and 7.5 Hz, 1H); 7.80 (ddm, *J* = 2.0 amd 2.0 Hz, 1H); 7.63 (dddm, *J* = 8.5, 2.0 and 1.5 Hz, 1H); 7.42 (dd, *J* = 8.5 and 7.6 Hz, 1H); 7.23 (ddd, *J* = 7.6, 2.0 and 1.5 Hz, 1H); 3.52 (tm, *J* = 4.7 Hz, 4H); 3.14 (tm, *J* = 4.7 Hz, 4H).

#### 4.2.39 N-(4-chlorophenyl)-4-(isoquinolin-5-ylsulfonyl)piperazine-1-carboxamide (**33**)

LCMS[MH+]: 431.2, MW calc: 430.087, Rt: 3.35 min.

^1^H NMR (300 MHz, DMSO‑*d*_6_) *δ*: 9.50 (s, 1H); 8.71 (d, *J* = 6.1 Hz, 1H); 8.64 (s, 1H); 8.52 (dd, *J* = 8.0 and 1.1 Hz, 1H); 8.48 (d, *J* = 6.1 Hz, 1H); 8.38 (dd, *J* = 7.5 and 1.1 Hz, 1H); 7.89 (dd, *J* = 8.0 and 7.5 Hz, 1H); 7.38 (dm, *J* = 8.9 Hz, 2H); 7.23 (dm, *J* = 8.9 Hz, 2H); 3.49 (tm, *J* = 4.7 Hz, 4H); 3.11 (tm, *J* = 4.7 Hz, 4H).

#### 4.2.40 N-(4-chloro-3-(trifluoromethyl)phenyl)-4-(isoquinolin-5-ylsulfonyl)piperazine-1-carboxamide (**34**)

LCMS[MH+]: 476.0, MW calc: 475.072, Rt: 3.56 min.

^1^H NMR (300 MHz, DMSO‑*d*_6_) *δ*: 9.50 (s, 1H); 8.94 (s, 1H); 8.70 (d, *J* = 6.2 Hz, 1H); 8.52 (dd, *J* = 8.3 and 1.1 Hz, 1H); 8.48 (d, *J* = 6.2 Hz, 1H); 8.39 (dd, *J* = 7.4 and 1.1 Hz, 1H); 7.93 (d, *J* = 2.6 Hz, 1H); 7.89 (dd, *J* = 8.3 and 7.4 Hz, 1H); 7.67 (dd, *J* = 8.8 and 2.6 Hz, 1H); 7.52 (d, *J* = 8.8 Hz, 1H); 3.52 (tm, *J* = 4.7 Hz, 4H); 3.13 (tm, *J* = 4.7 Hz, 4H).

#### 4.2.41 N-(4-chloro-3-nitrophenyl)-4-(isoquinolin-5-ylsulfonyl)piperazine-1-carboxamide (**35**)

LCMS[MH+]:477.01, MW calc: 475.911, Rt: 3.66 min.

^1^H NMR (300 MHz, DMSO‑*d*_6_) *δ*: 9.50 (s, 1H); 8.71 (d, *J* = 6.2 Hz, 1H); 8.52 (dd, *J* = 8.3 and 1.1 Hz, 1H); 8.48 (d, *J* = 6.2 Hz, 1H); 8.39 (dd, *J* = 7.4 and 1.1 Hz, 1H); 7.90 (dd, *J* = 8.3 and 7.4 Hz, 1H); 7.85 (s, 1H); 7.65 (d, *J* = 2.3 Hz, 1H); 6.99 (dd, *J* = 8.7 and 2.3 Hz, 1H); 6.95 (d, *J* = 8.7 Hz, 1H); 3.72 (s, 3H); 3.46 (tm, *J* = 4.7 Hz, 4H); 3.11 (tm, *J* = 4.7 Hz, 4H).

#### 4.2.42 N-(3-chloro-4-fluorophenyl)-4-(isoquinolin-5-ylsulfonyl)piperazine-1-carboxamide (**36**)

LCMS[MH+]: 449.2, MW calc: 448.077, Rt: 3.43 min.

^1^H NMR (300 MHz, DMSO‑*d*_6_) *δ*: 9.50 (s, 1H); 8.71 (d, *J* = 6.1 Hz, 1H); 8.70 (s, 1H); 8.52 (dd, *J* = 8.0 and 1.1 Hz, 1H); 8.48 (d, *J* = 6.1 Hz, 1H); 8.38 (dd, *J* = 7.5 and 1.1 Hz, 1H); 7.89 (dd, *J* = 8.0 and 7.5 Hz, 1H); 7.63 (dd, *J* = 6.9 amd 2.3 Hz, 1H); 7.29 (dddm, *J* = 9.0, 4.7 and 2.3 Hz, 1H); 7.24 (dd, *J* = 9.0 and 9.0 Hz, 1H); 3.49 (tm, *J* = 4.7 Hz, 4H); 3.11 (tm, *J* = 4.7 Hz, 4H).

#### 4.2.43 N-(2,3-dichlorophenyl)-4-(isoquinolin-5-ylsulfonyl)piperazine-1-carboxamide (**37**)

LCMS[MH+]: 465.2, MW calc: 464.047, Rt: 3.48 min.

^1^H NMR (300 MHz, DMSO‑*d*_6_) *δ*: 9.51 (s, 1H); 8.71 (d, *J* = 6.1 Hz, 1H); 8.53 (dd, *J* = 8.0 and 1.1 Hz, 1H); 8.48 (d, *J* = 6.1 Hz, 1H); 8.45 (s, 1H); 8.39 (dd, *J* = 7.5 and 1.1 Hz, 1H); 7.90 (dd, *J* = 8.0 and 7.5 Hz, 1H); 7.36 (dd, *J* = 8.0 and 1.9 Hz, 1H); 7.33 (dd, *J* = 7.8 and 1.9 Hz, 1H); 7.25 (dd, *J* = 8.0 and 7.8 Hz, 1H); 3.30 (tm, *J* = 4.7 Hz, 4H); 3.12 (tm, *J* = 4.7 Hz, 4H).

#### 4.2.44 N-(3-chloro-4-methylphenyl)-4-(isoquinolin-5-ylsulfonyl)piperazine-1-carboxamide (**38**)

LCMS[MH+]: 445.1, MW calc: 444.102, Rt: 3.63 min.

^1^H NMR (300 MHz, DMSO‑*d*_6_) *δ*: 9.50 (s, 1H); 8.70 (d, *J* = 6.2 Hz, 1H); 8.59 (s, 1H); 8.52 (dd, *J* = 8.3 and 1.1 Hz, 1H); 8.48 (d, *J* = 6.2 Hz, 1H); 8.39 (dd, *J* = 7.4 and 1.1 Hz, 1H); 7.89 (dd, *J* = 8.3 and 7.4 Hz, 1H); 7.51 (d, *J* = 1.7 Hz, 1H); 7.19 (dd, *J* = 8.5 and 1.7 Hz, 1H); 7.14 (d, *J* = 8.5 Hz, 1H); 3.48 (tm, *J* = 4.7 Hz, 4H); 3.11 (tm, *J* = 4.7 Hz, 4H); 2.20 (s, 3H).

#### 4.2.45 4-(Isoquinolin-5-ylsulfonyl)—N-(4-methyl-2-nitrophenyl)piperazine-1-carboxamide (**39**)

LCMS[MH+]: 456.3, MW calc: 455.126, Rt: 3.56 min.

^1^H NMR (300 MHz, DMSO‑*d*_6_) *δ*: 9.52 (s, 1H); 9.09 (s, 1H); 8.72 (d, *J* = 6.2 Hz, 1H); 8.54 (dd, *J* = 8.3 and 1.1 Hz, 1H); 8.50 (d, *J* = 6.2 Hz, 1H); 8.41 (dd, *J* = 7.4 and 1.1 Hz, 1H); 7.91 (dd, *J* = 8.3 and 7.4 Hz, 1H); 7.68 (dm, *J* ∼ 1.0 Hz, 1H); 7.35–7.43 (ovl. m, 2H); 3.50 (tm, *J* = 4.7 Hz, 4H); 3.12 (tm, *J* = 4.7 Hz, 4H); 2.29 (s, 3H).

#### 4.2.46 N-(2-chloro-6-methylphenyl)-4-(isoquinolin-5-ylsulfonyl)piperazine-1-carboxamide (**40**)

LCMS[MH+]: 445.2, MW calc: 444.102, Rt: 3.01 min.

^1^H NMR (300 MHz, DMSO‑*d*_6_) *δ*: 9.52 (s, 1H); 8.71 (d, *J* = 6.1 Hz, 1H); 8.54 (dd, *J* = 8.0 and 1.1 Hz, 1H); 8.50 (d, *J* = 6.1 Hz, 1H); 8.39 (dd, *J* = 7.5 and 1.1 Hz, 1H); 8.17 (s, 1H); 7.91 (dd, *J* = 8.0 and 7.5 Hz, 1H); 7.21 (dd, *J* = 7.3 and 2.2 Hz, 1H); 7.13 (dd, *J* = 7.5 and 2.2 Hz, 1H); 7.10 (dd, *J* = 7.5 and 7.3 Hz, 1H); 3.51 (tm, *J* = 4.7 Hz, 4H); 3.07 (tm, *J* = 4.7 Hz, 4H); 2.00 (s, 3H).

#### 4.2.47 N-(benzo[d] [1,3]dioxol-5-yl)-4-(isoquinolin-5-ylsulfonyl)piperazine-1-carboxamide (**41**)

LCMS[MH+]: 441.1, MW calc: 440.115, Rt: 2.89 min.

^1^H NMR (300 MHz, DMSO‑*d*_6_) *δ*: 9.51 (s, 1H); 8.70 (d, *J* = 6.2 Hz, 1H); 8.52 (dd, *J* = 8.2 and 1.2 Hz, 1H); 8.49 (d, *J* = 6.2 Hz, 1H); 8.41 (s, 1H)8.39 (dd, *J* = 7.3 and 1.2 Hz, 1H); 7.89 (dd, *J* = 8.2 and 7.3 Hz, 1H); 7.01 (d, *J* ∼ 1.5 Hz, 1H); 6.68–6.77 (ovl. m, 2H); 5.90 (s, 2H); 3.46 (m, 4H); 3.10 (m, 4H).

#### 4.2.48 *N*-(benzo[*d*] [1,3]dioxol-5-yl)-4-(isoquinolin-5-ylsulfonyl)piperazine-1-*carbothioamide (****42****)*

LCMS[MH+]: 457.3, MW calc: 456.093, Rt: 3.21 min.

^1^H NMR (300 MHz, DMSO‑*d*_6_) *δ*: 9.51 (s, 1H); 9.23 (s, 1H); 8.71 (d, *J* = 6.1 Hz, 1H); 8.53 (dd, *J* = 8.0 and 1.1 Hz, 1H); 8.49 (d, *J* = 6.1 Hz, 1H); 8.39 (dd, *J* = 7.5 and 1.1 Hz, 1H); 7.90 (dd, *J* = 8.0 and 7.5 Hz, 1H); 6.78 (d, *J* = 1.5 Hz, 1H); 6.77 (d, *J* = 8.3 Hz, 1H); 6.55 (dd, *J* = 8.3 and 1.5 Hz, 1H); 5.97 (s, 2H); 3.93 (tm, *J* = 4.7 Hz, 4H); 3.16 (tm, *J* = 4.7 Hz, 4H).

#### 4.2.49 4-[(Isoquinoline-5-sulfonyl) amino]—N-(2H-1,3-benzodioxol-5-yl)piperidine-1-carboxamide (**43**)

LCMS[MH+]: 471.3, MW calc: 470.108, Rt: 2.99 min.

^1^H NMR (300 MHz, DMSO‑*d*_6_) *δ*: 9.49 (s, 1H); 9.04 (s, 1H); 8.71 (d, *J* = 6.1 Hz, 1H); 8.45 (d, *J* = 6.1 Hz, 1H); 8.44 (dd, *J* = 8.0 and 1.1 Hz, 1H); 8.41 (dd, *J* = 7.5 and 1.1 Hz, 1H); 8.25 (d, *J* = 8.0 Hz, 1H); 7.85 (dd, *J* = 8.0 and 7.5 Hz, 1H); 6.79 (d, *J* = 8.3 Hz, 1H); 6.79 (d, *J* = 1.8 Hz, 1H); 6.56 (dd, *J* = 8.3 and 1.8 Hz, 1H); 5.98 (s, 2H); 4.37 (dm, *J* ∼ 13.5 Hz, 2H); 3.37 (m, 1H); 3.09 (ddm, *J* ∼ 13.5 and 11.0 Hz, 2H); 1.49 (dm, *J* = 12.0 Hz, 2H); 1.30 (dddd, *J* = 12.0, 11.0, 10.0 and 1.5 Hz, 2H).

#### 4.2.50 4-(Isoquinoline-5-sulfonyl)—N-(2H-1,3-benzodioxol-5-yl)-1,4-diazepane-1-carboxamide (**44**)

LCMS[MH+]: 455.3, MW calc: 454.131, Rt: 2.74 min.

^1^H NMR (300 MHz, DMSO‑*d*_6_) *δ*: 9.46 (s, 1H); 8.66 (d, *J* = 6.1 Hz, 1H); 8.44 (dd, *J* = 8.0 and 1.1 Hz, 1H); 8.33 (d, *J* = 6.1 Hz, 1H); 8.32 (dd, *J* = 7.5 and 1.1 Hz, 1H); 8.14 (s, 1H); 7.82 (dd, *J* = 8.0 and 7.5 Hz, 1H); 7.08 (d, *J* ∼ 1.5 Hz, 1H); 6.74–6.82 (ovl. m, 2H); 5.94 (s, 2H); 3.60 (tm, *J* ∼ 5.5 Hz, 2H); 3.47–3.55 (ovl. m, 4H); 3.41 (tm, *J* ∼ 5.5 Hz, 2H); 1.78 (tt, *J* ∼ 5.5 and 5.5 Hz, 2H).

#### 4.2.51 N-(benzo[d] [1,3]dioxol-5-yl)-4-(isoquinolin-5-ylsulfonyl)-2-methylpiperazine-1-carboxamide (**45**)

LCMS[MH+]: 455.2, MW calc: 454.131, Rt: 3.08 min.

^1^H NMR (300 MHz, DMSO‑*d*_6_) *δ*: 9.50 (s, 1H); 8.71 (d, *J* = 6.1 Hz, 1H); 8.52 (dd, *J* = 8.0 and 1.1 Hz, 1H); 8.45 (d, *J* = 6.1 Hz, 1H); 8.38 (dd, *J* = 7.5 and 1.1 Hz, 1H); 8.35 (s, 1H); 7.89 (dd, *J* = 8.0 and 7.5 Hz, 1H); 7.02 (d, *J* ∼ 1.5 Hz, 1H); 6.70–6.77 (m, 2H); 5.91 (s, 2H); 4.37 (qdd, *J* = 6.6, 3.4 and 1.5 Hz, 1H); 3.92 (ddd, *J* = 14.0, 1.5 and 1.5 Hz, 1H); 3.77 (ddd, *J* = 11.5, 1.5 and 1.5 Hz, 1H); 3.53 (ddd, *J* = 12.0, 1.5 and 1.5 Hz, 1H); 3.08 (ddd, *J* = 14.0, 11.3 and 3.5 Hz, 1H); 2.64 (dd, *J* = 12.0 and 3.4 Hz, 1H); 2.49 (ddd, *J* = 12.0, 11.5 and 3.5 Hz, 1H); 1.05 (d, *J* = 6.6 Hz, 3H).

#### 4.2.52 N-benzyl-4-(isoquinolin-5-ylsulfonyl)piperazine-1-carboxamide (**46**)

LCMS[MH+]: 411.3, MW calc: 410.141, Rt: 2.93 min.

^1^H NMR (300 MHz, DMSO‑*d*_6_) *δ*: 9.51 (s, 1H); 8.70 (d, *J* = 6.2 Hz, 1H); 8.52 (dd, *J* = 8.2 and 1.2 Hz, 1H); 8.46 (d, *J* = 6.2 Hz, 1H); 8.41 (s, 1H)8.37 (dd, *J* = 7.3 and 1.2 Hz, 1H); 7.89 (dd, *J* = 8.2 and 7.3 Hz, 1H); 7.05–7.25 (ovl. m, 6H); 4.14 (d, *J* ∼ 5.5 Hz, 2H); 3.38 (m, 4H); 3.04 (m, 4H).

^13^C NMR (75 MHz, DMSO‑*d*_6_) *δ*: 157.1; 153.8; 145.1; 140.7; 134.7; 134.5; 131.2; 130.9; 128.9; 128.2; 127.0; 126.7; 126.6; 117.2; 45.5; 43.5; 43.2.

#### 4.2.53 4-(Isoquinolin-5-ylsulfonyl)—N-(3-methoxybenzyl)piperazine-1-carboxamide (**47**)

LCMS[MH+]: 440.2, MW calc: 440.152, Rt: 3.02 min.

^1^H NMR (300 MHz, DMSO‑*d*_6_) *δ*: 9.51 (s, 1H); 8.70 (d, *J* = 6.1 Hz, 1H); 8.53 (dd, *J* = 8.0 and 1.1 Hz, 1H); 8.47 (d, *J* = 6.1 Hz, 1H); 8.38 (dd, *J* = 7.5 and 1.1 Hz, 1H); 7.90 (dd, *J* = 8.0 and 7.5 Hz, 1H); 7.15 (ddm, *J* ∼ 8.0 and 8.0 Hz, 1H); 6.97 (dm, *J* ∼ 8.0 Hz, 1H); 6.89 (dm, *J* ∼ 8.0 Hz, 1H); 6.86 (t, *J* = 5.0 Hz, 1H); 6.76 (ddm, *J* ∼ 8.0 and 8.0 Hz, 1H); 4.11 (d, *J* = 5.0 Hz, 2H); 3.72 (s, 3H); 3.39 (tm, *J* = 4.7 Hz, 4H); 3.05 (tm, *J* = 4.7 Hz, 4H).

^13^C NMR (75 MHz, DMSO‑*d*_6_) *δ*: 157.2, 156.5, 153.7, 145.1, 134.6, 134.5, 131.2, 131.0, 128.9, 128.0, 127.6, 127.1, 126.7, 120.0, 117.2, 110.3, 55.3, 45.5, 43.3, 38.4.

#### 4.2.54 4-(Isoquinolin-5-ylsulfonyl)—N-(2-methylbenzyl)piperazine-1-carboxamide (**48**)

LCMS[MH+]: 425.3, MW calc: 424.157, Rt: 3.18 min.

^1^H NMR (300 MHz, DMSO‑*d*_6_) *δ*: 9.51 (s, 1H); 8.70 (d, *J* = 6.1 Hz, 1H); 8.53 (dd, *J* = 8.0 and 1.1 Hz, 1H); 8.46 (d, *J* = 6.1 Hz, 1H); 8.37 (dd, *J* = 7.5 and 1.1 Hz, 1H); 7.89 (dd, *J* = 8.0 and 7.5 Hz, 1H); 6.97–7.09 (ovl. m, 4H); 6.92 (t, *J* = 5.5 Hz, 1H); 4.11 (d, *J* = 5.5 Hz, 2H); 3.39 (tm, *J* = 4.7 Hz, 4H); 3.03 (tm, *J* = 4.7 Hz, 4H); 2.16 (s, 3H).

^13^C NMR (75 MHz, DMSO‑*d*_6_) *δ*: 157.0; 153.7; 145.1; 138.1; 135.3; 134.7; 134.5; 131.2; 130.8; 129.8; 128.9; 127.1; 126.7; 126.5; 125.6; 117.2; 45.5; 43.3; 41.5; 18.7.

#### 4.2.55 Benzyl 4-(isoquinolin-5-ylsulfonyl)piperazine-1-carboxylate (**49**)

LCMS[MH+]: 412.3, MW calc: 411.25, Rt: 3.53 min.

^1^H NMR (300 MHz, DMSO‑*d*_6_) *δ*: 9.72 (s, 1H); 8.74 (d, *J* = 6.2 Hz, 1H); 8.65 (dd, *J* = 8.2 and 1.1 Hz, 1H); 8.62 (d, *J* = 6.2 Hz, 1H); 8.48 (dd, *J* = 7.4 and 1.1 Hz, 1H); 7.99 (dd, *J* = 8.2 and 7.4 Hz, 1H); 7.23–7.38 (ovl. m, 5H); 5.00 (s, 2H); 3.44 (tm, *J* = 4.7 Hz, 4H); 3.12 (tm, *J* = 4.7 Hz, 4H).

### 4.3 Drug susceptibility testing and target-based whole-cell screening against *M. tuberculosis*

An Alamar Blue fluorescence-based broth microdilution assay was used to assess minimum inhibitory concentration (MIC) of compounds against *M. tuberculosis* strains, as described previously [5,23].

### 4.4 IMPDH biochemical assay

The *Mtb* IMPDH was overexpressed and purified as described previously [5]. The kinetics of inhibition of *Mtb* GuaB2 by compound **1** and its analogs were performed as described previously [5].

### 4.5 IMPDH purification, crystallization, and data collection of M. thermoresistible (Mth)

IMPDH was expressed, purified, and crystallized as previously described [5,8]. Briefly, hexahistidine tagged *Mth* IMPDH ΔCBS in the pHat2 vector was expressed overnight in BL21 DE3 (NEB) cells at 18 °C by the addition of 500 μM IPTG. Cells were lysed in 50 mM Hepes, pH 8.0, 500 mM NaCl, 5% glycerol, 10 mM β-mercaptoethanol, and 20 mM imidazole, and the recombinant protein purified by immobilized metal affinity chromatography using a gradient of up to 300 mM imidazole over a Hi-Trap IMAC FF column (GE Healthcare) charged with nickel. The hexahistidine tag was cleaved by TEV protease, and purified through a negative nickel gravity-flow purification step [24]. Following size exclusion chromatography on a Superdex 200 gel filtration column equilibrated in 20 mM Hepes pH 8.0, 500 mM NaCl, 5% glycerol, and 1 mM TCEP, the recombinant *Mth* IMPDH ΔCBS was concentrated to 12.5 mg/mL for crystallization.

*Mth* IMPDH ΔCBS protein was crystallized in 1 μL + 1 μL hanging drops with 100 mM sodium acetate, pH 5.5, 200 mM calcium chloride, and 8–14% isopropanol. Crystals were soaked overnight in drops of well solution +5 mM IMP and 5 mM compound solubilized in 100% DMSO. Crystals were cryoprotected by passing through drops containing well solution +25% glycerol and flash-frozen in liquid nitrogen. Data were collected from the crystals at Diamond Light Source beamline I24.

### 4.6 Structure solution, ligand fitting, and refinement

Data were processed using XDS [25] and Pointless (CCP4). To solve the structure, molecular replacement was performed with Phenix Phaser [26] using a previously solved IMP-bound *Mth* GuaB2 ΔCBS structure as a probe (unpublished data). Refinement was performed using Phenix.refine and manually in Coot [27]. IMP and the inhibitors were sequentially fitted into the density using the LigandFit function of Phenix, and the structures were manually refined further using Coot. Information regarding the crystallographic statistics can be found in Table 5. Protein–ligand interactions were visualized using Arpeggio [17] and their importance quantified by the mCSM suite [[28], [29], [30], [31], [32], [33], [34]]. Pharmacophore analysis performed as previously described [35]. All figures made using Pymol (Schrodinger).

The atomic coordinates and experimental data for the *Mth* GuaB2 ΔCBS structure in complex with the compounds have been deposited in the Protein Data Bank under the accession numbers: 6D4Q (Compound **14**); 6D4R (Compound **18**); 6D4V (Compound **22**); 6D4U (Compound **27**); 6D4W (Compound **35**); 6D4S (Compound **37**); 6D4T (Compound **45**).

### 4.7 Computational modelling

Docking of ligands was performed using Surflex running in Sybyl-X 2.1.1 and Glide running in Schrodinger 2017 as previously described [24,[36], [37], [38], [39], [40], [41], [42], [43]]. Ligand sites were defined on the basis of the crystallographic structures using a threshold of 0.5 and bloat value of 2.0. Compound poses were evaluated using Arpeggio [17] and CSM-Lig [44]. The pkCSM model was used to calculate the Caco-2 permeability [27]. A compound is considered to be Caco-2 permeable with a permeability coefficient (Papp) > 8 × 10^−6^ cm/s. For the pkCSM predictive model, high Caco-2 permeability would translate in predicted values > 0.9.

## Appendix A Supplementary data

Table S1, related to synthesis of compound **1** and its analogs.

Table S2, describing the IUPAC names, LogP, and predicted Caco-2 values of the compounds.

Analytical data for compounds **1**, **21**, **47** and **48**.

***PDB ID Codes***: The structures have been deposited in the Protein Data Bank under the accession numbers: 6D4Q (Compound 1**4**); 6D4R (Compound 1**8**); 6D4V (Compound **22**); 6D4U (Compound **27**); 6D4W (Compound **35**); 6D4S (Compound **37**); 6D4T (Compound **45**);
