# Supplementary material for: Synthesis and Structure–Activity relationship of 1-(5-isoquinolinesulfonyl)piperazine analogues as inhibitors of Mycobacterium tuberculosis IMPDH
Source: Eur J Med Chem. 2019 Jul 15;174:309–29. doi: 10.1016/j.ejmech.2019.04.027 (PMC6990405; doi:10.1016/j.ejmech.2019.04.027)
Supplement: Multimedia component 2 [file mmc2.pdf]

SUPPLEMENTARY INFORMATION

Synthesis and Structure–Activity Relationship of 1-(5-isoquinolinesulfonyl)piperazine Analogues as Inhibitors of *Mycobacterium tuberculosis* IMPDH

Vinayak Singh[1,2,3*], Angela Pacitto[4], Stefano Donini[5], Davide M. Ferraris[5], Sándor Boros[7], Eszter Illyés[7], Bálint Szokol[7], Menico Rizzi[5], Tom L. Blundell[4], David B. Ascher[4,6], Janos Pato[7], and Valerie Mizrahi[2*]

[1]H3D Drug Discovery and Development Centre, Department of Drug Discovery and Development & Institute of Infectious Disease and Molecular Medicine, University of Cape Town, Rondebosch 7701, Cape Town, South Africa

[2]MRC/NHLS/UCT Molecular Mycobacteriology Research Unit, DST/NRF Centre of Excellence for Biomedical TB Research & Wellcome Centre for Infectious Diseases Research in Africa, Institute of Infectious Disease and Molecular Medicine & Department of Pathology, University of Cape Town, Anzio Road, Observatory 7925, South Africa

[3]South African Medical Research Council Drug Discovery and Development Research Unit, Department of Chemistry and Institute of Infectious Disease and Molecular Medicine, University of Cape Town, Rondebosch 7701, Cape Town, South Africa

[4]Department of Biochemistry, University of Cambridge, 80 Tennis Court Road, Cambridge CB2 1GA, United Kingdom

[5]Dipartimento di Scienze del Farmaco, University of Piemonte Orientale, Via Bovio 6, 28100 Novara, Italy

[6]Department of Biochemistry and Molecular Biology, University of Melbourne, Bio21 Institute, 30 Flemington Road, Parkville, 3052, Australia

[7]Vichem Chemie Research Ltd, Rákóczi utca 5, Veszprém, 8200, Hungary

Table of Contents

1. Table S1

2. Table S2 (attached as a separate excel spreadsheet document)

3. Analytical data for compounds **1**, **4**, **21**, **46**, **47** and **48**.

Table S1. Synthesis of compound 1 and its analogs (See the Scheme 2 for codes)

Cmpd No.	Sulfochloride	Amine	Cyclic	Method
**1**	S1	A1	C 1	(v)
**2**	S1	A5	C 1	(vi)
**3**	S1	A4	C 1	(vi)
**4**	S1	A6	C 1	(vi)
**5**	S1	A3	C 1	(vi)
**6**	S2	A1	C 1	(vi)
**7**	S3	A1	C 1	(i)
**8**	S4	A1	C 1	(i)
**9**	S1	A5	C 5	(vi)
**10**	S1	A5	C 6	(vi)
**11**	S1	A5	C 7	(vi)
**12**	S1	A1	C 2	(vii)
**13**	S1	A1	C 3	(vii)
**14**	S1	A1	C 4	(vii)
**15**	S1	A1	C 8	(v)
**16**	S1	A1	C 11	(v)
**17**	S1	A1	C 12	(v)
**18**	S1	A1	C 9	(v)
**19**	S1	A1	C 10	(v)
**20**	S1	A1	C 13	(v)
**21**	S1	A5	C 10	(v)
**22**	S1	A1	P 8	(vii)

**23**	S1	A1	P 9	(vii)
**24**	S1	A1	P 3	(vii)
**25**	S1	A1	P 1	(vii)
**26**	S1	A1	P 5	(vii)
**27**	S1	A1	P 4	(vii)
**28**	S1	A1	P 7	(vii)
**29**	S1	A1	P 6	(vii)
**30**	S1	A1	P 2	(vii)
**31**	S1	A1	I 9	(viii)
**32**	S1	A1	I 6	(viii)
**33**	S1	A1	I 7	(viii)
**34**	S1	A1	I 5	(v)
**35**	S1	A1	I 1	(viii)
**36**	S1	A1	I 2	(viii)
**37**	S1	A1	I 3	(viii)
**38**	S1	A1	I 4	(viii)
**39**	S1	A1	I 10	(v)
**40**	S1	A1	I 8	(viii)
**41**	S1	A1	I 11	(viii)
**42**	S1	A1	I 12	(viii)
**43**	S1	A3	I 11	(viii)
**44**	S1	A2	I 11	(viii)
**45**	S1	A4	I 11	(vi)
**46**	S1	A1	B 1	(viii)
**47**	S1	A1	B 3	(viii)
**48**	S1	A1	B 2	(viii)
**49**	S1	A1	B 4	(i)

Analytical data for compounds 1, 4, 21, 46, 47 and **48**.

*Compound **1***

Formula Weight: 387,4958; Exact Mass: 387,16166238
Molecular Formula: $C_{20}H_{25}N_3O_3S$

^{1}H-NMR chemical shifts δ [ppm]

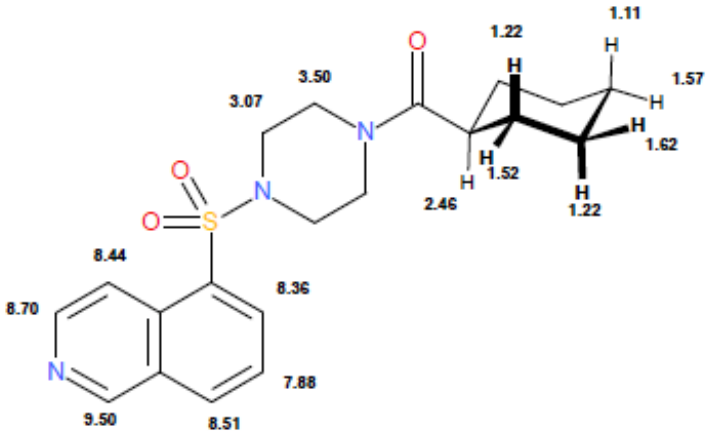

Characterictic J(H,H) coupling constants [Hz]

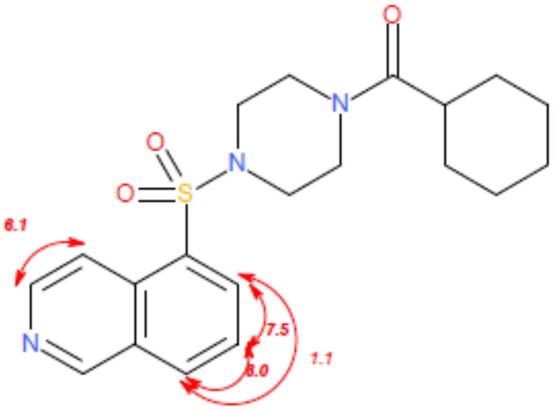

^{13}C-NMR chemical shifts δ [ppm]

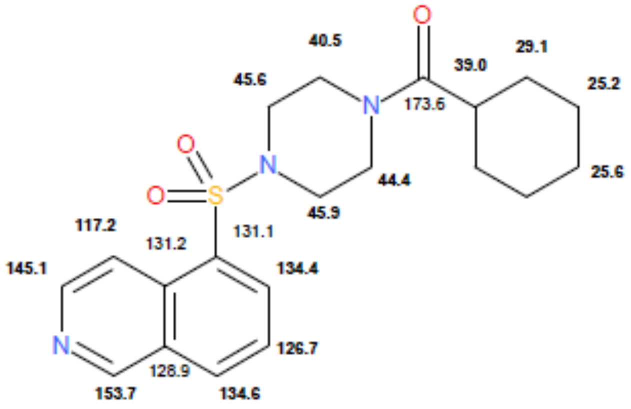

Characteristic heteronuclear long-range couplings detected by HMBC experiment H ⟶ C

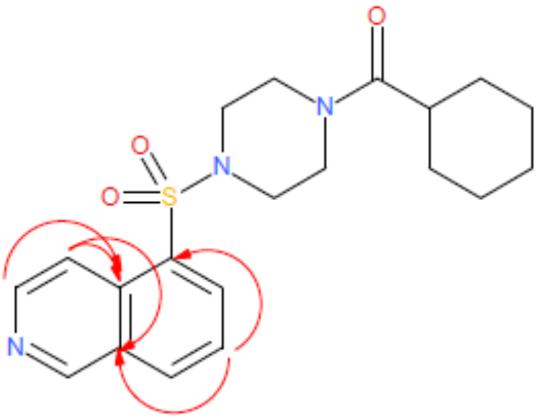

^{1}H NMR:

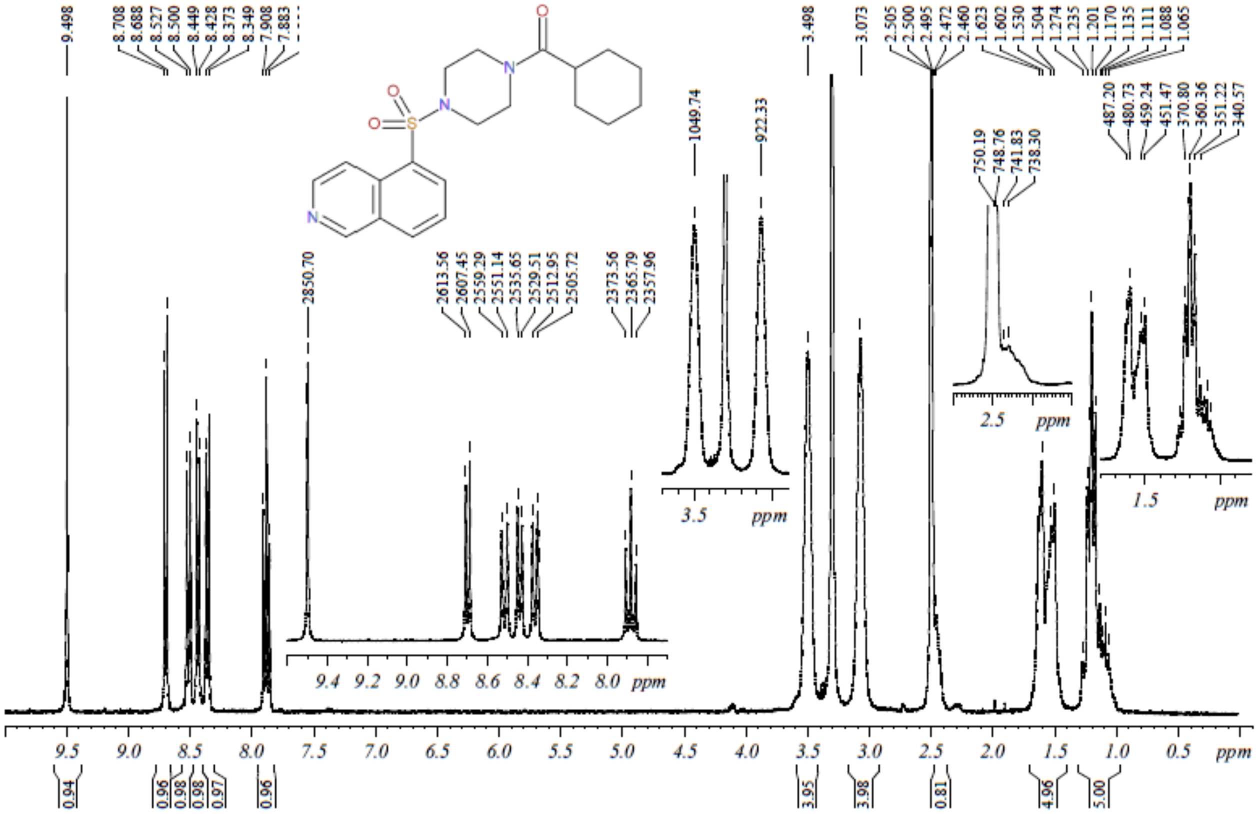

Total correlation spectroscopy (TOCSY):

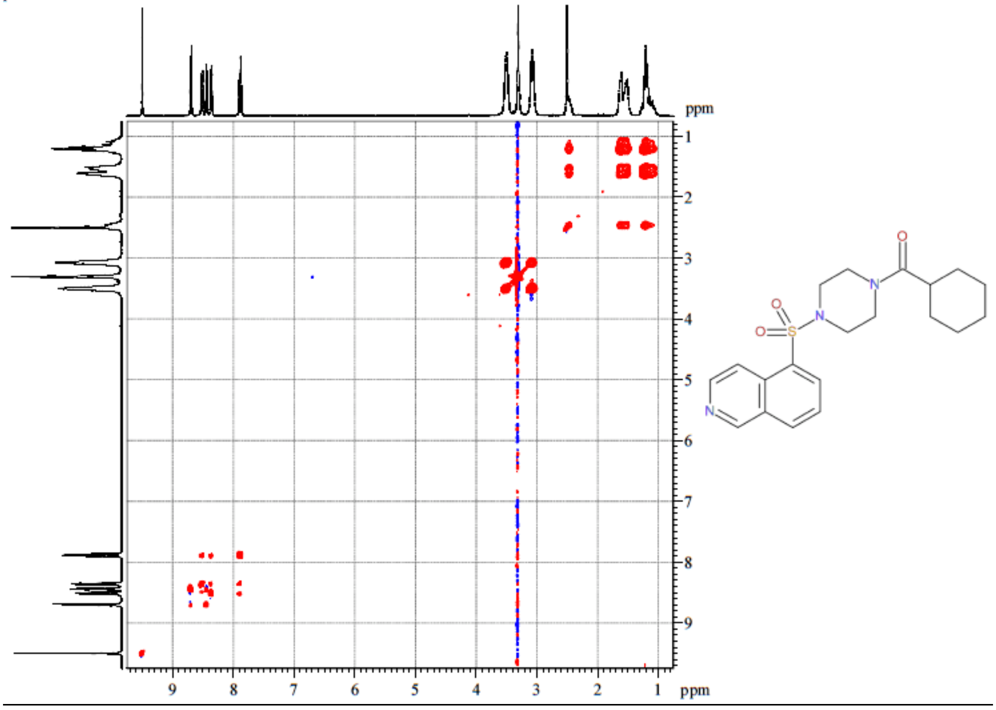

^{13}C-NMR:

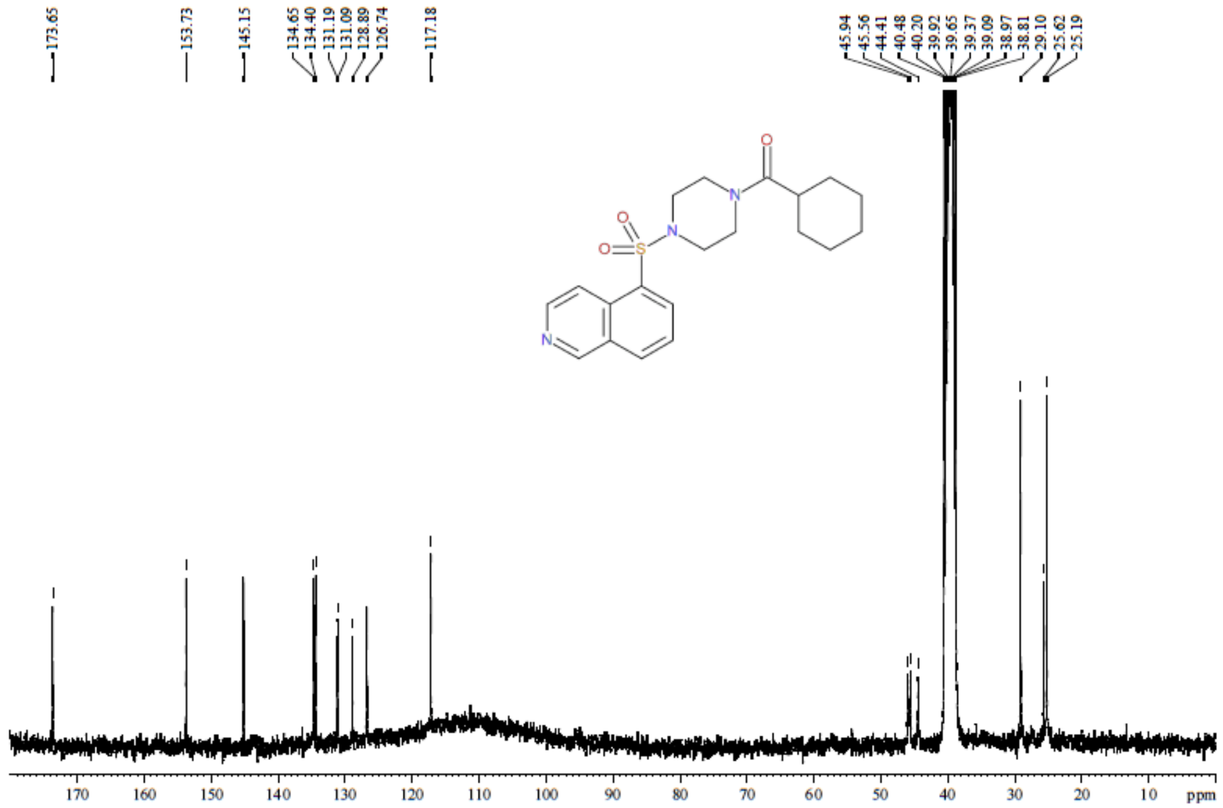

Heteronuclear single quantum coherence (HSQC):

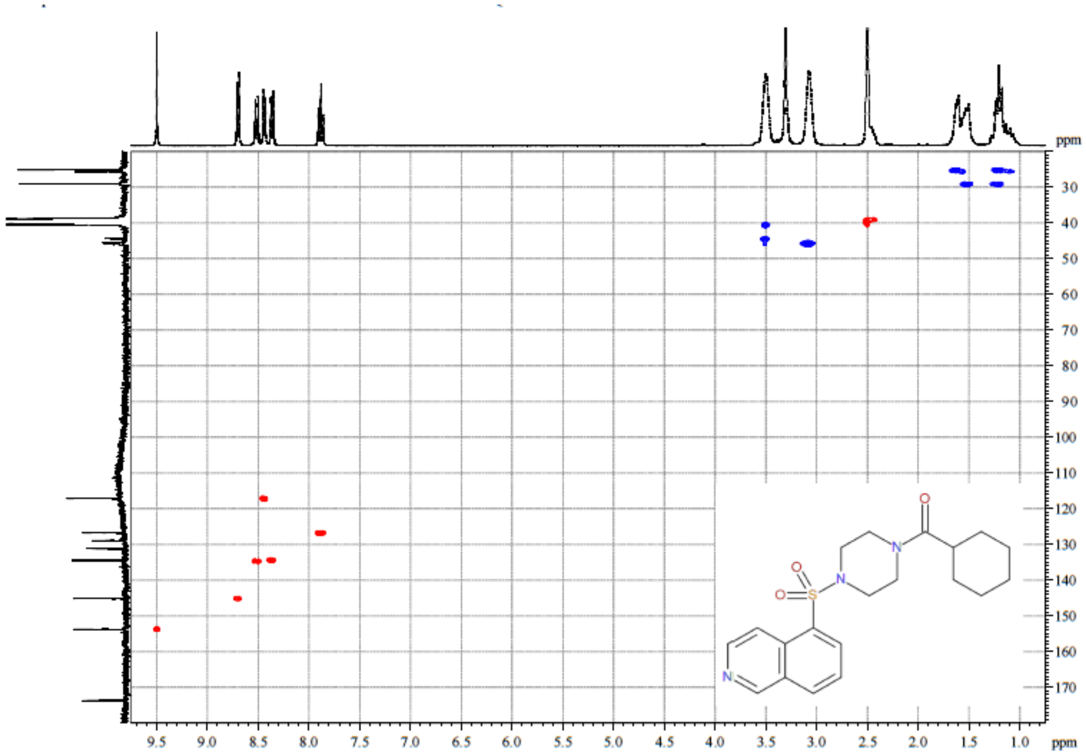

HMBC:

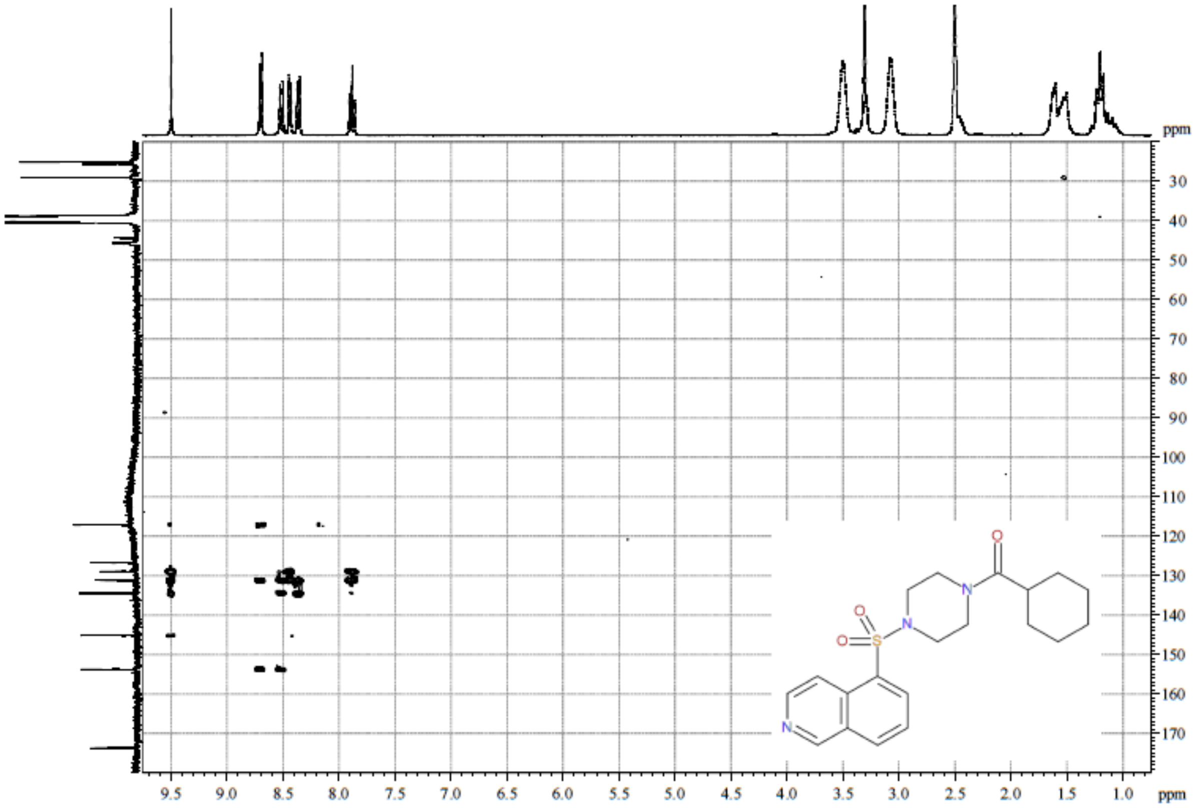

ppm
30
40
50
60
70
80
90
100
110
120
130
140
150
160
170
9.5
9.0
8.5
8.0
7.5
7.0
6.5
6.0
5.5
5.0
4.5
4.0
3.5
3.0
2.5
2.0
1.5
1.0
ppm

LC-MS:

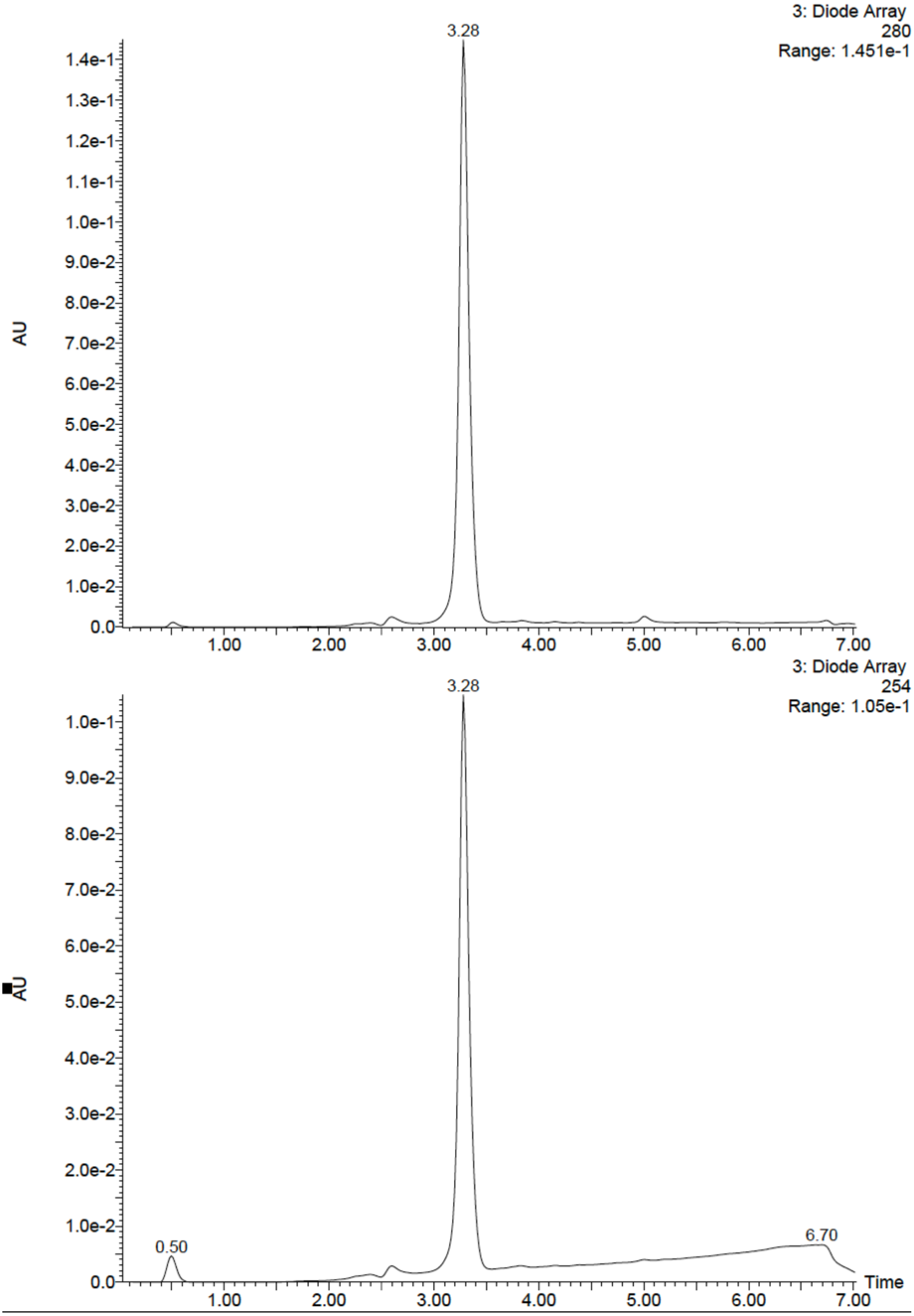

3: Diode Array
280
Range: 1.451e-1
3.28
AU
1.4e-1
1.3e-1
1.2e-1
1.1e-1
1.0e-1
9.0e-2
8.0e-2
7.0e-2
6.0e-2
5.0e-2
4.0e-2
3.0e-2
2.0e-2
1.0e-2
0.0
1.00
2.00
3.00
4.00
5.00
6.00
7.00
3: Diode Array
254
Range: 1.05e-1
3.28
0.50
6.70
AU
Time

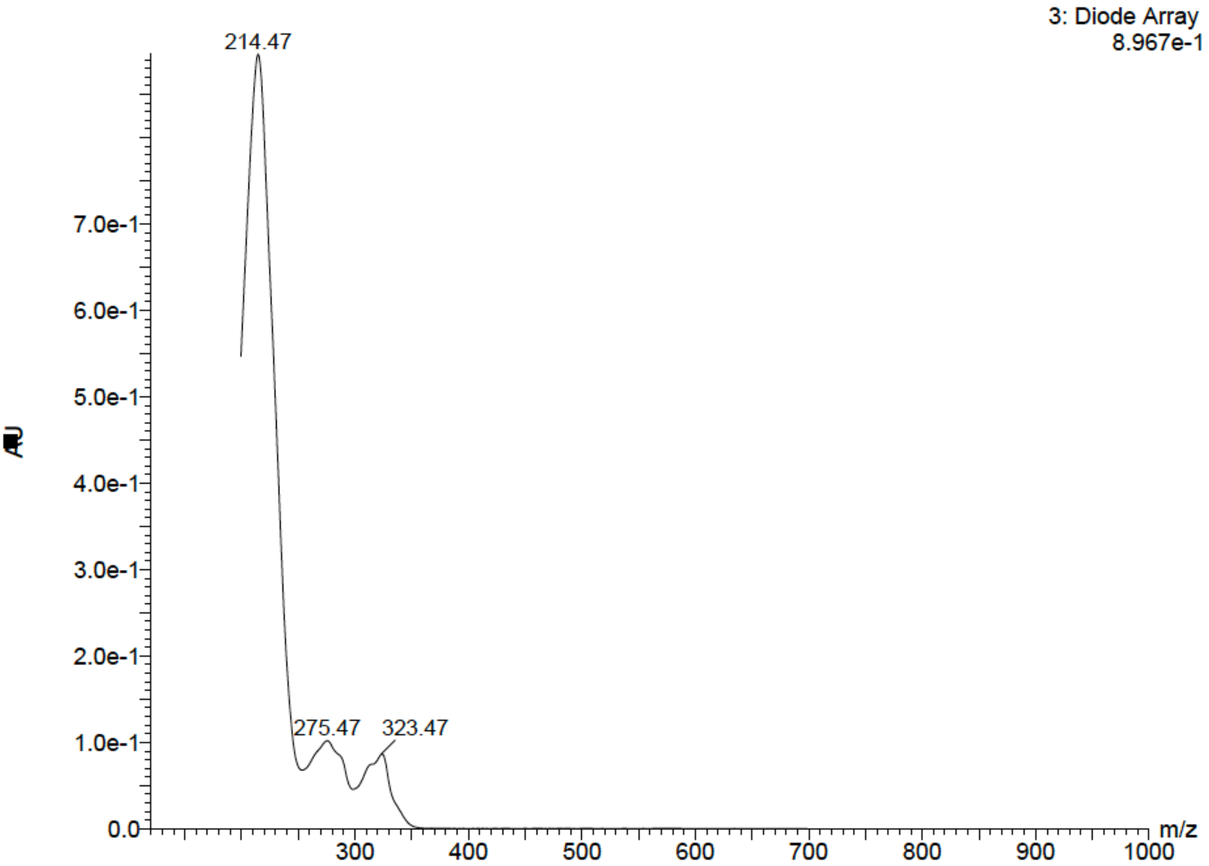
3: Diode Array
8.967e-1
214.47
275.47
323.47
AU
7.0e-1
6.0e-1
5.0e-1
4.0e-1
3.0e-1
2.0e-1
1.0e-1
0.0
300
400
500
600
700
800
900
1000
m/z

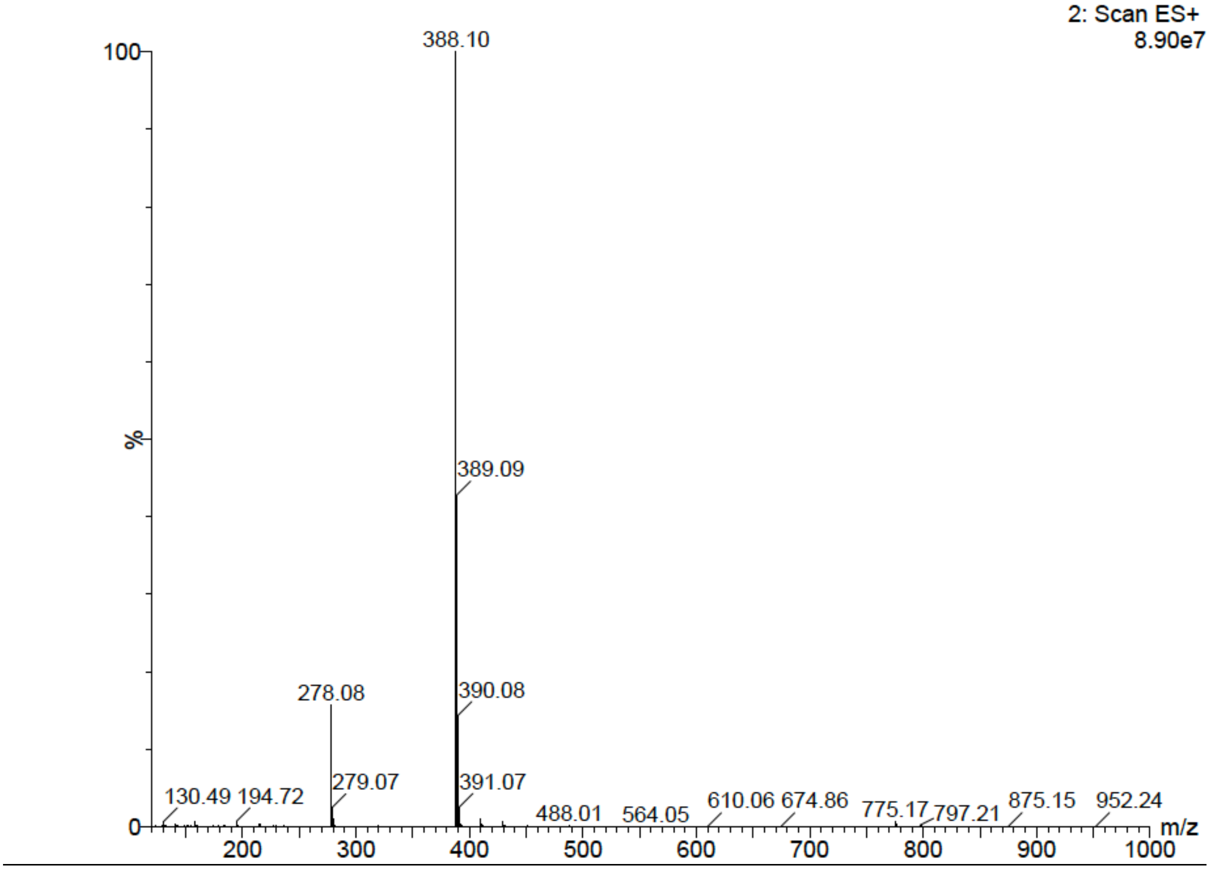
2: Scan ES+
8.90e7
388.10
389.09
390.08
391.07
278.08
279.07
130.49
194.72
488.01
564.05
610.06
674.86
775.17
797.21
875.15
952.24
100
%
0
200
300
400
500
600
700
800
900
1000
m/z

Compound 4

^{1}H-NMR chemical shifts δ [ppm]

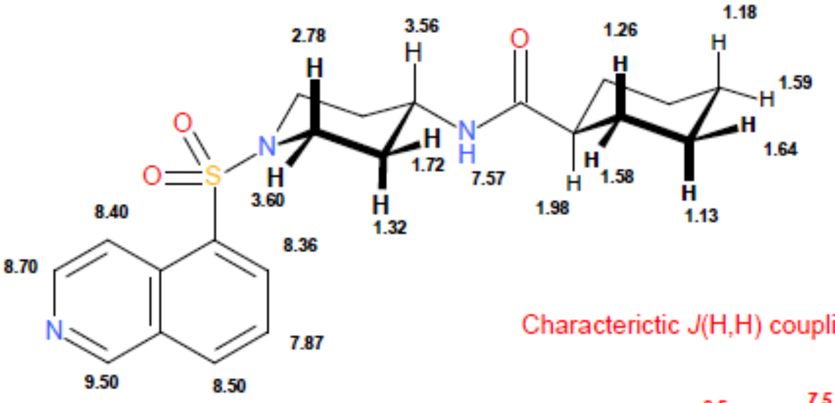

Characterictic J(H,H) coupling constants [Hz]

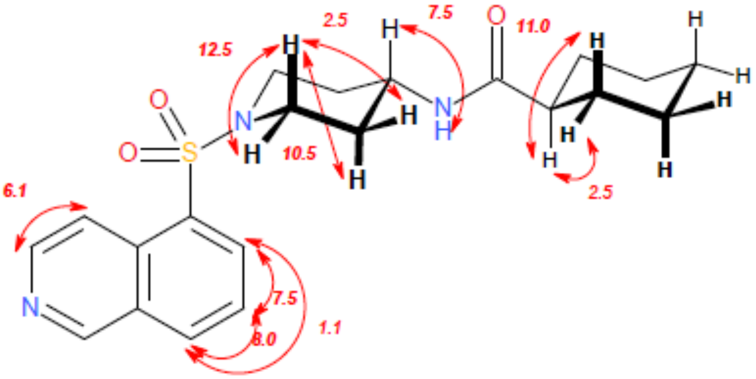

^{13}C-NMR chemical shifts δ [ppm]

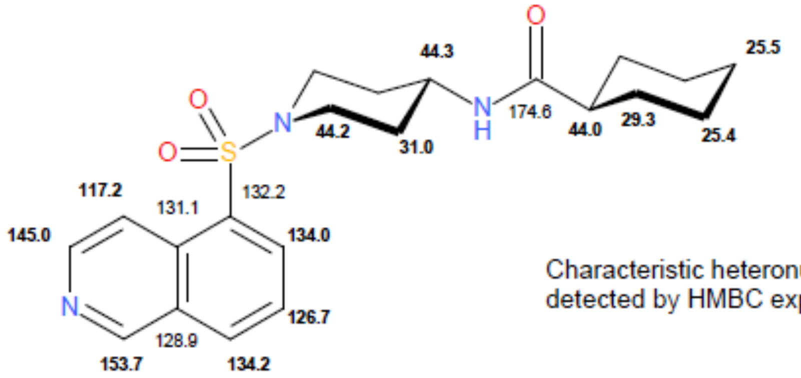

Characteristic heteronuclear long-range couplings detected by HMBC experiment H → C

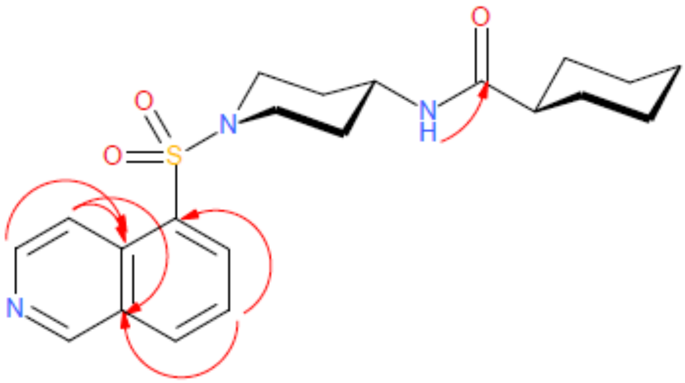

^{1}H NMR:

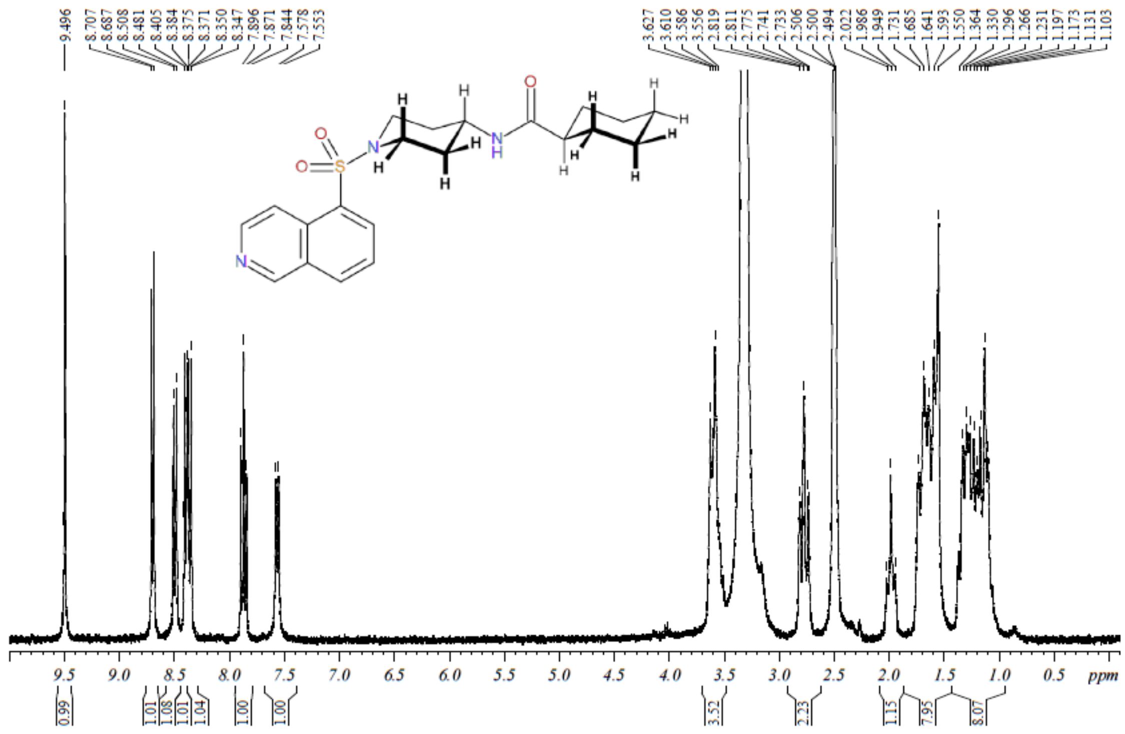

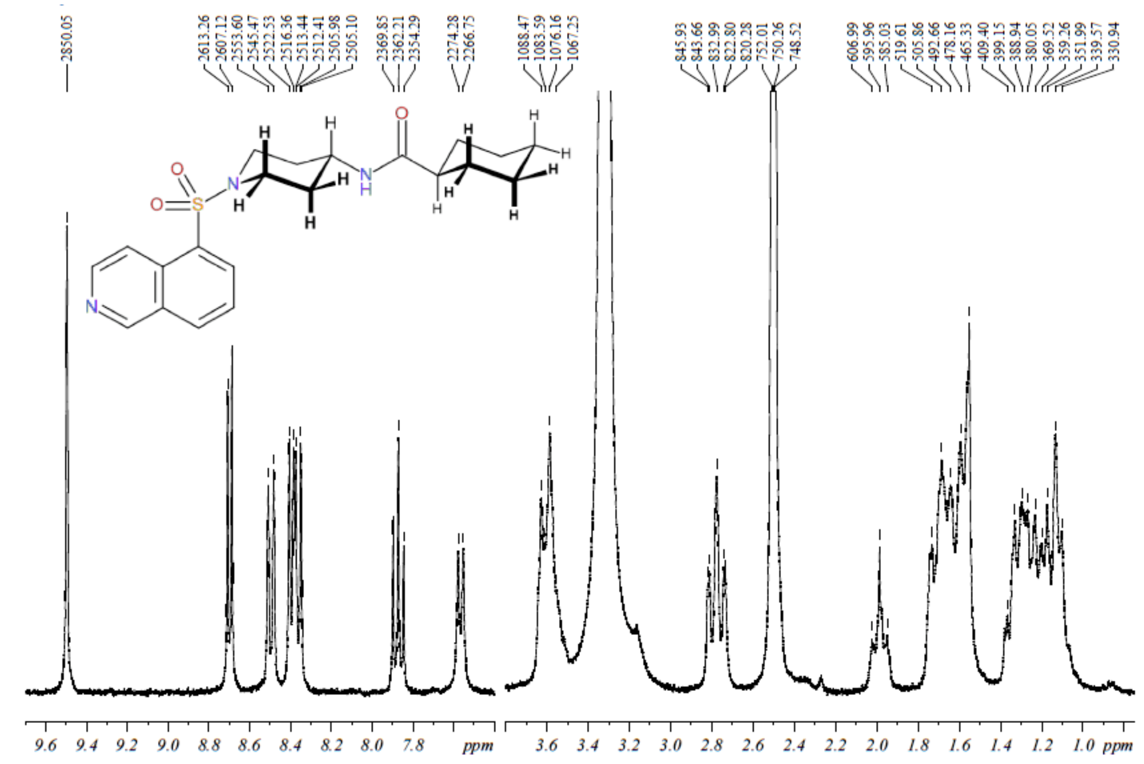

Total correlation spectroscopy (TOCSY):

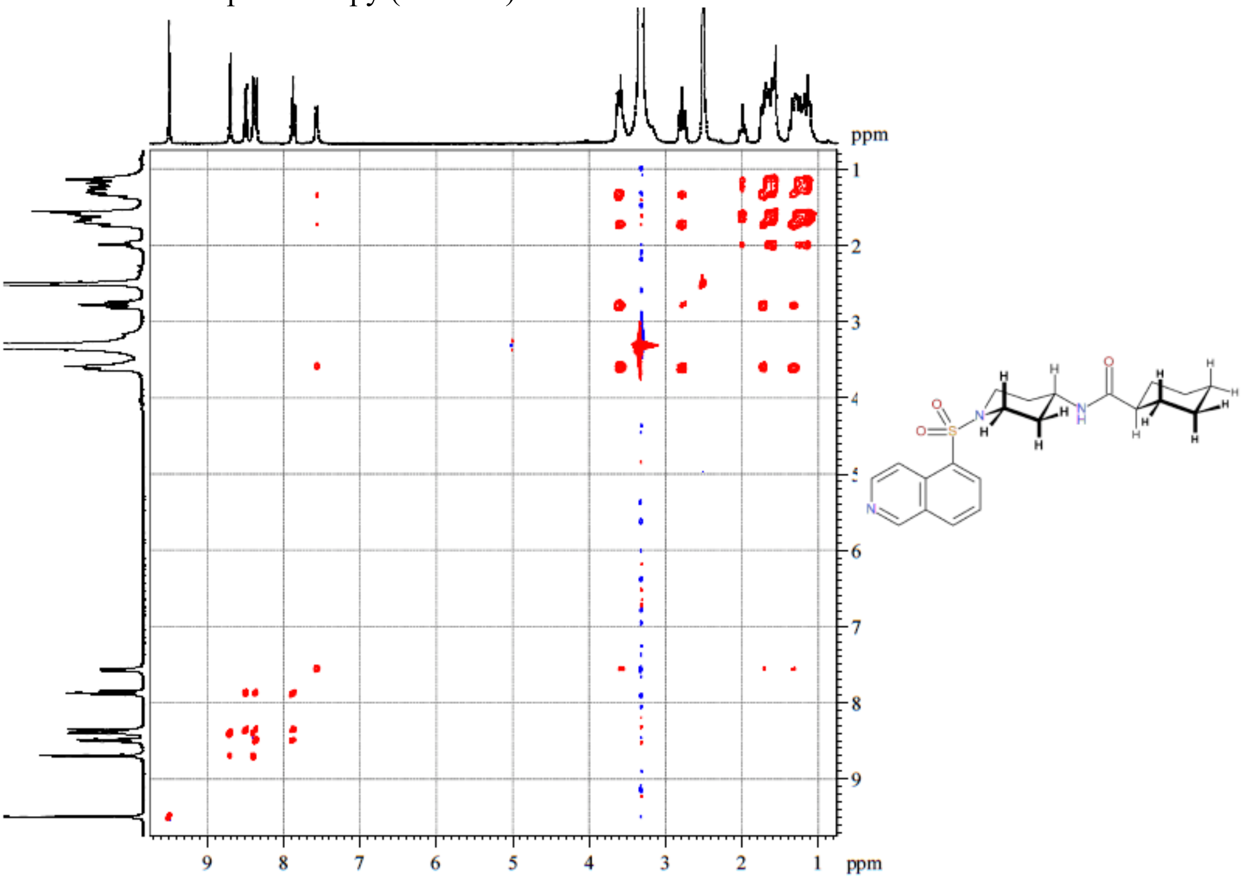

^{13}C-NMR:

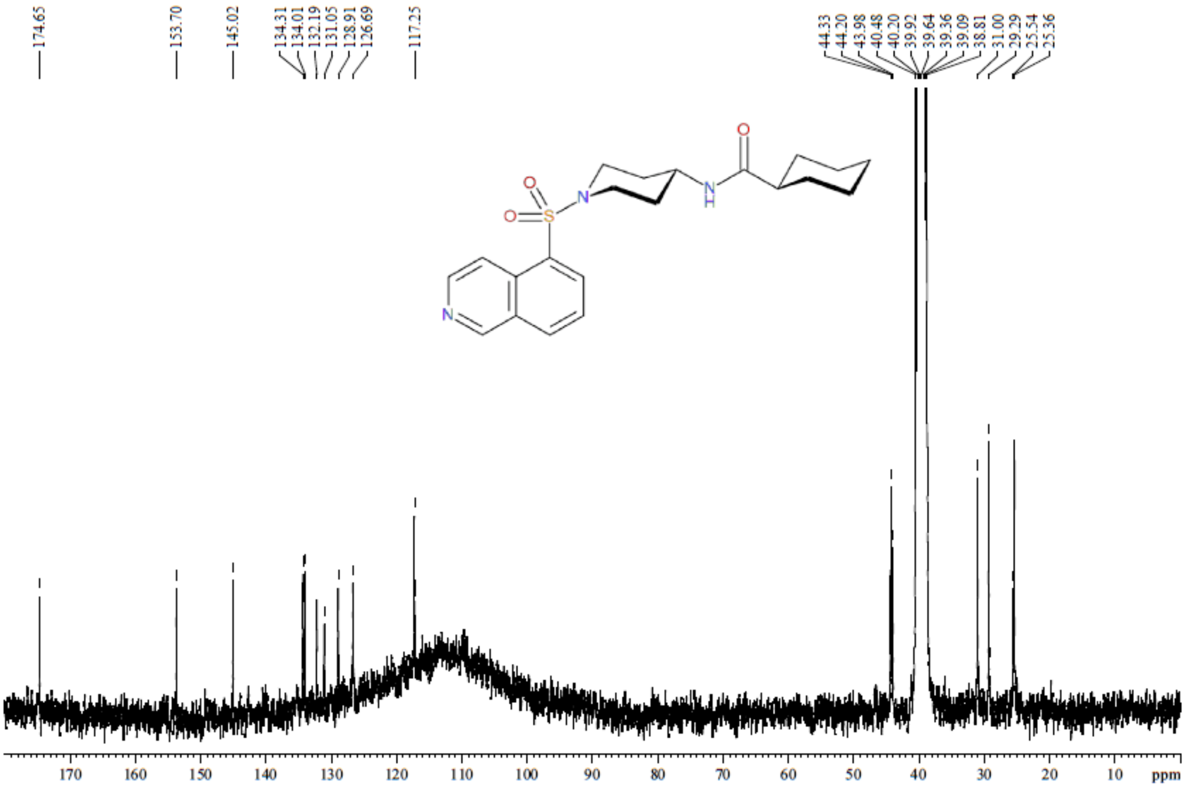

Heteronuclear single quantum coherence (HSQC):

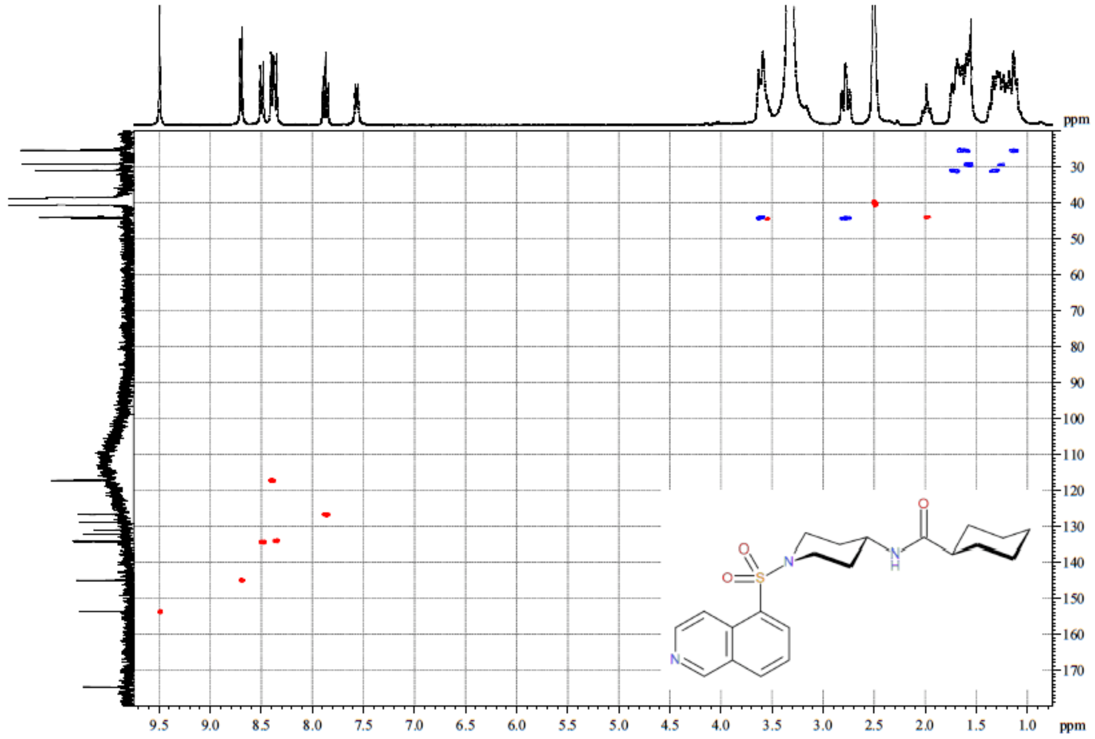

HMBC:

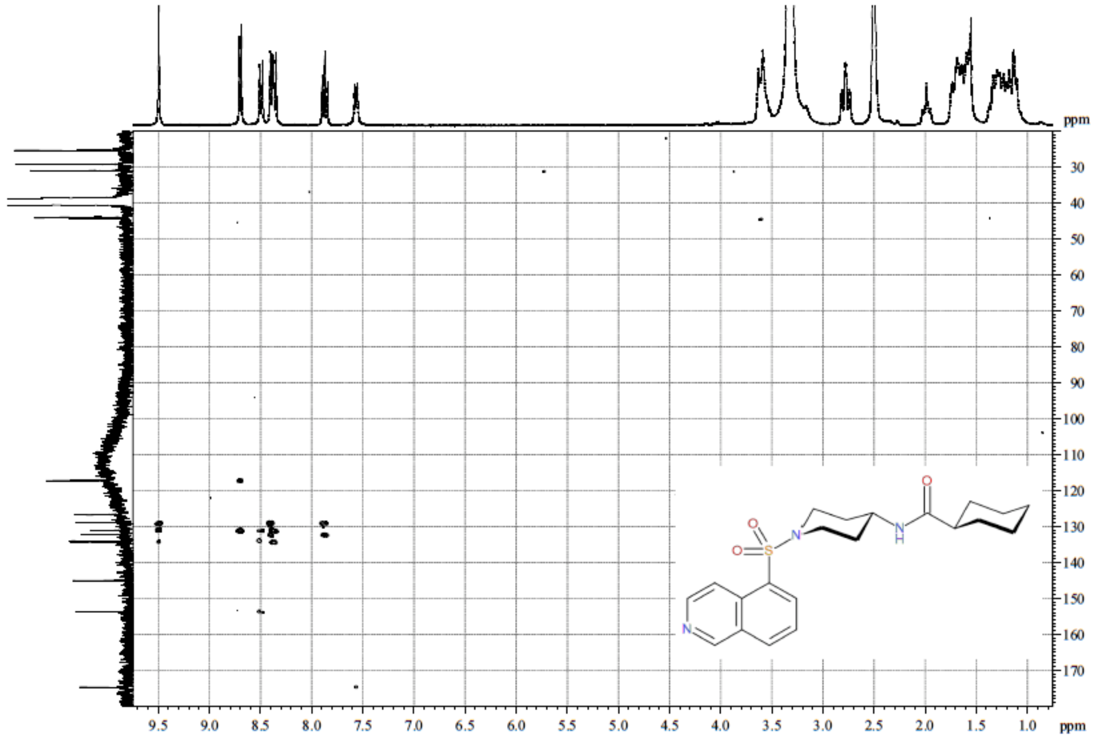

*Compound **21***

Formula Weight: 445,406; Exact Mass: 445,06921856; Molecular Formula: $C_{18}H_{15}N_5O_7S$

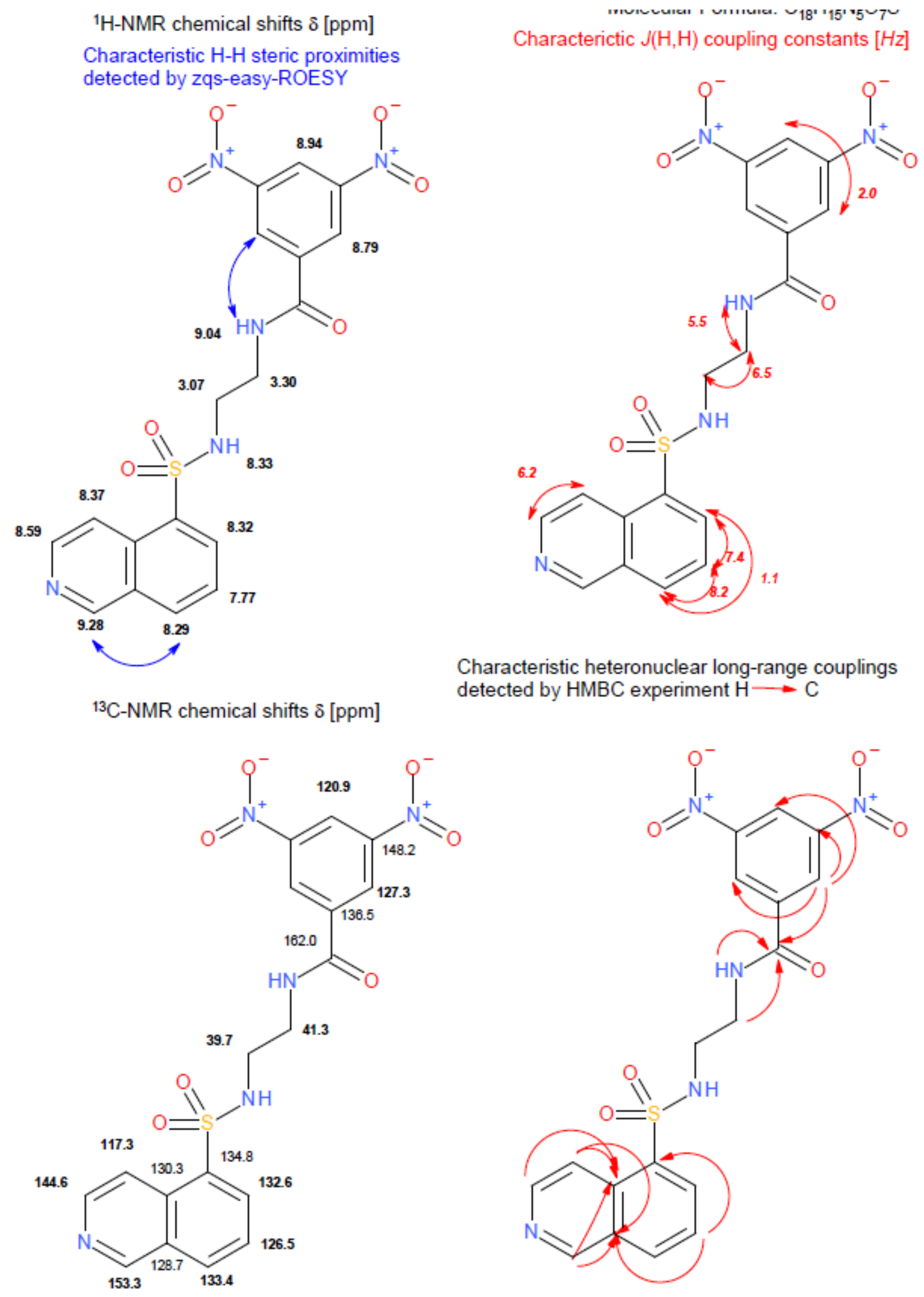

^{1}H NMR:

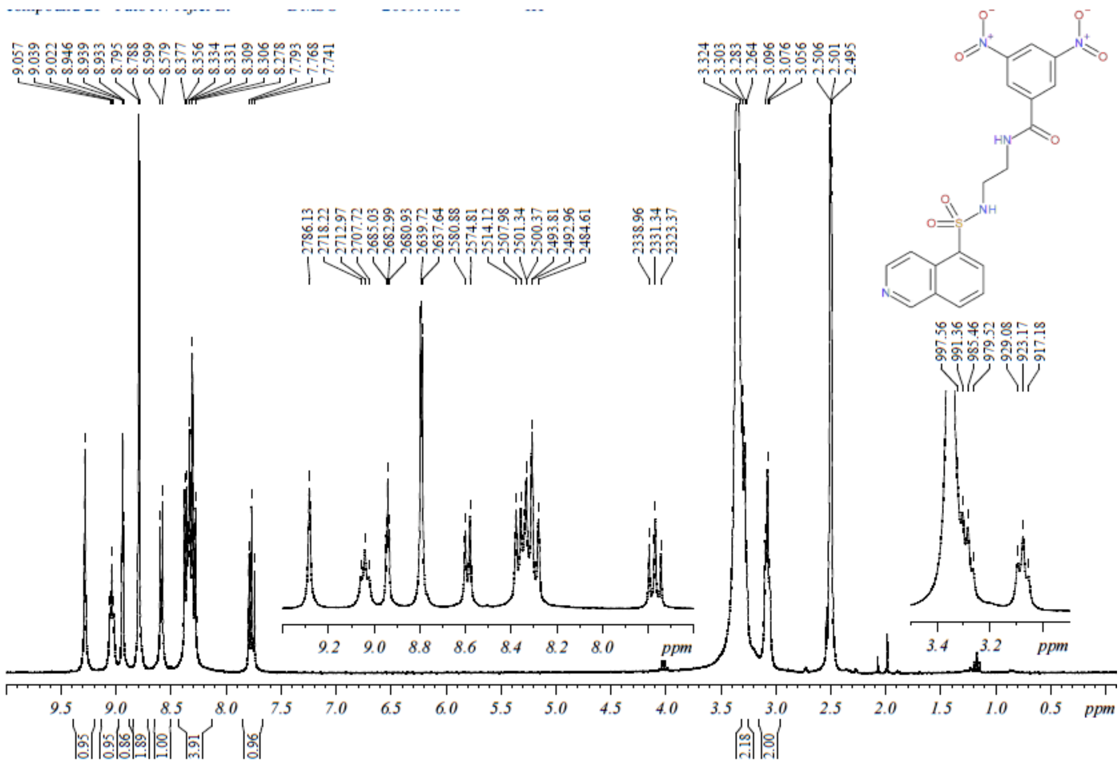

Total correlation spectroscopy (TOCSY):

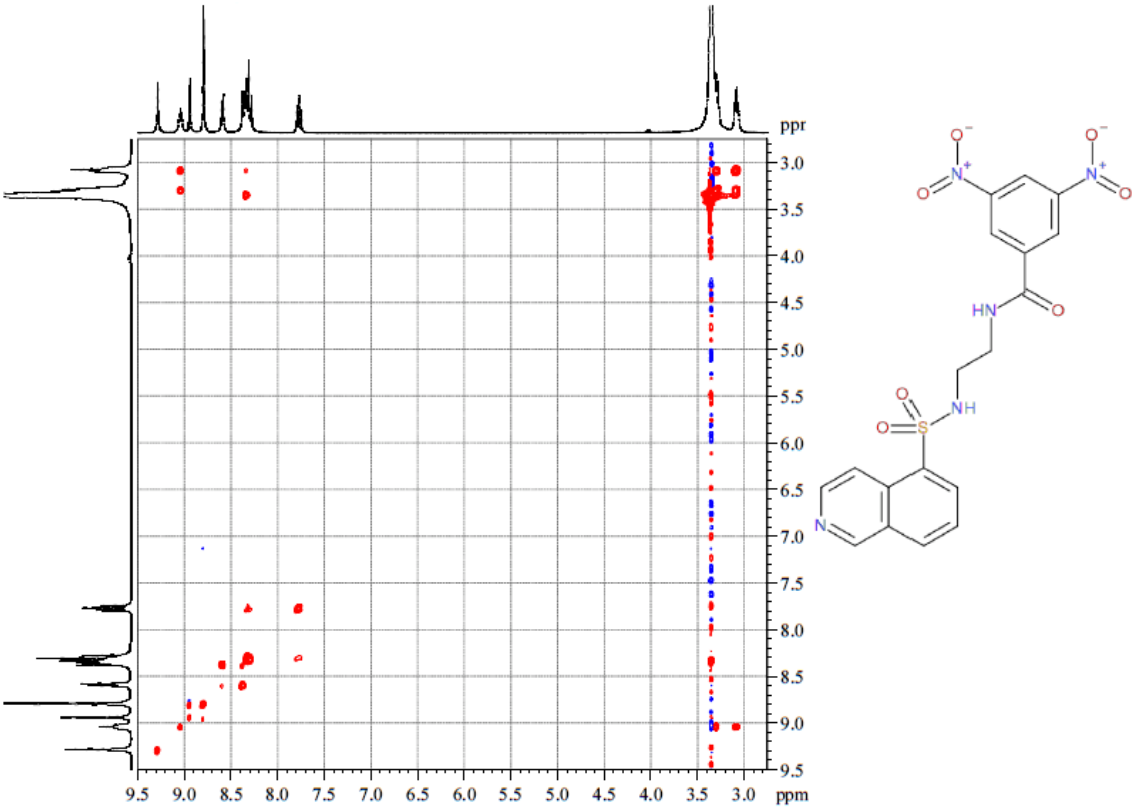

ROSEY:

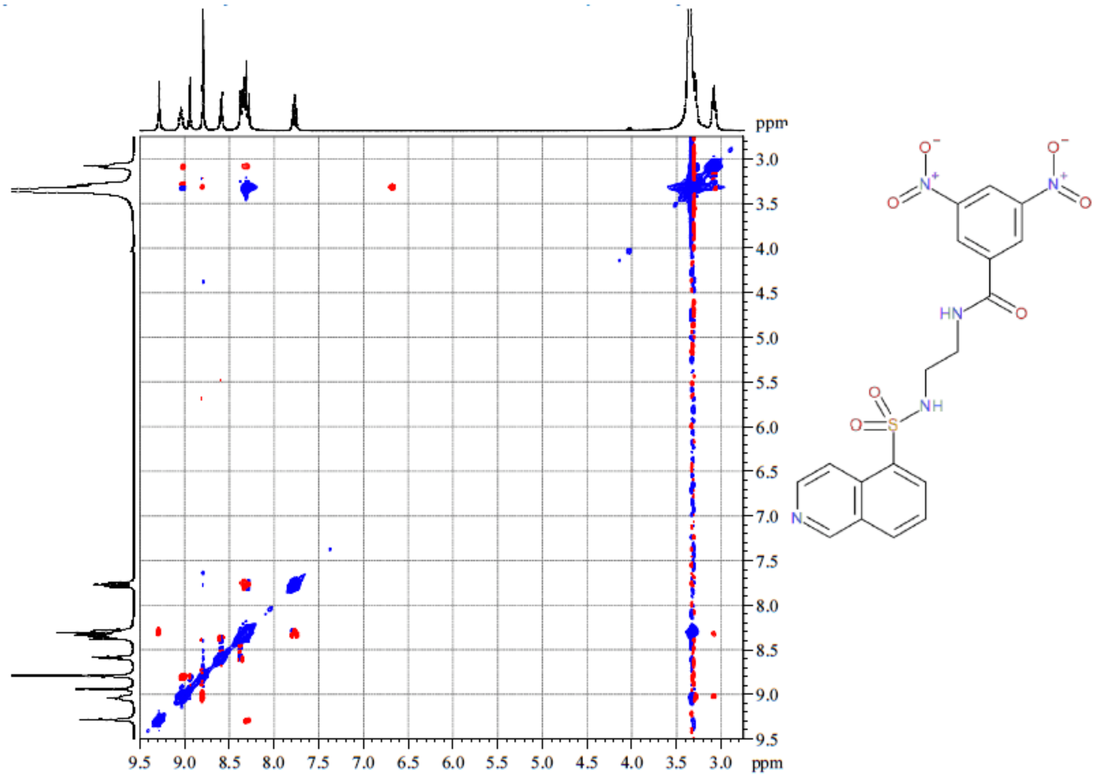

^{13}C-NMR:

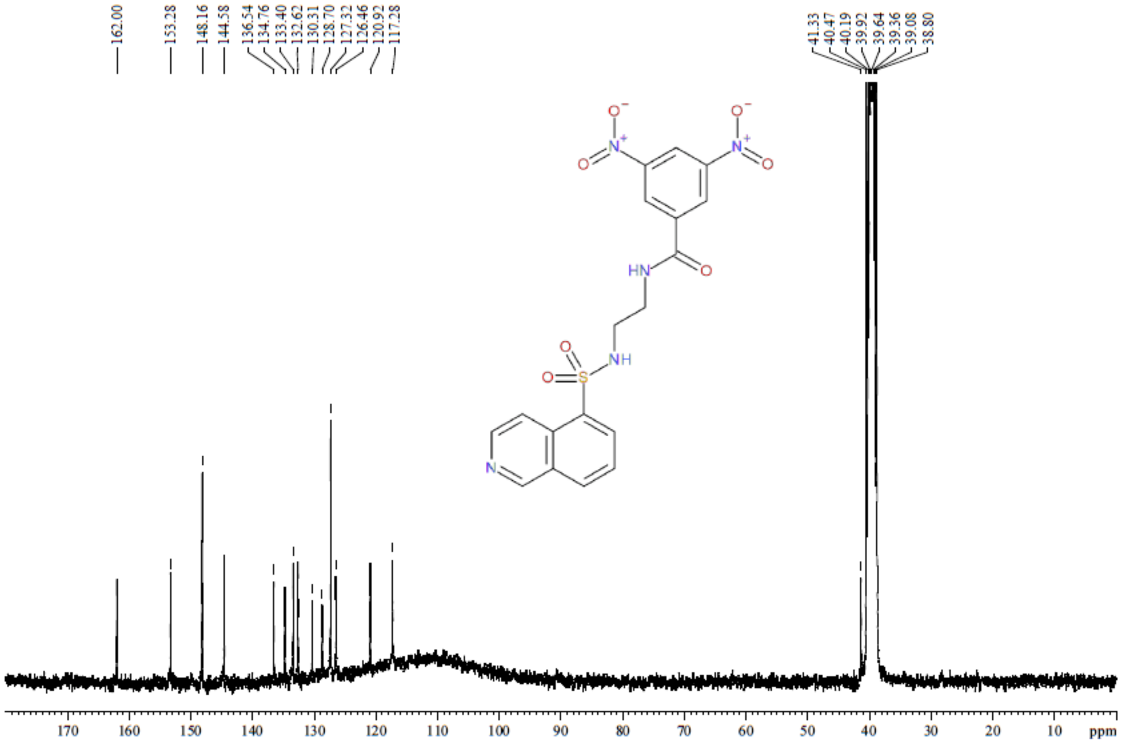

Heteronuclear single quantum coherence (HSQC):

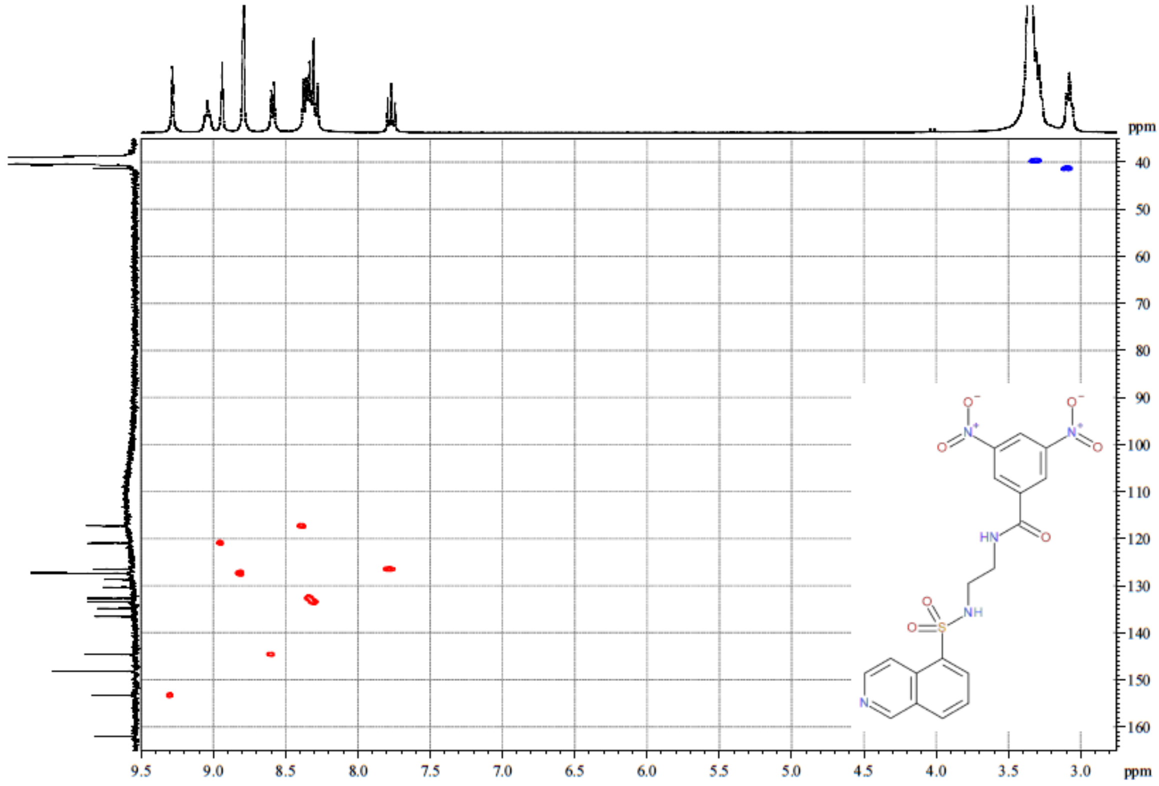

HMBC:

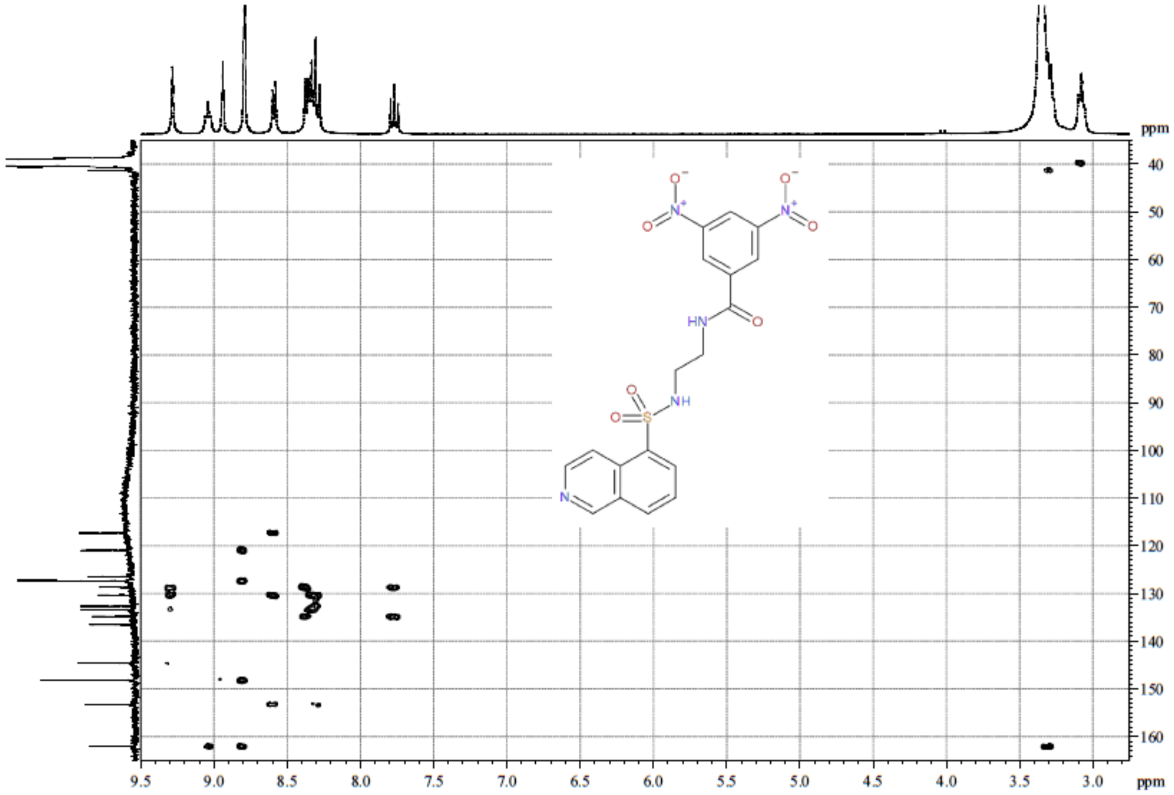

LC-MS:

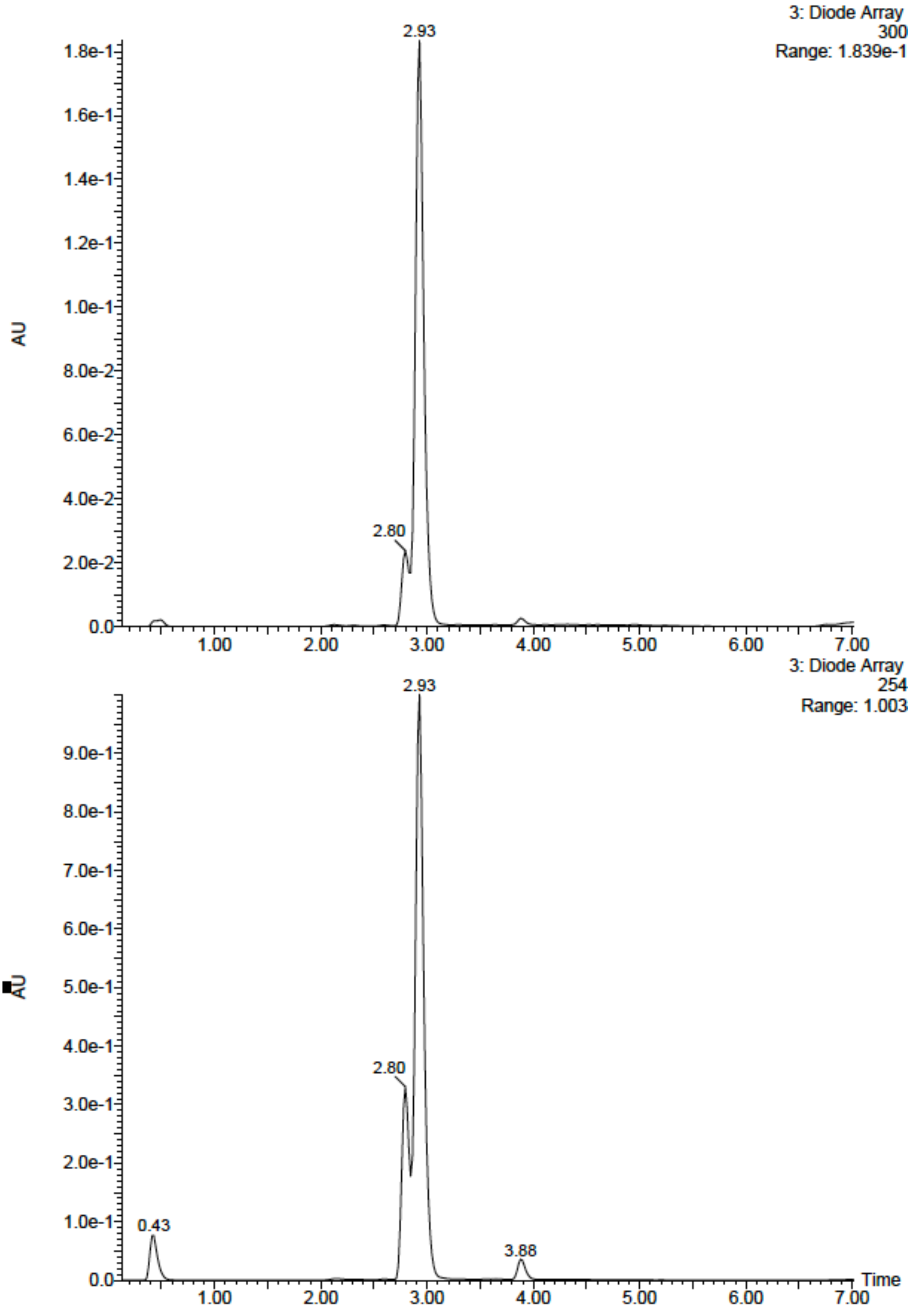

3: Diode Array
300
Range: 1.839e-1
2.93
2.80
AU
1.8e-1
1.6e-1
1.4e-1
1.2e-1
1.0e-1
8.0e-2
6.0e-2
4.0e-2
2.0e-2
0.0
1.00
2.00
3.00
4.00
5.00
6.00
7.00
3: Diode Array
254
Range: 1.003
2.93
2.80
0.43
3.88
AU
9.0e-1
8.0e-1
7.0e-1
6.0e-1
5.0e-1
4.0e-1
3.0e-1
2.0e-1
1.0e-1
0.0
Time
1.00
2.00
3.00
4.00
5.00
6.00
7.00

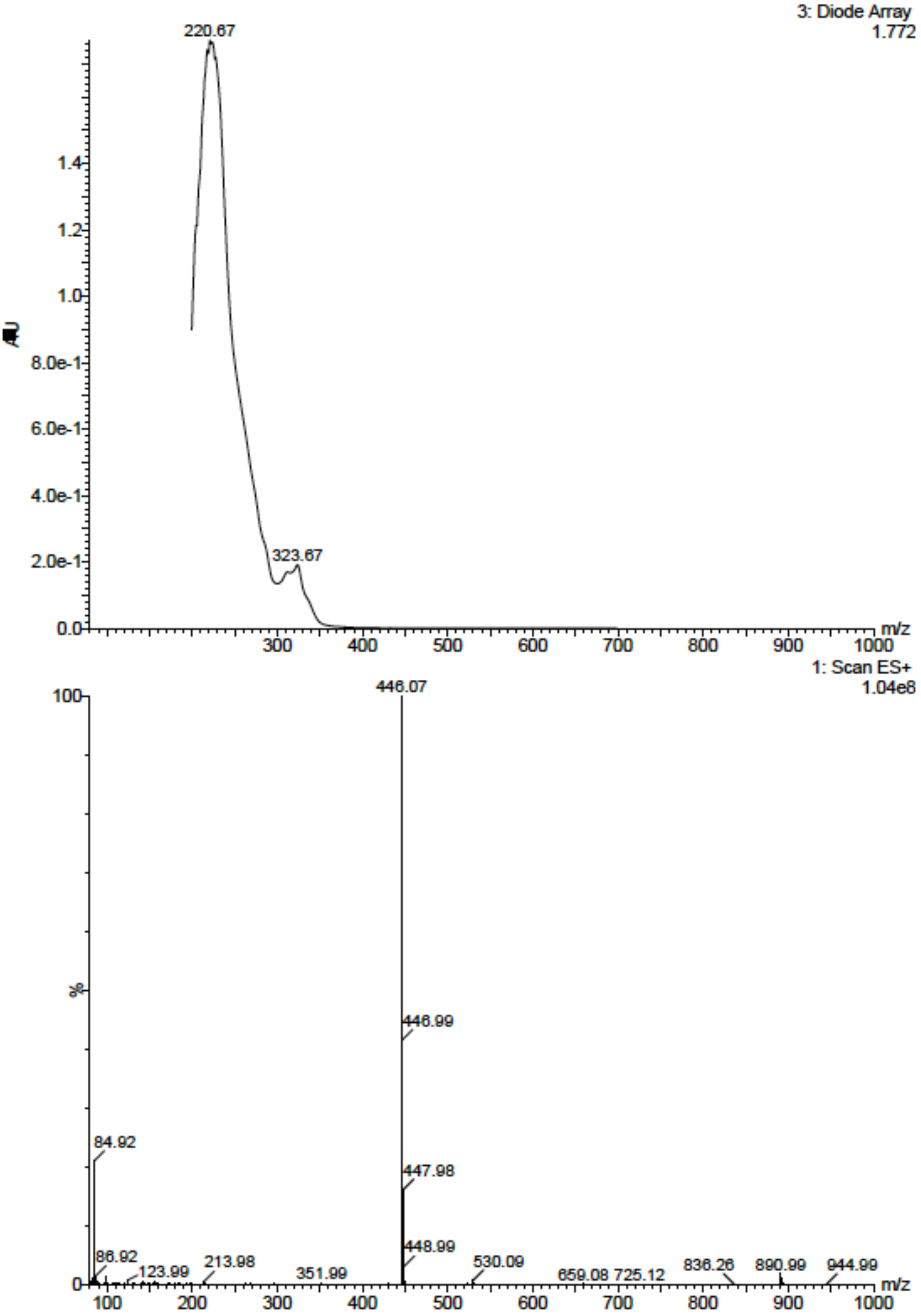
3: Diode Array
1.772
220.67
323.67
AU
m/z
1: Scan ES+
1.04e8
446.07
446.99
447.98
448.99
84.92
86.92
123.99
213.98
351.99
530.09
659.08 725.12
836.26
890.99
944.99
%
m/z

*Compound **46***

Formula Weight: 410,48938; Exact Mass: 410,141261294; Molecular Formula: $C_{21}H_{22}N_4O_3S$

^{1}H-NMR chemical shifts δ [ppm]

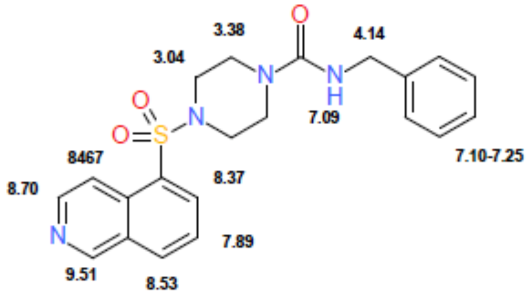

Characterictic J(H,H) coupling constants [Hz]

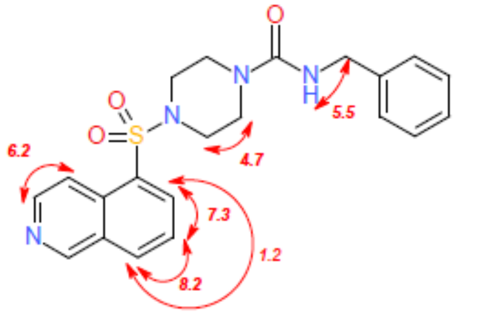

^{13}C-NMR chemical shifts δ [ppm]

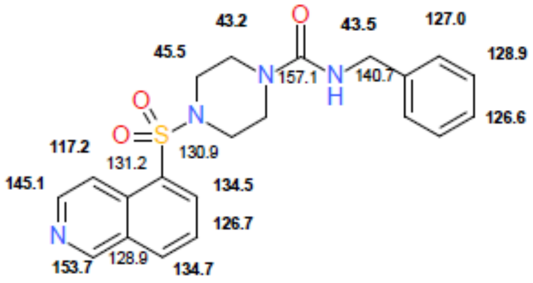

Characteristic heteronuclear long-range couplings detected by HMBC experiment H → C

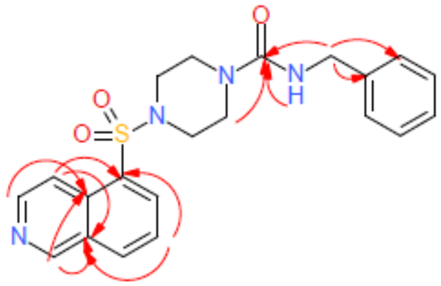

^{1}H-NMR:

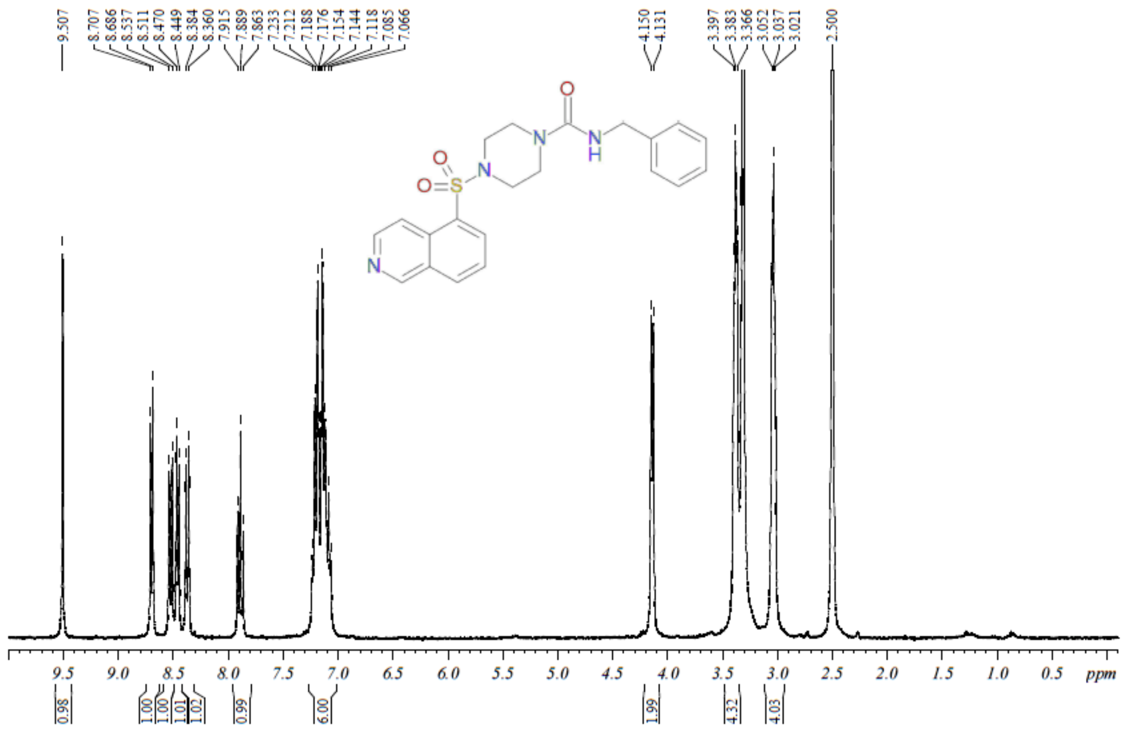

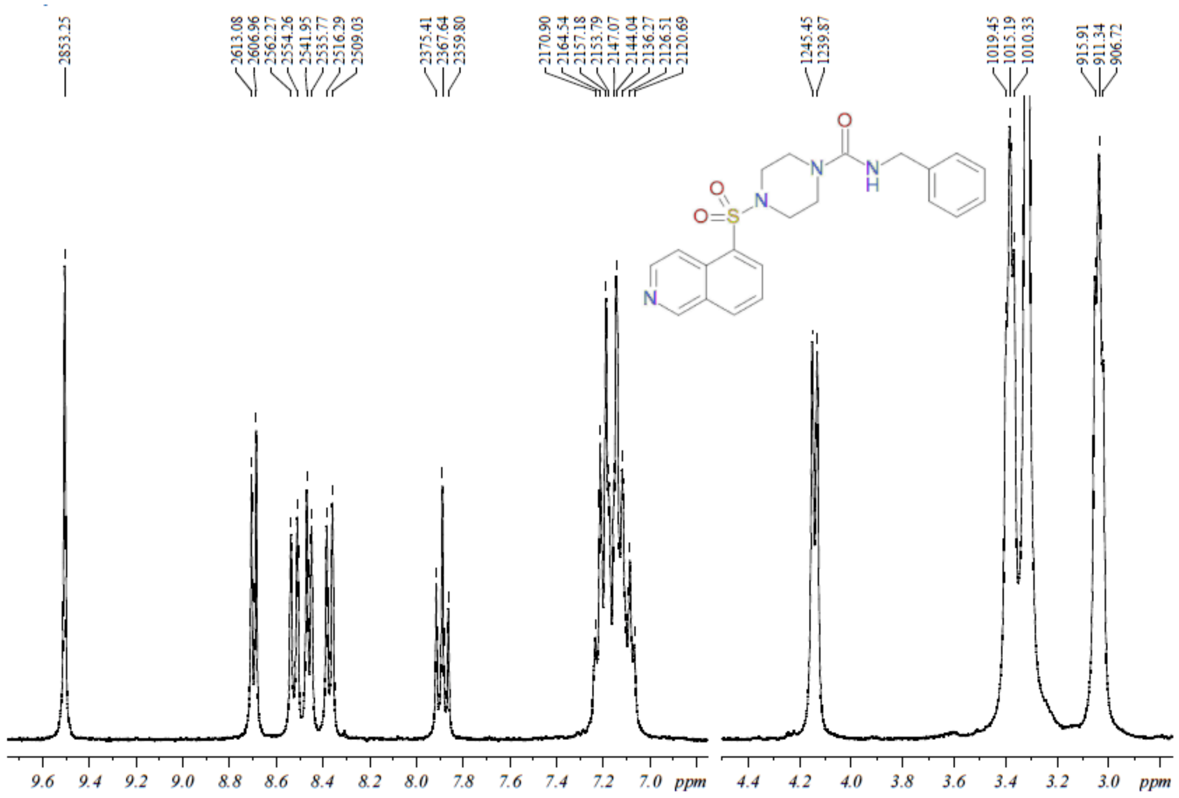

Total correlation spectroscopy (TOCSY):

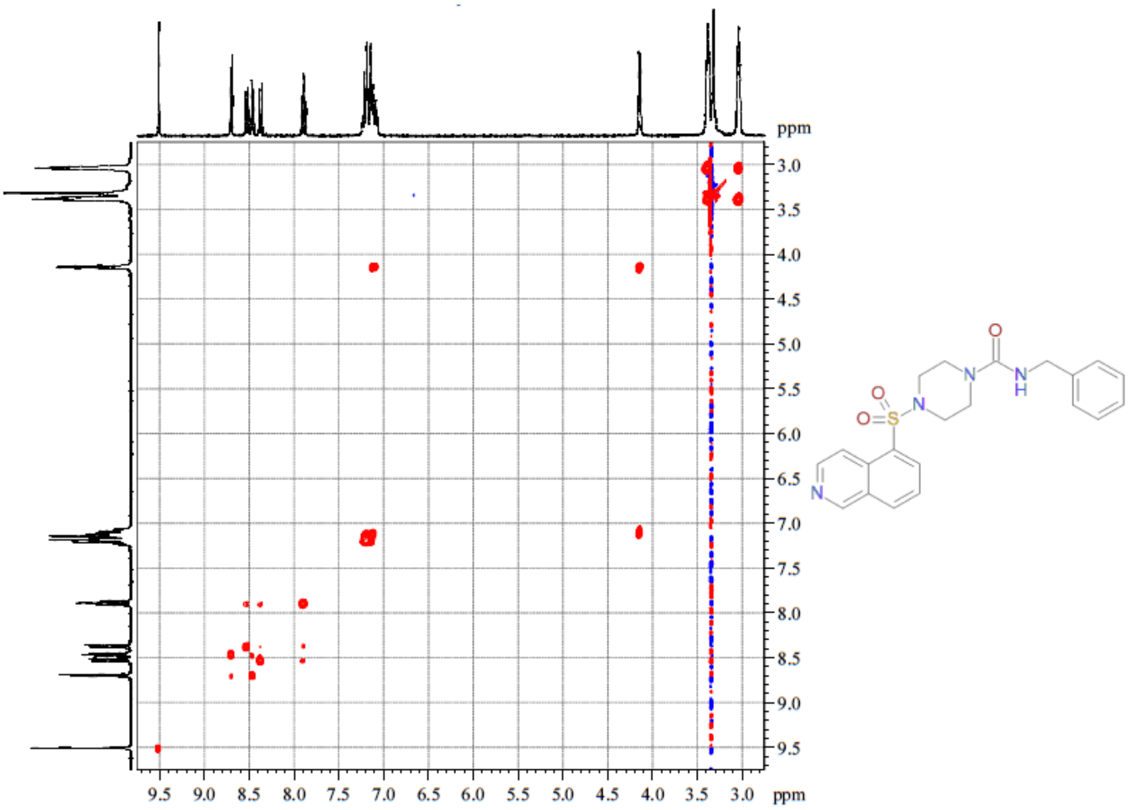

^{13}C-NMR:

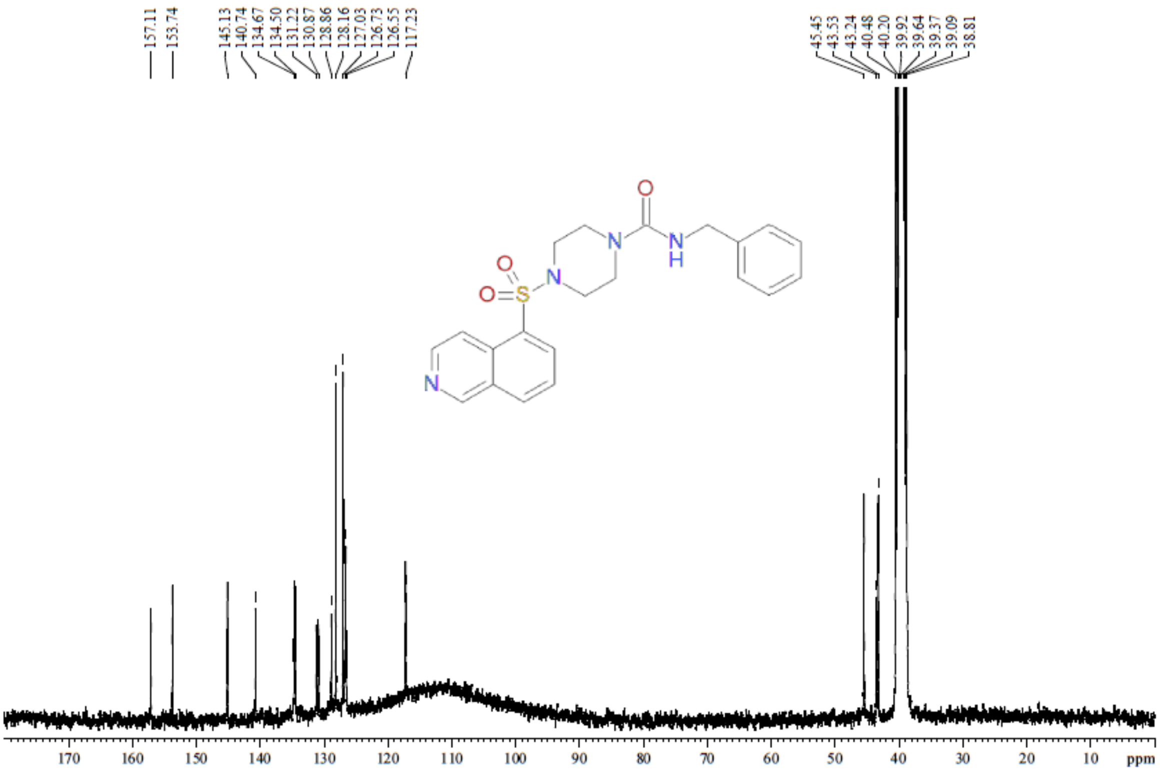

Heteronuclear single quantum coherence (HSQC):

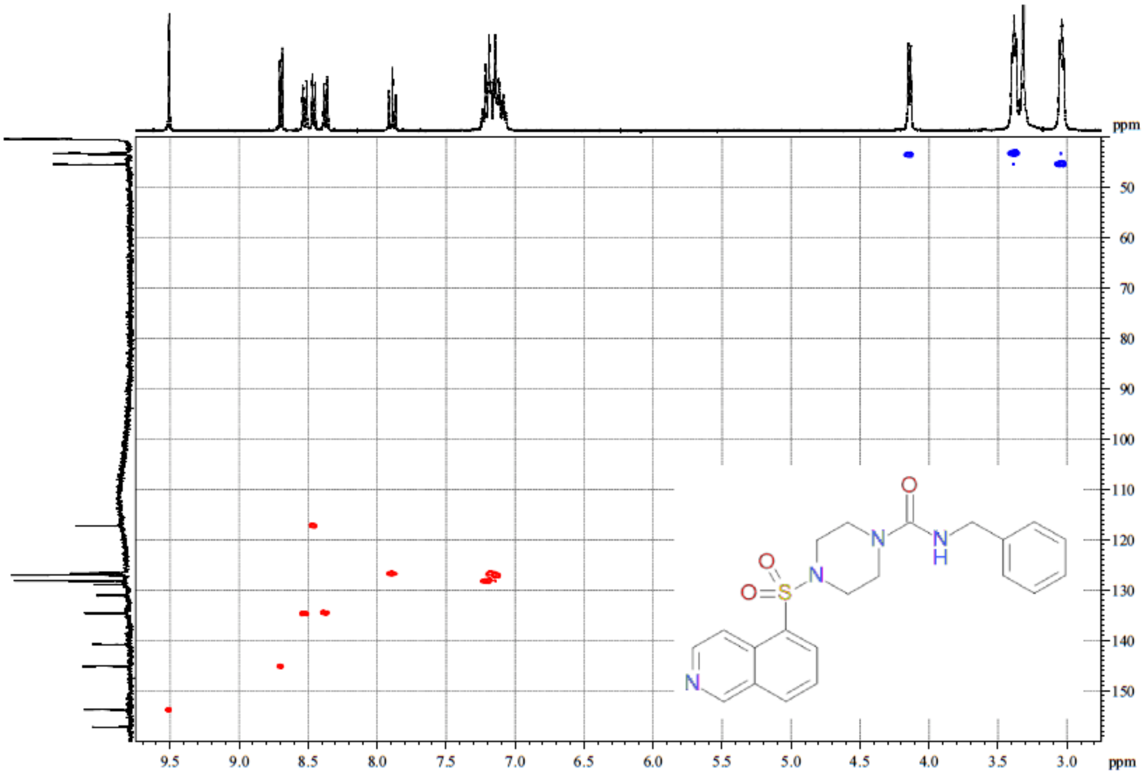

HMBC:

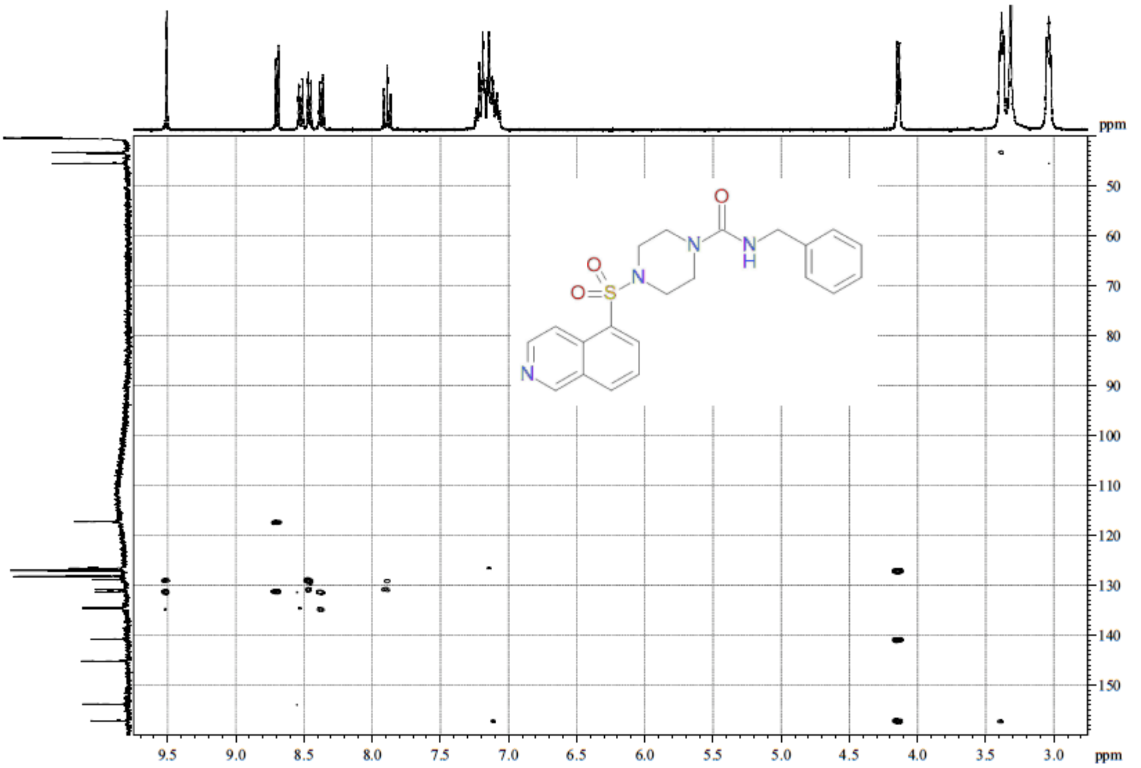

*Compound **47***

^{1}H NMR:

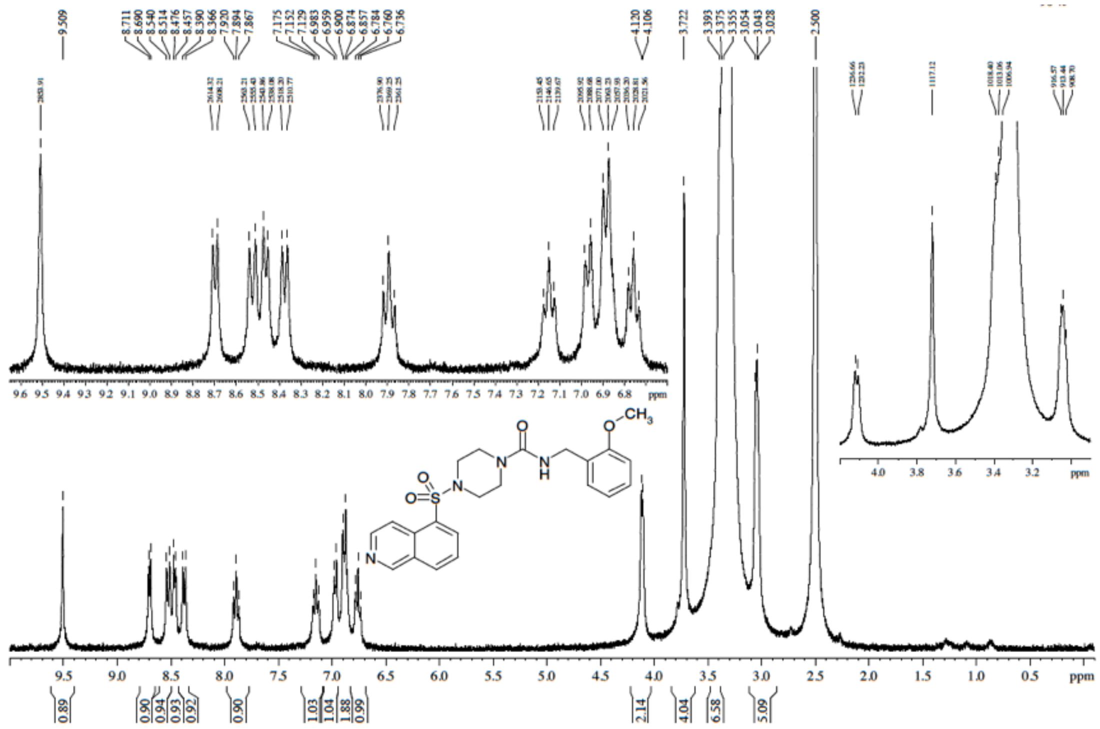

^{13}C NMR:

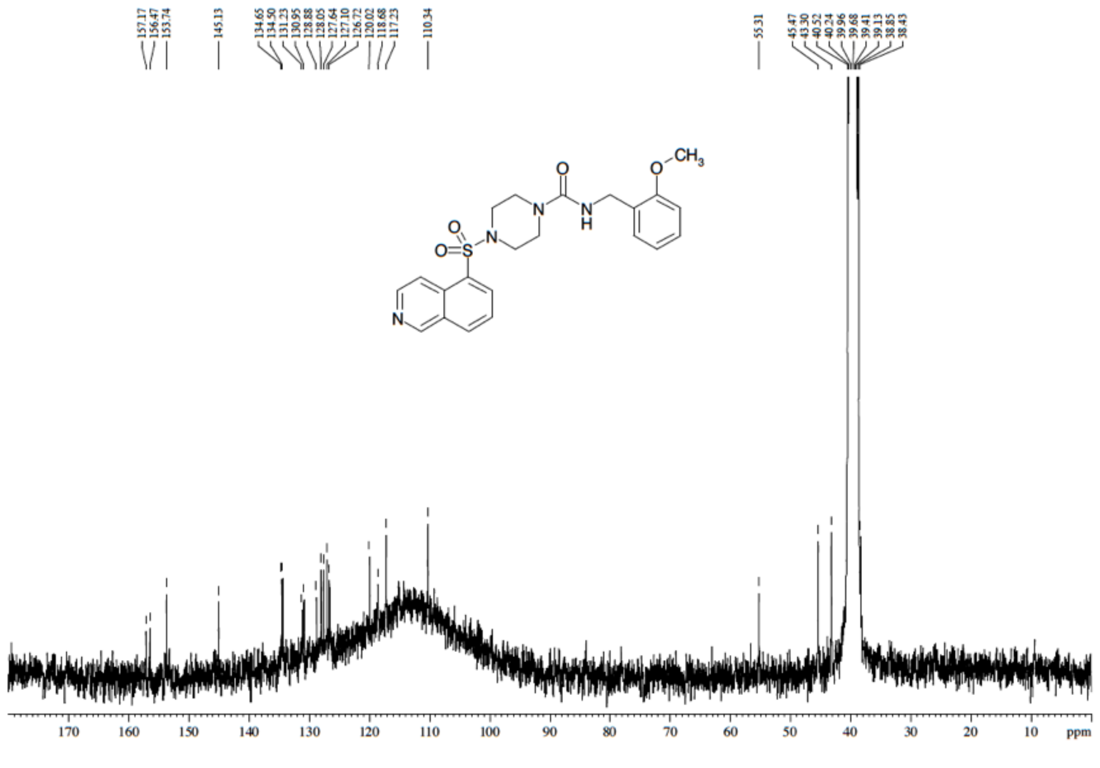

Total correlation spectroscopy (TOCSY):

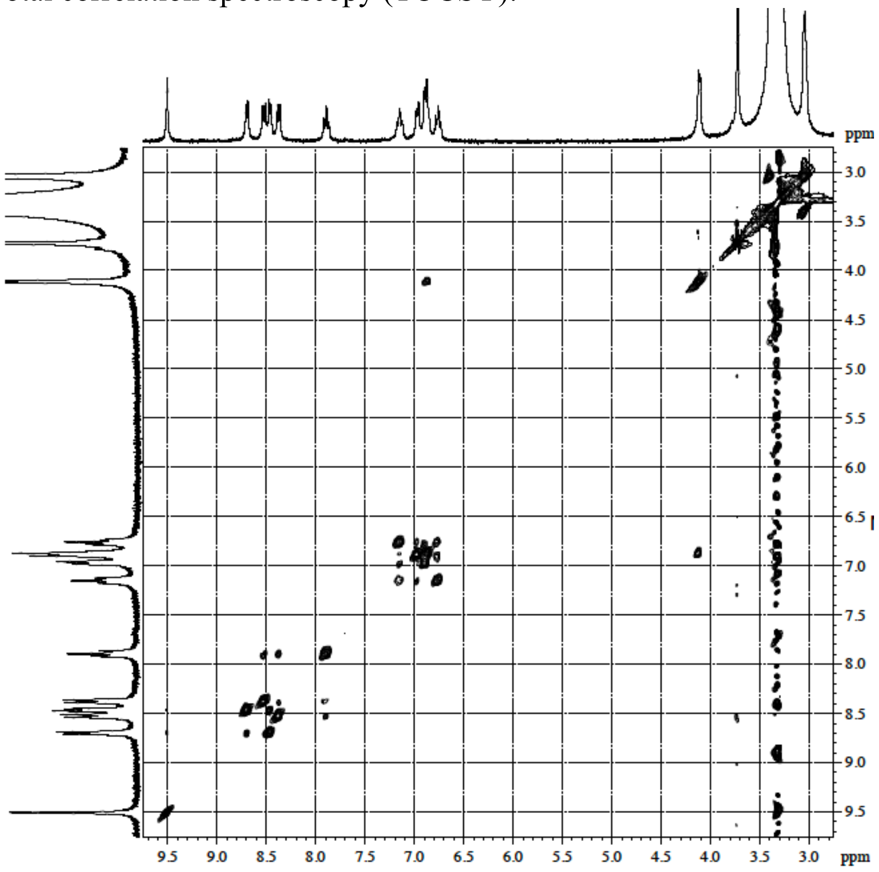

Heteronuclear single quantum coherence (HSQC):

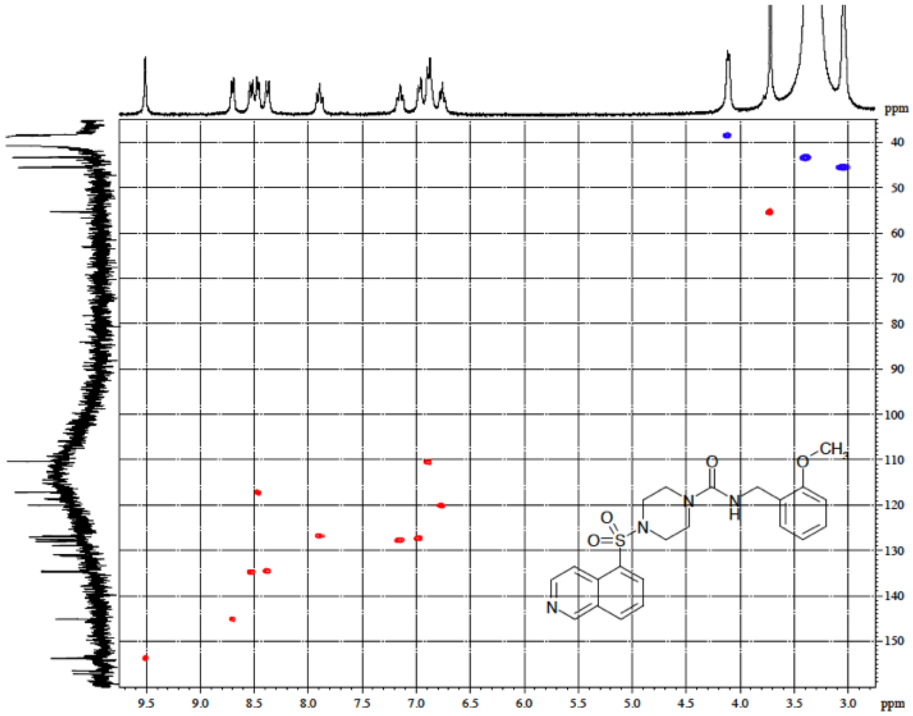

LC-MS:

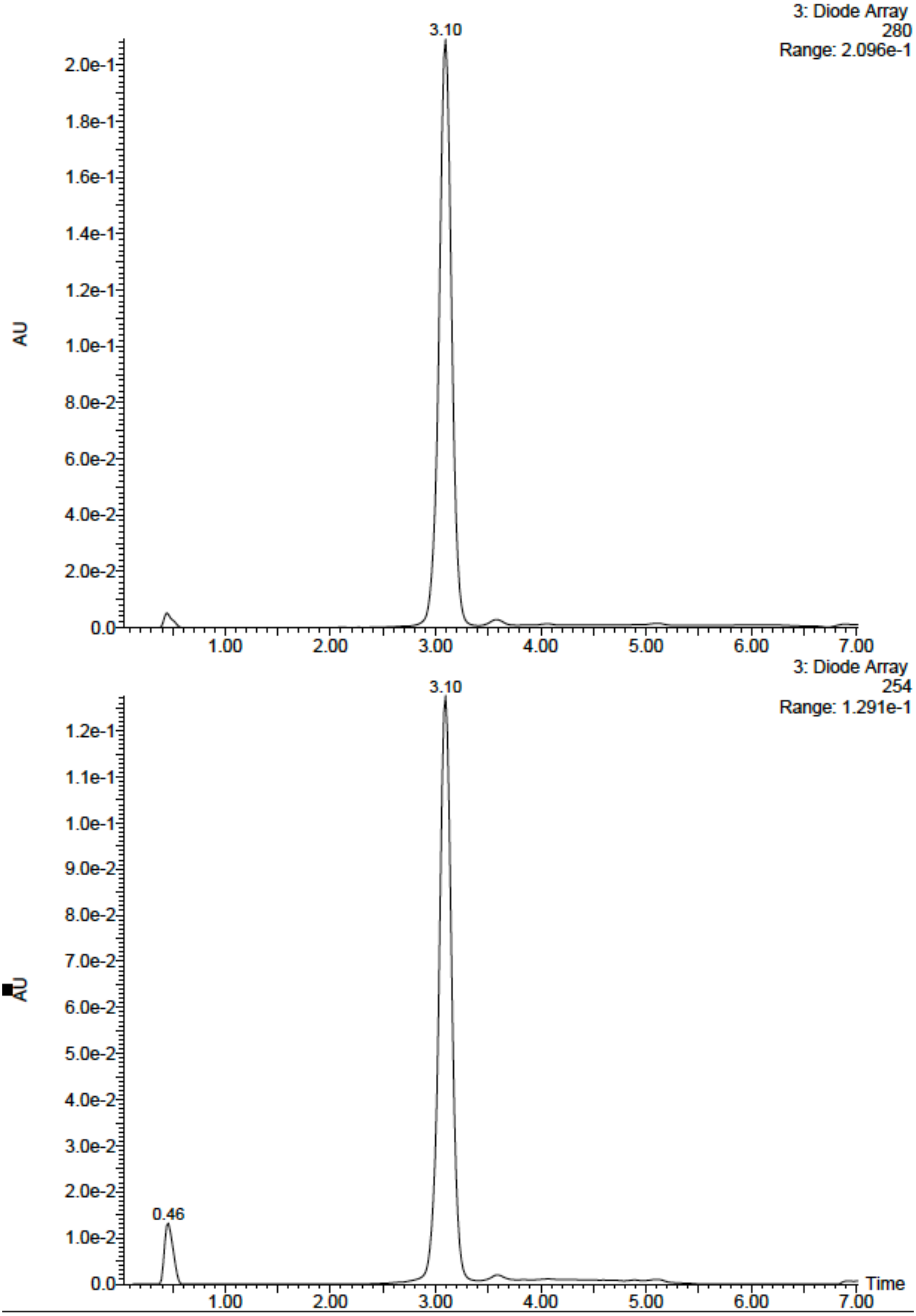
3: Diode Array
280
Range: 2.096e-1
3.10
AU
2.0e-1
1.8e-1
1.6e-1
1.4e-1
1.2e-1
1.0e-1
8.0e-2
6.0e-2
4.0e-2
2.0e-2
0.0
1.00
2.00
3.00
4.00
5.00
6.00
7.00
3: Diode Array
254
Range: 1.291e-1
3.10
0.46
AU
1.2e-1
1.1e-1
1.0e-1
9.0e-2
8.0e-2
7.0e-2
6.0e-2
5.0e-2
4.0e-2
3.0e-2
2.0e-2
1.0e-2
0.0
Time
1.00
2.00
3.00
4.00
5.00
6.00
7.00

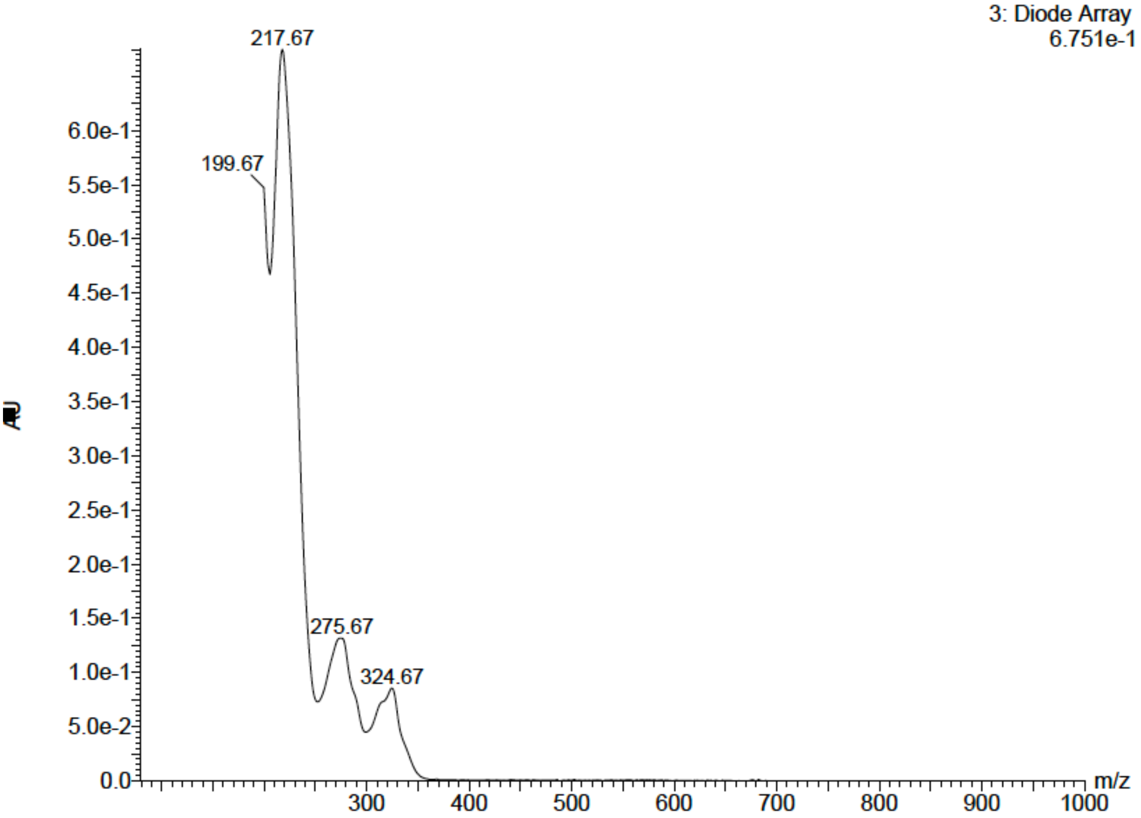

3: Diode Array
6.751e-1
217.67
199.67
275.67
324.67
AU
6.0e-1
5.5e-1
5.0e-1
4.5e-1
4.0e-1
3.5e-1
3.0e-1
2.5e-1
2.0e-1
1.5e-1
1.0e-1
5.0e-2
0.0
300
400
500
600
700
800
900
1000
m/z

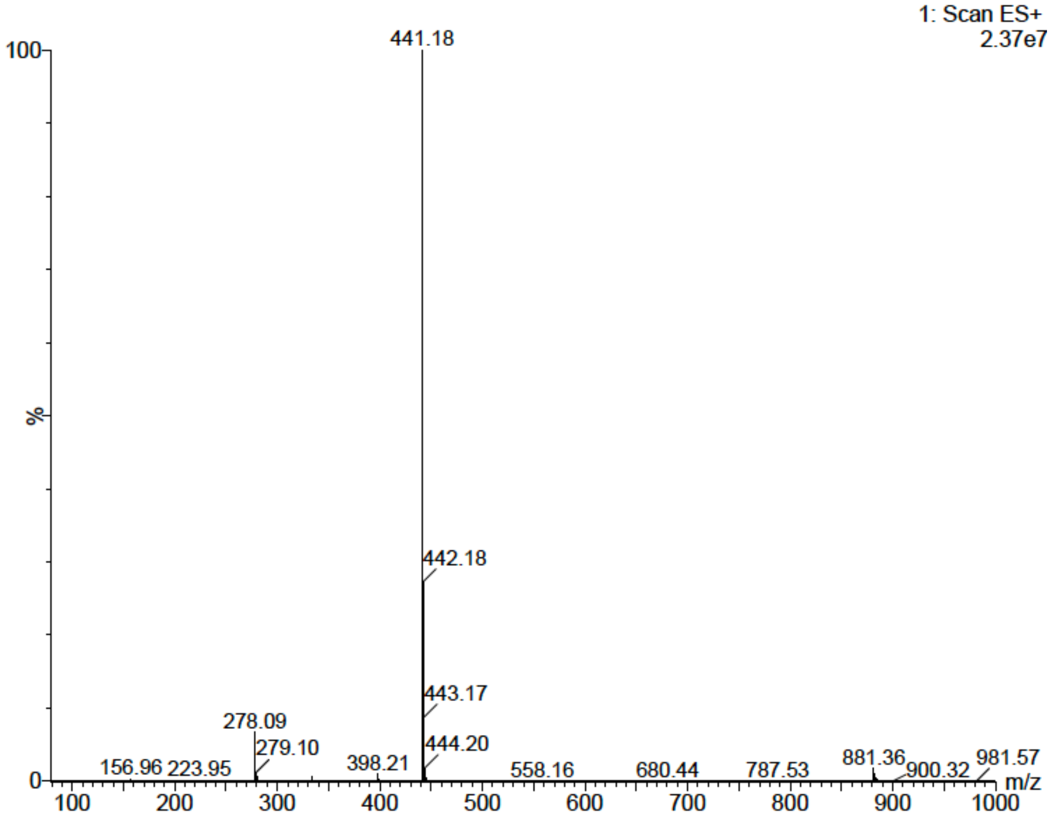

1: Scan ES+
2.37e7
441.18
442.18
443.17
444.20
278.09
279.10
156.96
223.95
398.21
558.16
680.44
787.53
881.36
900.32
981.57
%
100
0
100
200
300
400
500
600
700
800
900
1000
m/z

Compound ***48***

Formula Weight: 424,51596; Exact Mass: 424,156911358; Molecular Formula: $C_{22}H_{24}N_4O_3S$

^{1}H-NMR chemical shifts δ [ppm]

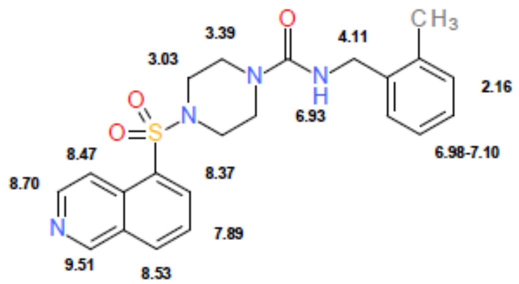

Characterictic *J*(H,H) coupling constants [*Hz*]

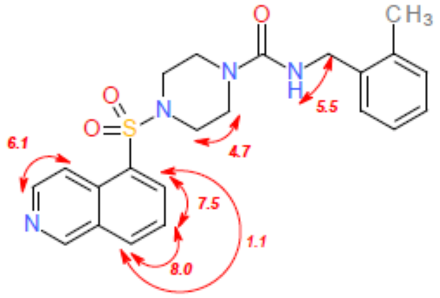

^{13}C-NMR chemical shifts δ [ppm]

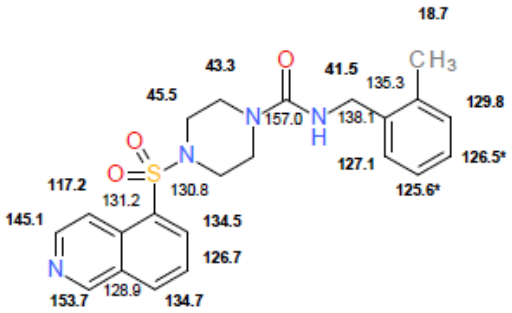

Characteristic heteronuclear long-range couplings detected by HMBC experiment H ⟶ C

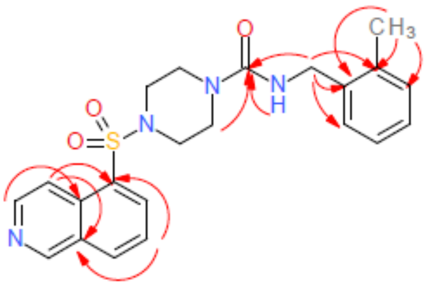

^{1}H NMR:

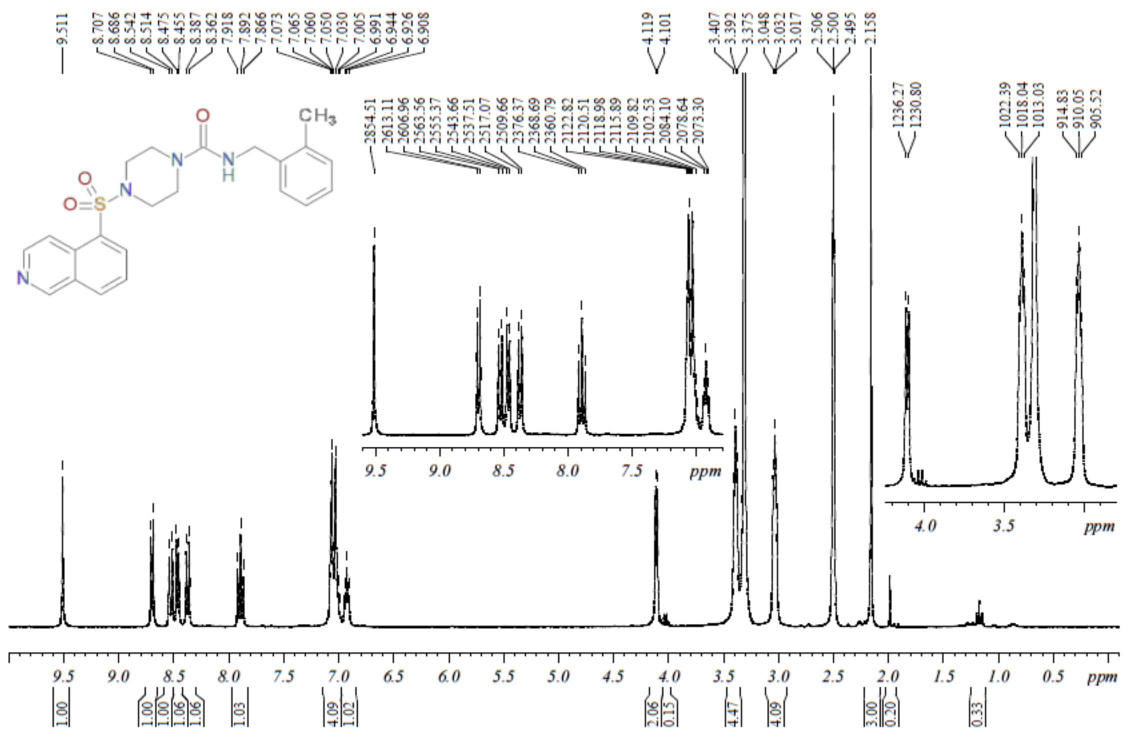

Total correlation spectroscopy (TOCSY):

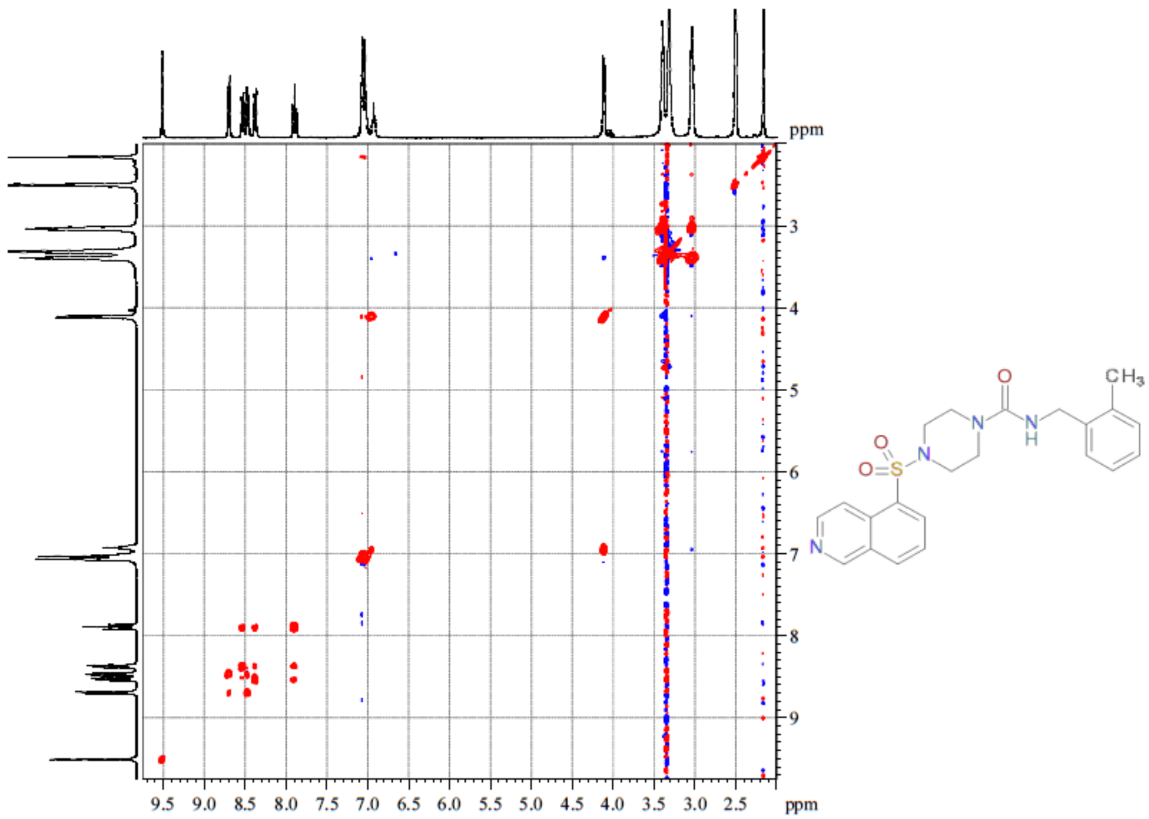

^{13}C-NMR:

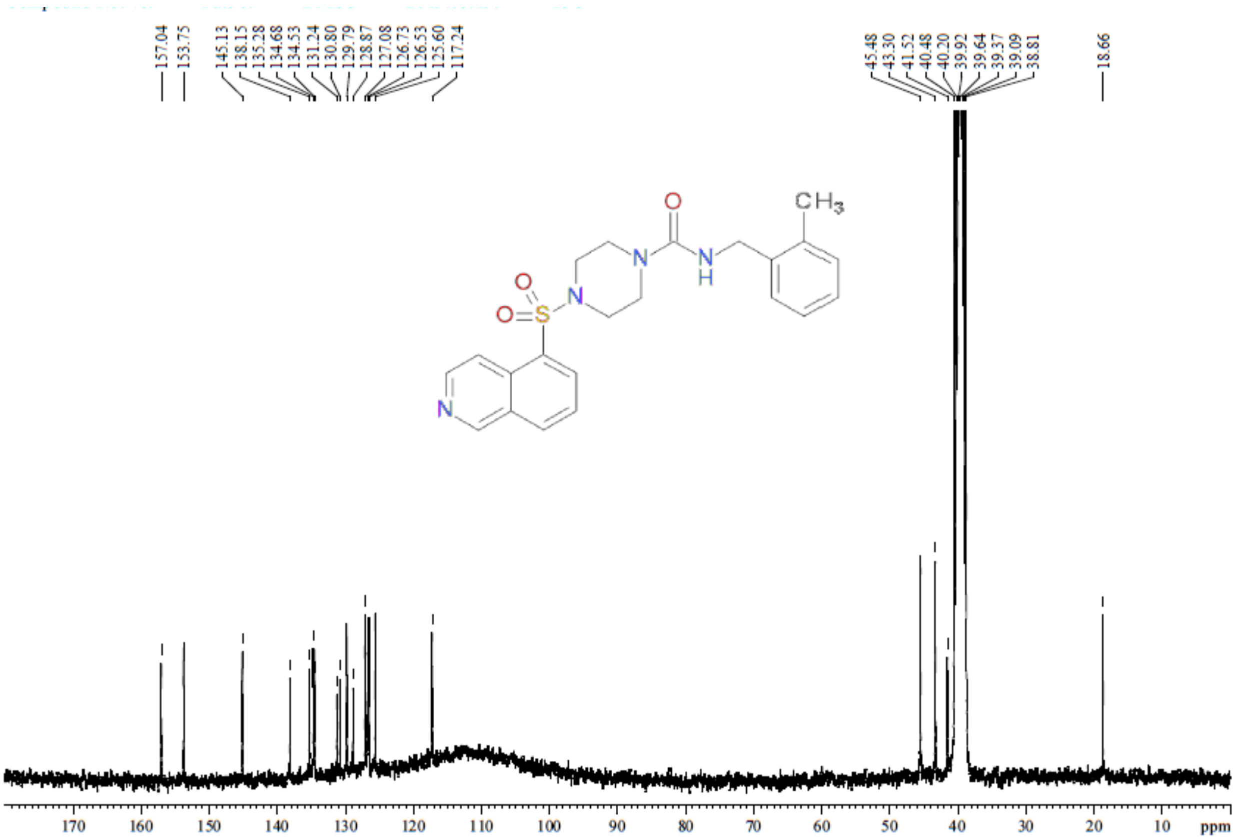

Heteronuclear single quantum coherence (HSQC):

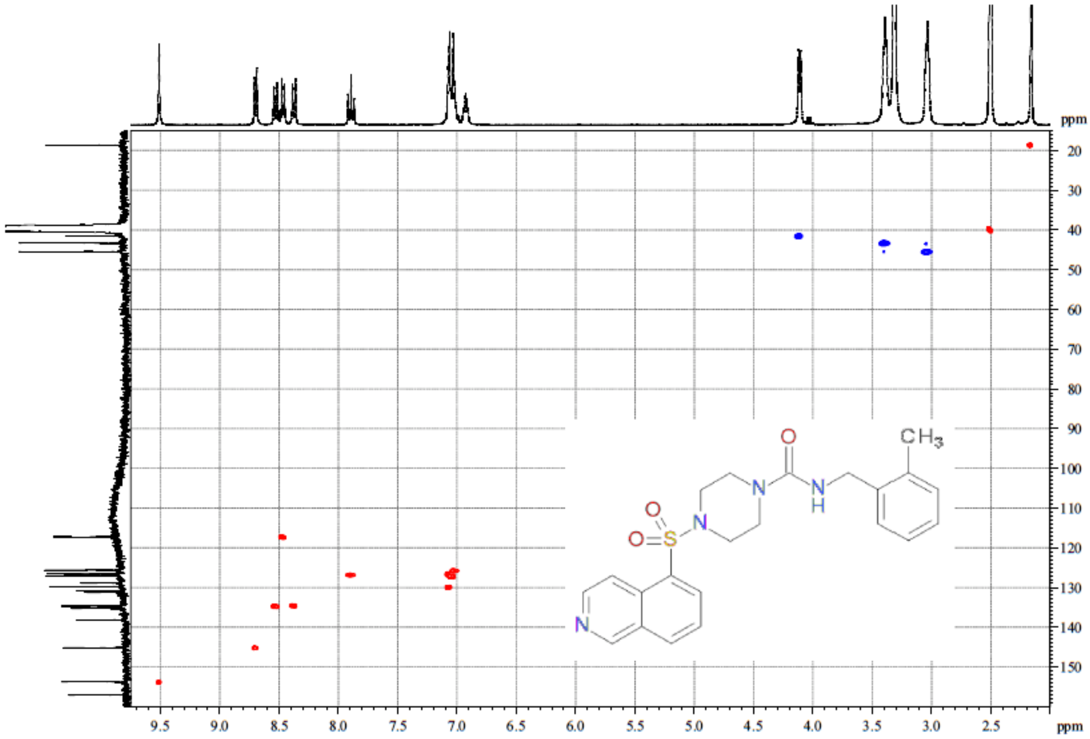

HMBC:

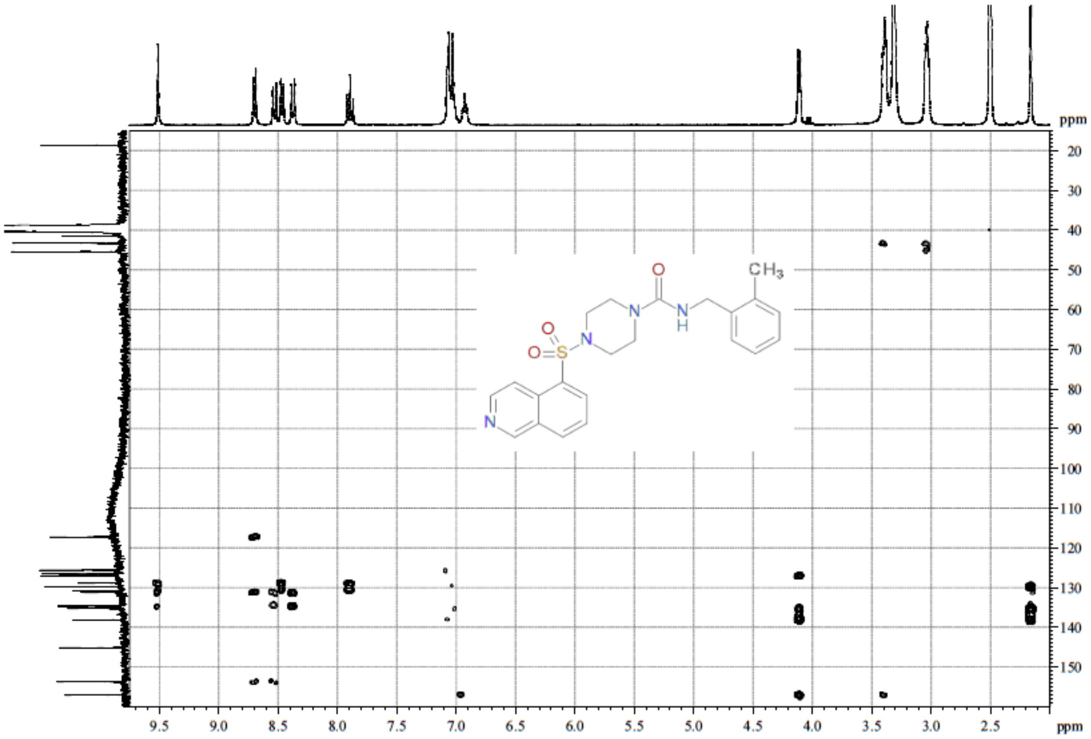

ppm
20
30
40
50
60
70
80
90
100
110
120
130
140
150
9.5
9.0
8.5
8.0
7.5
7.0
6.5
6.0
5.5
5.0
4.5
4.0
3.5
3.0
2.5
ppm
O
CH3
N
N
H
O
S
O
N
N

LC-MS:

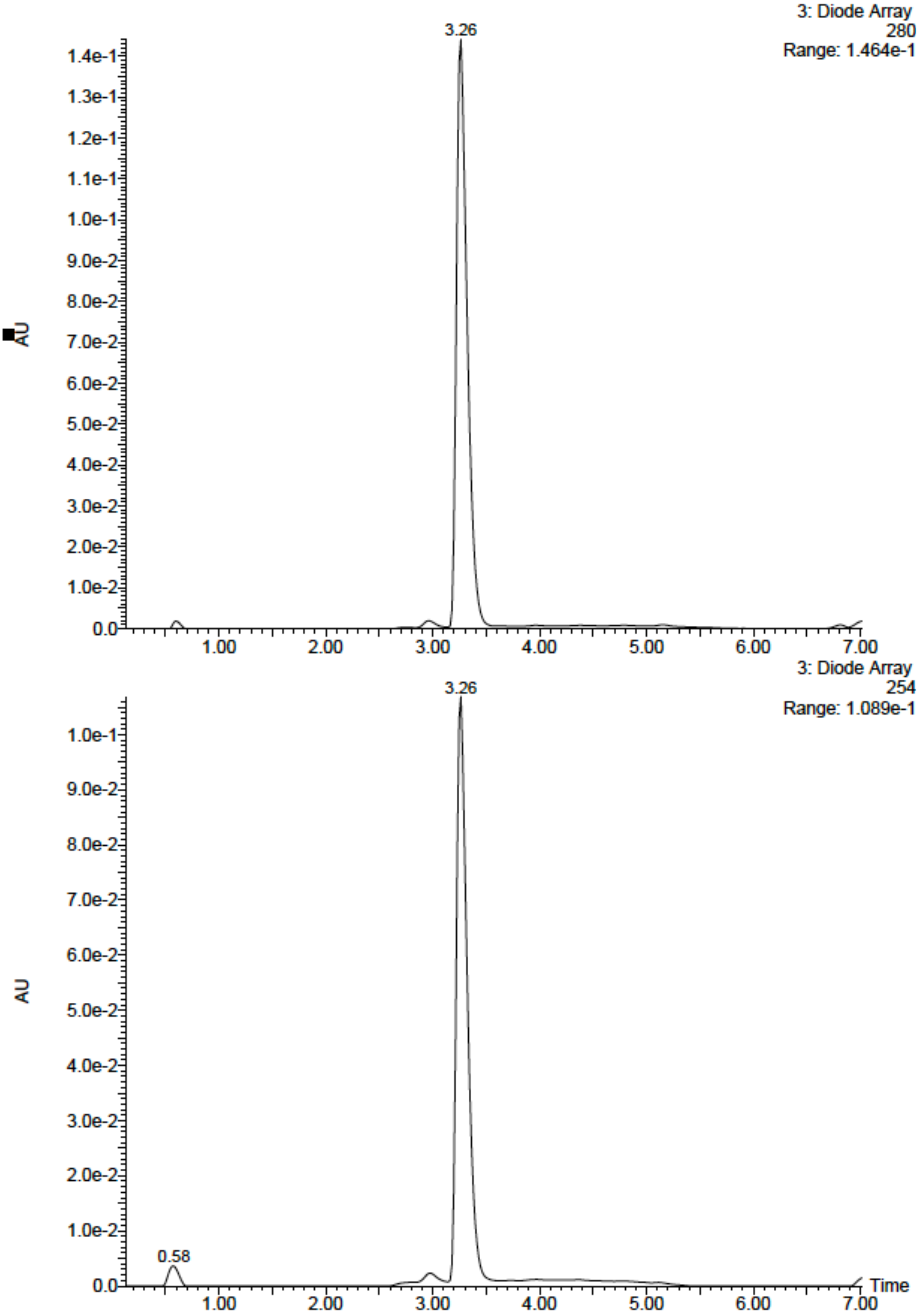
3: Diode Array
280
Range: 1.464e-1
3.26
AU
1.4e-1
1.3e-1
1.2e-1
1.1e-1
1.0e-1
9.0e-2
8.0e-2
7.0e-2
6.0e-2
5.0e-2
4.0e-2
3.0e-2
2.0e-2
1.0e-2
0.0
1.00
2.00
3.00
4.00
5.00
6.00
7.00
3: Diode Array
254
Range: 1.089e-1
3.26
0.58
AU
1.0e-1
9.0e-2
8.0e-2
7.0e-2
6.0e-2
5.0e-2
4.0e-2
3.0e-2
2.0e-2
1.0e-2
0.0
1.00
2.00
3.00
4.00
5.00
6.00
7.00
Time

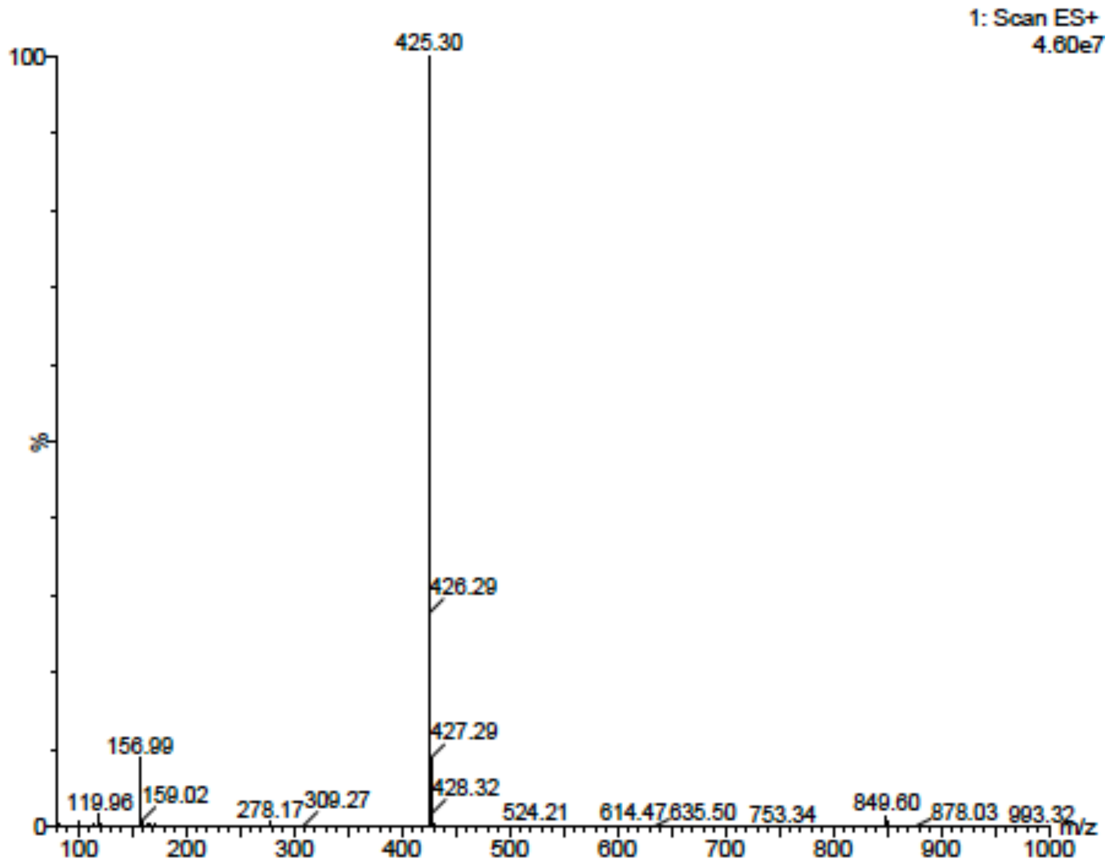

1: Scan ES+
4.60e7
425.30
100
%
426.29
427.29
156.99
428.32
119.96
159.02
278.17
309.27
524.21
614.47
635.50
753.34
849.60
878.03
993.32
0
m/z
100
200
300
400
500
600
700
800
900
1000

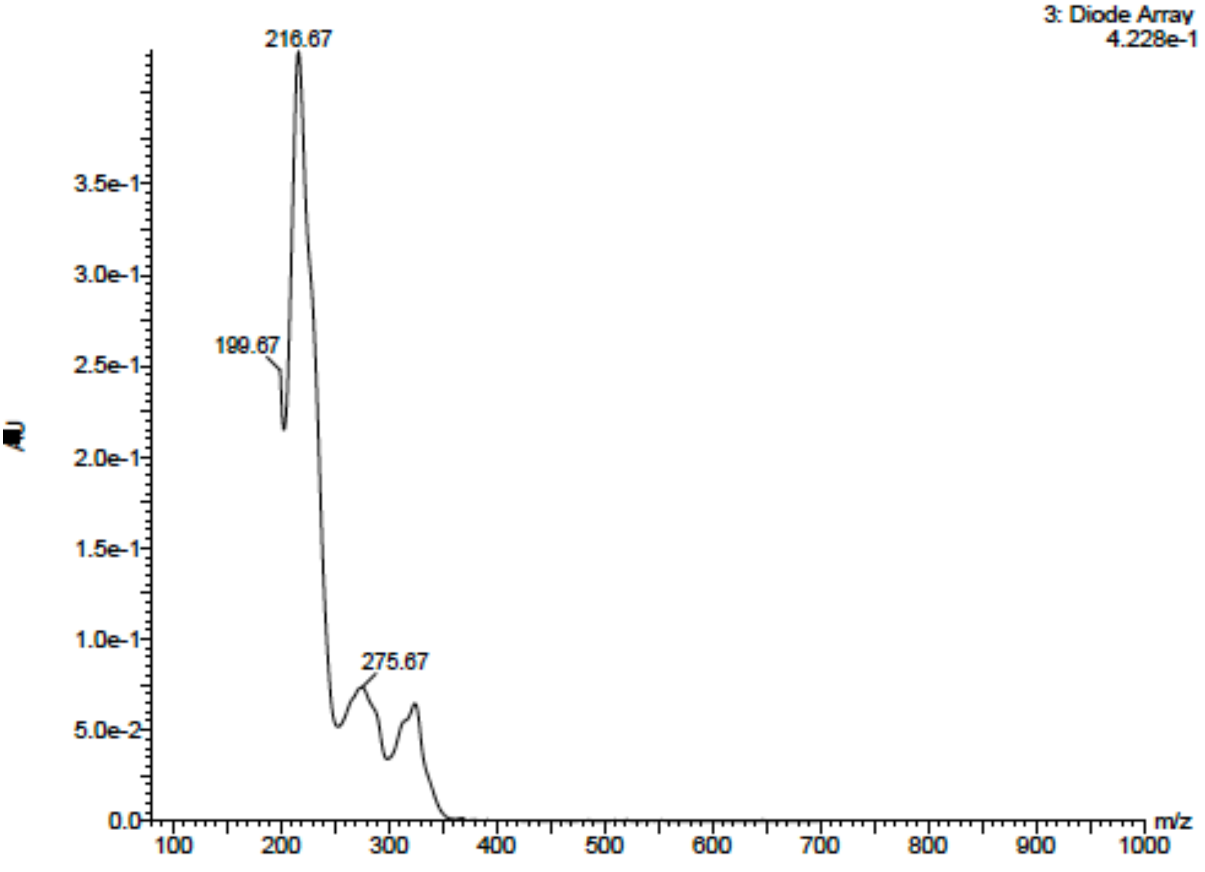

3: Diode Array
4.228e-1
216.67
199.67
275.67
AU
3.5e-1
3.0e-1
2.5e-1
2.0e-1
1.5e-1
1.0e-1
5.0e-2
0.0
m/z
100
200
300
400
500
600
700
800
900
1000
